# ASCENT (Automated Simulations to Characterize Electrical Nerve Thresholds): A pipeline for sample-specific computational modeling of electrical stimulation of peripheral nerves

**DOI:** 10.1371/journal.pcbi.1009285

**Published:** 2021-09-07

**Authors:** Eric D. Musselman, Jake E. Cariello, Warren M. Grill, Nicole A. Pelot

**Affiliations:** 1 Department of Biomedical Engineering, Duke University, Durham, North Carolina, United States of America; 2 Department of Computer Science, Duke University, Durham, North Carolina, United States of America; 3 Department of Electrical and Computer Engineering, Duke University, Durham, North Carolina, United States of America; 4 Department of Neurobiology, Duke University, Durham, North Carolina, United States of America; 5 Department of Neurosurgery, Duke University, Durham, North Carolina, United States of America; Hebrew University of Jerusalem, ISRAEL

## Abstract

Electrical stimulation and block of peripheral nerves hold great promise for treatment of a range of disease and disorders, but promising results from preclinical studies often fail to translate to successful clinical therapies. Differences in neural anatomy across species require different electrodes and stimulation parameters to achieve equivalent nerve responses, and accounting for the consequences of these factors is difficult. We describe the implementation, validation, and application of a standardized, modular, and scalable computational modeling pipeline for biophysical simulations of electrical activation and block of nerve fibers within peripheral nerves. The ASCENT (Automated Simulations to Characterize Electrical Nerve Thresholds) pipeline provides a suite of built-in capabilities for user control over the entire workflow, including libraries for parts to assemble electrodes, electrical properties of biological materials, previously published fiber models, and common stimulation waveforms. We validated the accuracy of ASCENT calculations, verified usability in beta release, and provide several compelling examples of ASCENT-implemented models. ASCENT will enable the reproducibility of simulation data, and it will be used as a component of integrated simulations with other models (e.g., organ system models), to interpret experimental results, and to design experimental and clinical interventions for the advancement of peripheral nerve stimulation therapies.

## Author summary

Despite promising results from preclinical studies, novel therapies using electrical stimulation of peripheral nerves often fail to produce successful clinical outcomes due to differences in neural anatomy across species. These differences often require different electrodes to interface with the nerves and/or different stimulation parameters to achieve equivalent nerve responses. Further, differences in nerve anatomy across a population contribute to differences in nerve responses to stimulation. These inter-species and inter-individual differences can be studied using computational modeling of individual-specific peripheral nerve morphology and biophysical properties. To accelerate the process of computational modeling of individual nerve anatomy, we developed ASCENT, a software platform for simulating the responses of sample-specific nerves to electrical stimulation with custom electrodes and stimulation parameters. ASCENT automates the complex, multi-step process required to build computational models of preclinical and clinical studies and to design novel stimulation protocols using biophysically realistic simulations. The ASCENT pipeline will be used to develop technologies that increase the selectivity and efficiency of stimulation and to accelerate the translation of novel peripheral nerve stimulation therapies to the clinic.

## 3 Introduction

Targeted and reversible changes in nerve activity by electrical stimulation and block of peripheral nerves holds great promise for treatment of disease and injury. For example, vagus nerve stimulation (VNS) is an FDA-approved therapeutic device including a cuff electrode around the left cervical vagus nerve and a pulse generator implanted subcutaneously in the chest. Stimulating the nerve with low frequencies, e.g., tens of Hz, activates the underlying nerve fibers to treat pharmacoresistant epilepsy [1–3] and depression [4–6]. Given the diverse end-organ targets of the vagus nerve, VNS is under investigation for a wide range of diseases [7] including sepsis, fibromyalgia, migraine, cardiovascular disease, ventilator-induced lung injury, stroke, diabetes, and rheumatoid arthritis [8].

Despite promising results from preclinical studies, novel applications of electrical stimulation of peripheral nerves often fail to translate to successful clinical therapies. Differences in neural anatomy across species often require different electrodes to interface with the nerves and/or different stimulation parameters to achieve equivalent nerve responses. Further, differences in nerve anatomy across individuals contribute to differences in nerve responses to stimulation. The nerve morphology, fiber types, and electrode geometry substantially impact the responses of nerve fibers to a given set of stimulation parameters, and producing equivalent responses between species or across individuals remains a challenge. The exceedingly large stimulation parameter space and nonlinear input-output relationships between applied stimulation and neural responses make it unrealistic to develop an optimized intervention using only *in vivo* experiments.

Computational modeling can be leveraged to achieve translation between preclinical and clinical studies and to optimize application-specific parameters. Prior modeling studies used generalized representations of nerve morphology [9–11] or patient-specific models of peripheral nerve stimulation [12,13], but these were bespoke implementations by experts; a standardized platform to enable the research community to implement and use such models has not been developed. The field needs a “completely customizable and modular framework … to interpret the results of stimulation experiments in terms of quantities of common use in neuro-prosthetics” [14].

We describe the implementation, validation, and application of a computational modeling pipeline for simulation of electrical activation and block of populations of nerve fibers within peripheral nerves. ASCENT (Automated Simulations to Characterize Electrical Nerve Thresholds) spans from fixed nerve samples to account for individual-specific neural anatomy (i.e., species and location of intervention), through three-dimensional representations of electrodes positioned on nerves, to analysis of waveforms and electrodes that produce selective activation or block in specific nerve fiber types (S1 Text). ASCENT automates the complex, multi-step process required to model peripheral nerve stimulation and block. The resulting models provide essential tools to aid in the interpretation of experimental results, for design of experimental and therapeutic interventions, and as elements that can relate to other models (e.g., organ system models) for integrated simulations. We applied ASCENT to quantify changes in fiber thresholds for different cuff electrode positions on a multifascicular nerve; we reproduced a published model of rabbit sciatic nerve stimulation and we improved upon a published model of human VNS, demonstrating how ASCENT reduces duplication of efforts and serves as a standardized platform to enable model reproducibility and promote best practices; and we modeled dog VNS experiments, reproducing the *in vivo* activation thresholds. Thus, ASCENT provides an important contribution towards FAIR (findable, accessible, interoperable, reusable) data in the realm of computational modeling [15].

## 4 Design and implementation

### 4.1 Overview of the ASCENT pipeline

**Fig 1:**
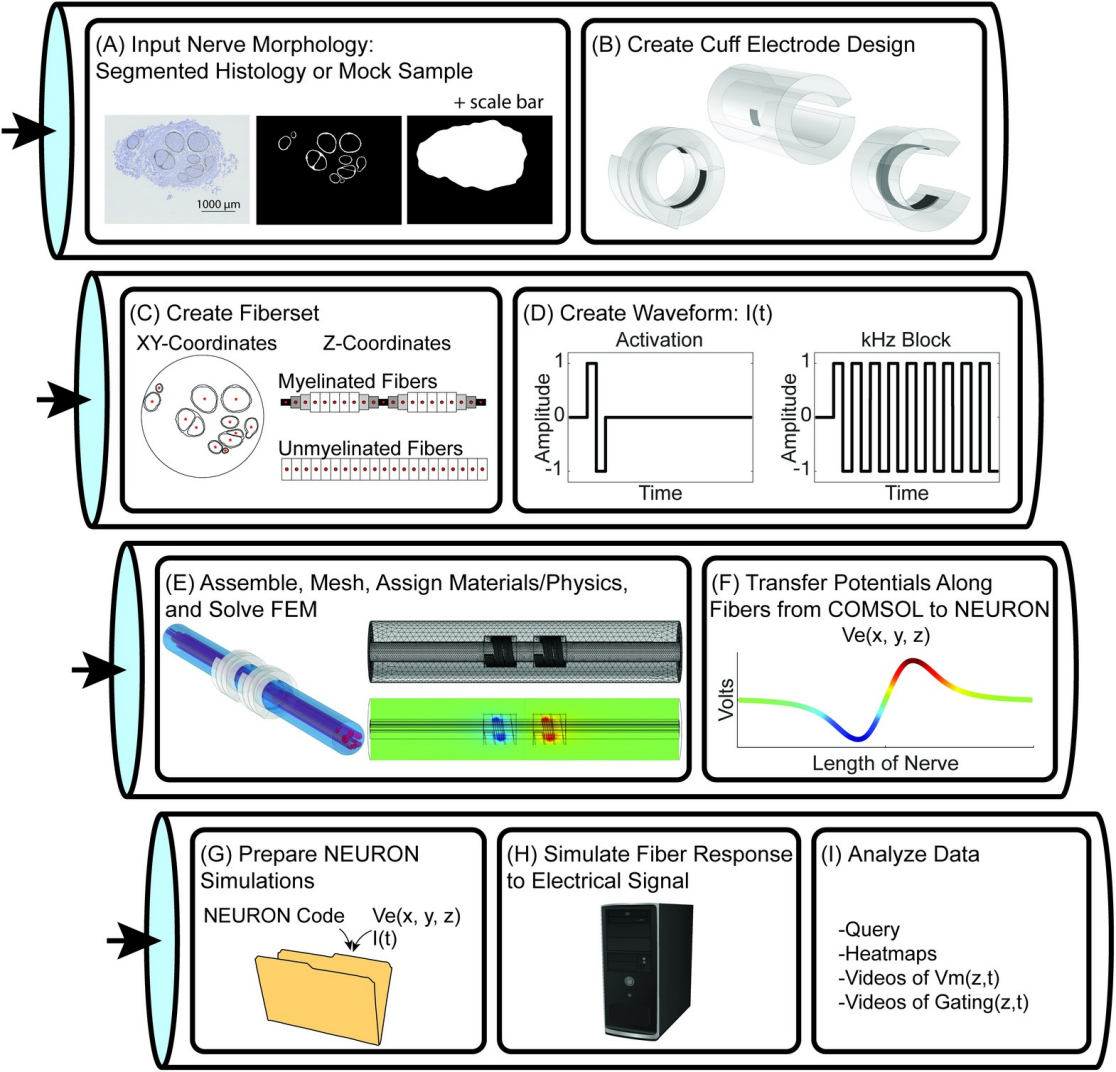
Overview of the ASCENT pipeline for simulating the response of sample-specific nerves to electrical stimulation with custom cuff electrodes and stimulation waveforms. FEM: finite element model.

ASCENT computes the electric fields [16] generated in inhomogeneous anisotropic nerve tissue by one or multiple current sources and applies the resulting extracellular potentials to cable models of individual fibers as a time-varying signal [17] (Fig 1). ASCENT’s process minimizes duplication of computationally intensive tasks, enables scalability by preserving modularity of operations, and provides robust automation and user control. Furthermore, ASCENT enables automatic batching of model execution across all domains (e.g., nerve samples, electrode geometries and positions on the nerve, material and tissue properties, fiber models, stimulation waveforms), which is essential for model-based design of electrodes and stimulation paradigms. ASCENT has built-in control for modular runs, error handling to skip failed models, breakpoints to run only up to a specified process, and scripts for accessing and plotting intermediate data (e.g., traces of perineurium and epineurium tissue boundaries, fiber locations, and stimulation waveforms).

The pipeline uses Python 3.7 [18], Java SE Development Kit 8 (1.8) [19], COMSOL Multiphysics 5.5 (COMSOL Inc., Burlington, MA), and NEURON v7.6 [20]; see S2 Text for installation instructions, S3 Text for a description of the data hierarchy, S4 Text for instructions to complete your first run of the pipeline, and S5 Text for guidelines to report methods. After defining parameters in JSON configuration files (S6–S9 Text), the user initiates two sequential processes: the first process uses Python, Java, and COMSOL to create stimulation waveforms and extracellular potentials along the length of fibers, and the second process uses Python to prepare batched NEURON jobs for execution on a personal computer or computer cluster (S10 Text).

### 4.2 Digitized nerve morphology

The finite element model (FEM) of a nerve can be sample-specific using segmented histology (S11 Text) [21] or generalized using our mock nerve morphology generator, as described below (S12 Text). We developed a standardized object-oriented framework for representing and processing nerve morphology in Python (S13 Text). From input binary masks, Python operations prepare the nerve sample for placement in a cuff electrode, including optional correction for tissue shrinkage during histology and deformation of the nerve to conform to the inner diameter of a cuff; Python produces two-dimensional CAD files to define nerve and fascicle tissue boundaries in COMSOL (S14 Text).

#### 4.2.1 Segmented histology

**Fig 2:**
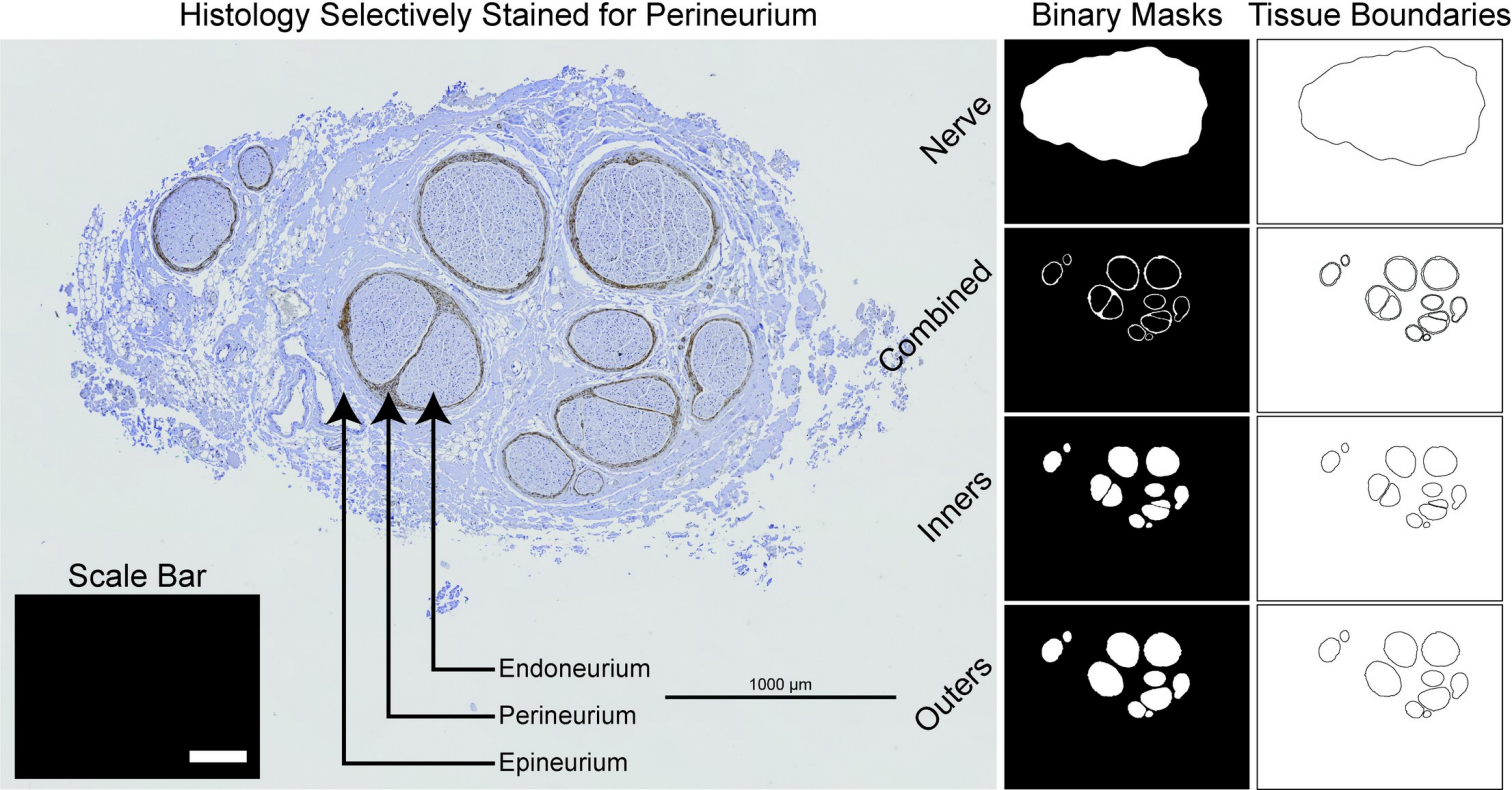
Define sample-specific models with segmented nerve images. We segmented the nerve boundary (n.tif), the perineurium (either “Combined” (c.tif), or both “Inners” (i.tif) and “Outers” (o.tif), or only “Inners”), and a horizontal “Scale Bar” (s.tif) of known length from a micrograph of a human cervical vagus nerve stained selectively for perineurium. In this example, two fascicles have multiple inner traces for their single outer trace (i.e., “peanut” fascicles), which requires their perineurium to be meshed; conversely, the other fascicles have a single inner trace for each outer trace, in which case the perineurium can be either meshed or modeled with a thin layer approximation. The nerve cross section is modeled in the (x,y,0) plane and extruded into the +z direction for the length of the nerve.

A segmented cross section of a nerve sample can be used to build the FEM (Figs 1A and 2).

#### 4.2.2 Mock nerve morphology generator

Alternatively, users can define a nerve sample as an elliptical nerve and a list of elliptical fascicles using our mock nerve morphology generator. The user may place fascicles using either the *explicit* option, for which the sizes (lengths of the major and minor axes), locations (centroids), and rotations of fascicles are explicitly defined by the user, or the *probabilistic* option, which randomly places a user-defined number of elliptical fascicles within the nerve: diameters and eccentricities are drawn from a statistical distribution (e.g., truncated normal, uniform), centroids are randomly chosen using disk point picking, and rotations are drawn from a uniform distribution. In randomly placing fascicles, the overlap of fascicles of fixed size and rotation is avoided by iteratively redefining the centroid of each fascicle until it is placed such that its boundaries are at least a user-defined distance from neighboring nerve and previously populated fascicle boundaries; the program will attempt to place each fascicle a user-defined number of times before skipping the fascicle and reporting the progress to the user.

### 4.3 Cuff electrode design

**Fig 3:**
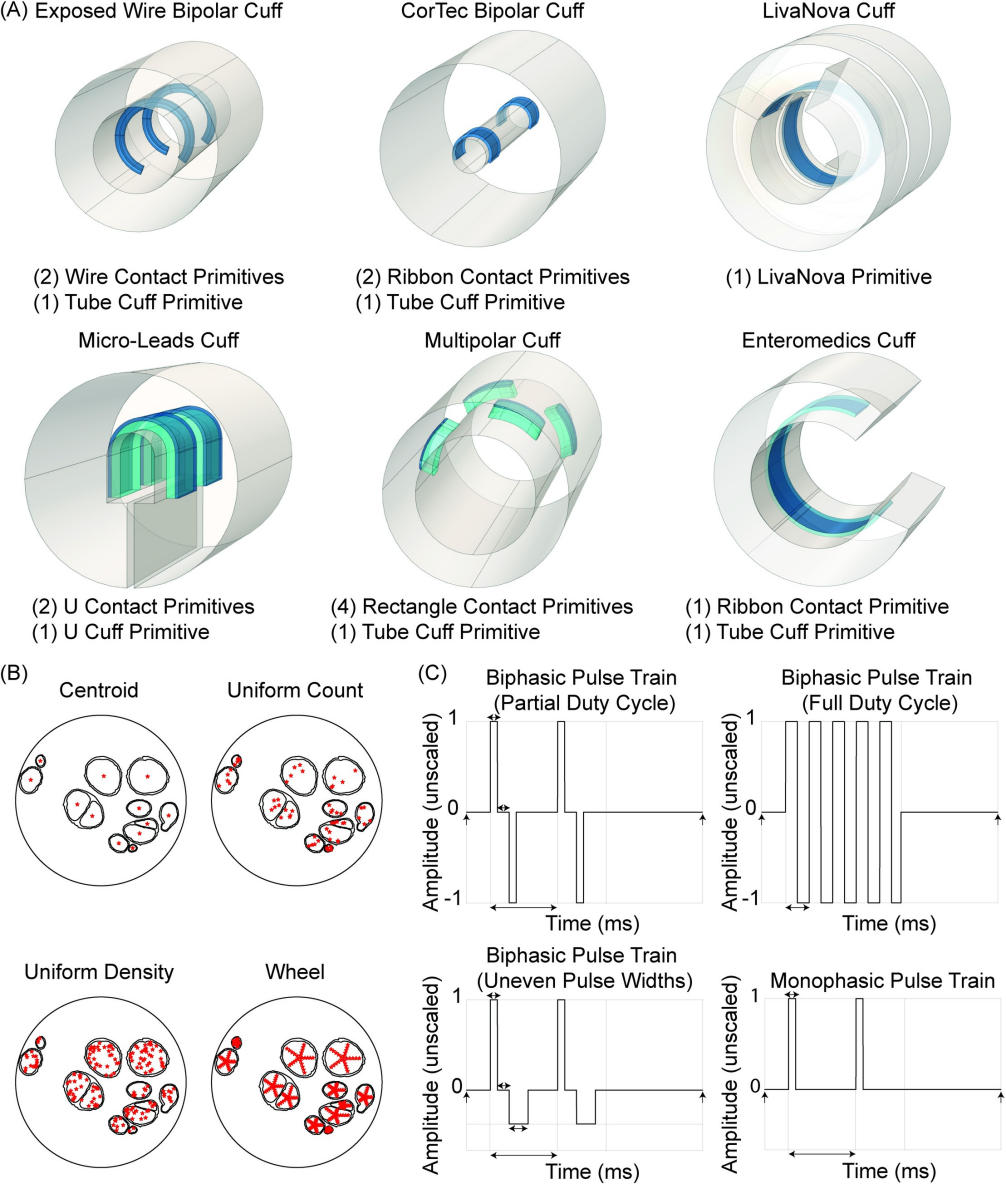
Parameterized preset cuff electrodes, modes to define fiber locations in nerve cross section, and modes to define conventional stimulation waveforms. (A) Examples of “preset” cuffs commonly used in preclinical and clinical studies assembled using part primitives from S16 Text, which are provided in config/system/cuffs/. Custom cuff electrodes can be created from existing or new part primitives (S17 and S18 Text). (B) Types of FiberXYMode defining the (x, y)-coordinates of the fiber locations in the nerve cross section. The parameters needed to define the (x, y)-coordinates for each “FiberXYMode” are explained in S8 Text. (C) Parameterized definitions of conventional waveforms included in the ASCENT pipeline. Users can also define custom waveforms (S8 Text).

ASCENT includes tools to define and manipulate the representation of cuff electrodes and surrounding media in COMSOL (Fig 1B). The pipeline includes cuff electrodes commonly used in preclinical studies and clinical therapies (Fig 3A and S15 Text). Users can also assemble custom cuff electrodes by adding parameterized instances of “part primitives” for electrode contacts, cuff insulators, and cuff fill (between the nerve and cuff and/or in the immediate vicinity around the cuff; e.g., saline, mineral oil, or encapsulation tissue) (S16–S18 Text). The user can control the placement of the cuff with respect to the nerve, including translation and rotation (S19 Text).

### 4.4 Fiber locations and waveforms

Before interfacing with COMSOL via Java, Python defines the coordinates of “fibersets” (i.e., the coordinates where voltages are sampled in COMSOL for extracellular stimulation or block of model fibers) (Fig 3B) and defines the current amplitude versus time stimulation waveforms used in NEURON (Fig 3C).

#### 4.4.1 Create fiberset

ASCENT includes parameterized modes to define fiber locations (Figs 1C and 3B and S20 Text). At each fiber’s (x, y)-location in the nerve cross section, potentials are sampled along the z axis at the locations of compartments of published models of myelinated and unmyelinated fibers (S21 Text). Alternatively, users can “super-sample” the potentials along the length of the nerve in COMSOL at each (x, y)-location: the pipeline will interpolate the potentials for the spatial discretization of each fiber model to be simulated, thereby eliminating later dependency on COMSOL.

#### 4.4.2 Create waveforms

The pipeline provides parameterized definitions of conventional waveforms (Figs 1D and 3C), defined using the same timestep and duration as the NEURON simulation. We use the SciPy Signal package [22] and the NumPy library [23] to generate the conventional waveforms. Users can also define custom arbitrary waveforms. The stimulation waveform input to NEURON is “unscaled”, meaning that the maximum magnitude is one; the waveform delivered to the model fibers in NEURON is then scaled by the stimulation amplitude (S22 Text).

### 4.5 Finite element modeling

After creating nerve morphology CAD files, fibersets, and waveforms in Python, a Java subprocess interfaces with COMSOL to build, mesh, solve, and extract data from FEMs (Fig 1E and S23 and S24 Text).

#### 4.5.1 Assemble and mesh the FEM

The medium surrounding the nerve and cuff electrode is represented by a cylindrical domain (S25 Text). The user can define different meshing parameters for the “proximal” domains (i.e., a cylinder containing the full length of the nerve and the cuff electrode) and the remaining “distal” domains; thus, the spatial discretization may be defined more coarsely outside of the target nerve area. The pipeline builds an unstructured mesh with tetrahedral elements and reports the meshing statistics (quality, number of elements, meshing time). Since files of FEM meshes can be quite large, we provide the option to save them; the pipeline will reuse a previously saved FEM mesh to reduce computation time if the current model has the identical geometry and mesh parameters, particularly in sweeps of material properties and boundary conditions (S26 Text).

#### 4.5.2 Assign conductivities and sources to the FEM

After the geometry of the model is discretized with a mesh, the pipeline applies the conductivities of the materials (cuff “insulator”, contact “conductor”, contact “recess”, cuff “fill”) and tissues (surrounding medium, endoneurium, epineurium, perineurium) to their corresponding domains. Relevant material and tissue conductivities from literature are built into ASCENT, and users may opt to define their own materials [24–31] (S27 Text). Perineurium can be modeled as a meshed domain or with a thin layer approximation to reduce mesh complexity (except with “peanut” fascicles; see an example in Fig 2 where the latter is termed “contact impedance” in COMSOL [27] (S28 Text).

A point source of current is placed at the center of each contact. A grounded boundary condition (V = 0) is applied to the outer-most surfaces of the surrounding medium, which includes the ends of the nerve, to simulate a distant ground (e.g., implanted pulse generator for chronic studies, percutaneous needle for acute experiments). However, if stimulating with more than one contact where the sum of the currents across the contacts is zero (e.g., bipolar cuff with +1 mA at one contact and -1 mA at the other), the current sunk to or sourced from the ground will be effectively zero after the difference is taken between the potentials resulting from each contact’s FEM solution. Alternatively, users may use an insulating boundary condition on the most distant surfaces.

#### 4.5.3 Solve the FEM

The pipeline solves the FEM once for each contact, where each contact is assigned 1 mA and the other contacts are floating [32]; this defines the solution “bases”. The potentials are obtained using conjugate gradients to solve Laplace’s equation using second order solution and geometry shape functions:

∇·(σ·∇ϕ)=0 (1)

Since files of FEM solutions can be quite large, we provide the option to save them; subsequent simulations of fibers within the same FEM but with new waveforms, a different ratio of currents across the contacts, or different fiber models can use either previously saved COMSOL files (*.mph files in bases/) or previously super-sampled potentials (*.dat files in ss_bases/).

#### 4.5.4 Transfer potentials from COMSOL to NEURON

The pipeline samples the potentials at each coordinate (x, y, z) of each fiber within fibersets/ or ss_coords/ (i.e., “super-sampled” coordinates) using the COMSOL Java API for each of the solution bases (Fig 1F and S29 Text). The linearity of scaling from Ohm’s law indicates that we can compute the potentials generated by any weighting of active contacts (i.e., different current amplitudes delivered by different contacts) by the dot product of our “bases” with a vector of contact weights.

### 4.6 Simulating fiber responses to electrical signals

When all potentials from COMSOL are saved to file, the pipeline returns to Python to build NEURON simulation directories (i.e., n_sims/) (Fig 1G and S30 Text) to be submitted to a computer or computer cluster using the submit.py or submit.sh scripts (Fig 1H and S10 Text). Users can simulate activation thresholds, block thresholds, or responses to set stimulation amplitudes (S22, S31 and S32 Text) for the MRG (McIntyre-Richardson-Grill) myelinated fiber models [33,34], the Rattay and Aberham, Sundt et al., and Tigerholm et al. unmyelinated [35–37] fiber models, or user-defined fiber models.

### 4.7 Data analysis

We provide tools for selecting and loading data created by the pipeline for analysis and visualization. We provide example scripts to plot inputs (e.g., nerve morphologies, fiber locations and discretization, waveforms) and outputs (e.g., heatmaps of thresholds) (Fig 1I and S33 Text). We also provide code to generate videos of transmembrane potential or ion channel state variables (i.e., gating parameters) as functions of space and time.

### 4.8 User oversight

Although ASCENT has built-in exception checking for many invalid parameter cases, the user is ultimately responsible for confirming that the chosen parameters are appropriate. As with all computational modeling, it is possible to obtain results (i.e., without the pipeline throwing exceptions) but for the results to be incorrect. In addition to checks around trends in the expected outputs, the user can leverage ASCENT tools to verify inputs and intermediate data.

We recommend that users plot their waveforms and fiber locations using the provided scripts (S33 Text). Additionally, we recommend that users verify that the FEM geometry is as expected in the COMSOL GUI with materials, boundary conditions, and physics assigned to the correct components; modular runs (e.g., only building the nerve) and partial runs (i.e., adding breakpoints to run only up to a specified step) (S8 Text) are useful for checking the simulations at multiple levels. Users must also conduct convergence studies to ensure that parameters are appropriately defined for sufficient numerical accuracy of the target outcomes (e.g., thresholds), including the size (i.e., length and radius) of the surrounding medium, the length of the fibers, the resolution of the mesh, and the time step (S34 Text) [38]. Users must also verify that the location of action potential detection is sufficiently far from the electrode cuff such that propagating activity is detected rather than only an ohmic rise in transmembrane potential, and thus, they must also use sufficient simulation time for any action potentials to propagate from the initiation site to the recording site.

## 5 Results

We applied ASCENT’s extensive set of features to quantify the change in fiber responses across cuff rotations for a multifascicular nerve, to implement previously published models of rabbit sciatic [11] and human vagus [39] nerve stimulation, and to reproduce findings from an *in vivo* study of dog VNS [40].

First, we designed test simulations to verify the accuracy of ASCENT’s activation thresholds. The verifications were conducted in collaboration with The Foundation for Research Technologies in Society (IT’IS) with the Sim4Life simulation platform (https://zmt.swiss/sim4life/) (Zurich, Switzerland) (S35 Text). We found strong agreement in thresholds between models generated with ASCENT and Sim4Life. Cross-validation of thresholds was performed for a monofascicular rat nerve with a bipolar cuff (<4.2% difference in thresholds), an idealized multifascicular nerve with a bipolar cuff (<3% difference), and a multifascicular human nerve with a bipolar LivaNova helical coil cuff (<2.5% difference). We verified ASCENT’s usability by asking beta testers to perform a task aided by supporting documentation (S4 Text).

### 5.1 Investigating impact of cuff rotation on multifascicular nerve

**Fig 4:**
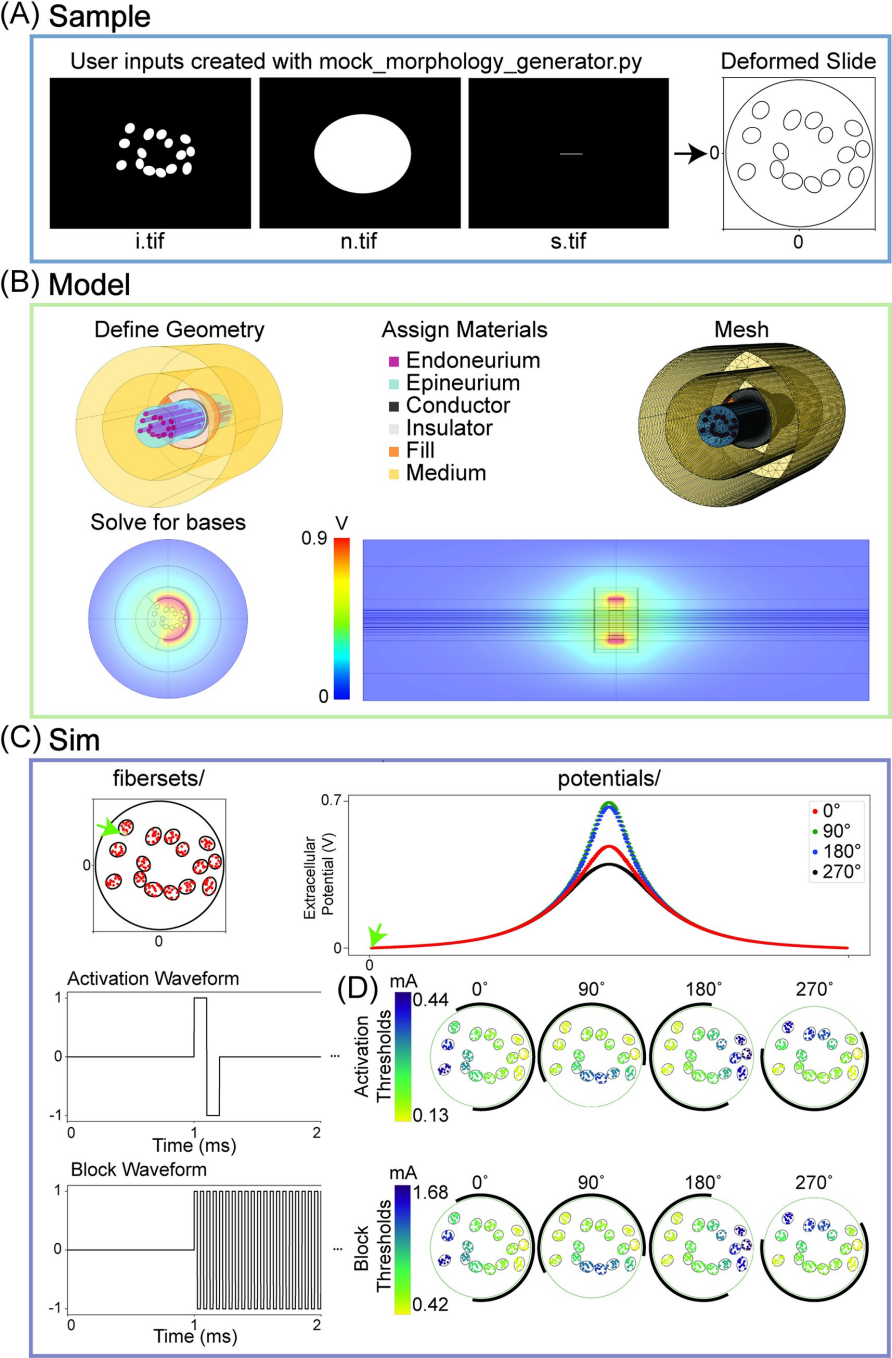
Modeling a multifascicular nerve with different cuff rotations to illustrate key ASCENT processes in context of configuration data hierarchy. (A) Binary images of the fascicle boundaries (“inner” perineurium: i.tif) and nerve boundary (n.tif) to serve as inputs to the pipeline (left) and the resulting deformed traces for placement in a circular cuff electrode (right) following physics-based Python operations. The sample JSON configuration used is provided in examples/results/cuff_rotation/mock.json along with the binary images shown. (B) COMSOL FEM solved for the potentials generated by 1 mA delivered through the monopolar Enteromedics cuff. A contact impedance describes the perineurium ensheathing each fascicle (not shown). Given that it is a monopolar cuff, there is only one solution basis for a given cuff placement; a given ***Model*** will result in a basis solution for each contact, i.e., a basis *.mph file for each contact delivering 1 mA. (C) Inputs to NEURON simulations for activation and block: extracellular potentials (i.e., potentials/ sampled from bases/ corresponding to coordinates defined by fibersets/) and waveforms. The green arrow in the fibersets/ panel points to the (x,y,0)-location for the fiber potentials plotted along the length of the nerve in the potentials/ panel. (D) Heatmaps of activation and block thresholds for 10 μm diameter MRG fibers for four different cuff rotations on a multifascicular nerve. The black arc around the nerve represents the exposed contact length delivering current on the inside surface of the Enteromedics cuff. The heatmaps were generated using Query’s heatmaps() method.

ASCENT can be used to design cuff geometries prior to fabrication and to investigate the sensitivity of activation patterns to cuff geometry and placement. As an example, we simulated the effects of the rotation of a monopolar cuff electrode on thresholds for activation and block of 10 μm diameter myelinated nerve fibers in a multifascicular nerve. Using the mock_morphology_generator.py script, we created binary image masks of the inners (i.tif) and nerve (n.tif) (Fig 4A). The ***Model*** configuration file contains parameters that define the geometry, materials, and physics of the FEM, as well as the settings for model meshing and solving (Fig 4B and S8 Text). The “preset” cuff parameter in ***Model*** for this example is the monopolar Enteromedics cuff. To simulate thresholds for four different cuff orientations on the nerve, we created four ***Model*** configurations with distinct “add_ang” parameter values (0°, 90°, 180°, and 270°) (provided in examples/results/cuff_rotation/ as model_<angle>.json).

We extracted potentials from the four FEM solutions (i.e., one ***Model*** for each cuff rotation) at the coordinates defined by fibersets/ to create potentials/ (Fig 4C). The ***Sim*** configuration files (provided in examples/results/cuff_rotation/ as sim_activation.json and sim_block.json) contain parameters to define fibersets/ for the (x,y)-coordinates (UNIFORM_DENSITY), z-coordinates (compartment spacing for the 10 μm diameter MRG fiber), stimulation waveforms (activation: BIPHASIC_PULSE_TRAIN; block: BIPHASIC_FULL_DUTY), and NEURON simulation control parameters for determining thresholds.

The binary searches to determine activation and block thresholds are conducted in NEURON for each of the four ***Model*** (i.e., four cuff rotations) and two ***Sim*** (i.e., activation and block) configurations. The resulting thresholds are shown as heatmaps in Fig 4D. The results demonstrate that, for this cuff design, cuff rotation on the nerve can substantially impact the distribution of thresholds for activation and block. Thresholds were lowest for fibers closest to the contact. Thus, the cuff rotation changed thresholds for a fiber by up to ~4x, which, depending on the targeted physiological response, could be perceived as a feature or flaw of the cuff’s design.

### 5.2 Reproducing a computational model

ASCENT will reduce duplication of efforts in the computational modeling research community and serve as a standardized platform to enhance model reproducibility. As an example, we implemented models from a published study that used computational modeling and in vivo electrophysiology to compare vagus nerve and sciatic nerve responses across cuff electrode geometries, with the objective of improving efficiency of fiber recruitment [11].

**Fig 5:**
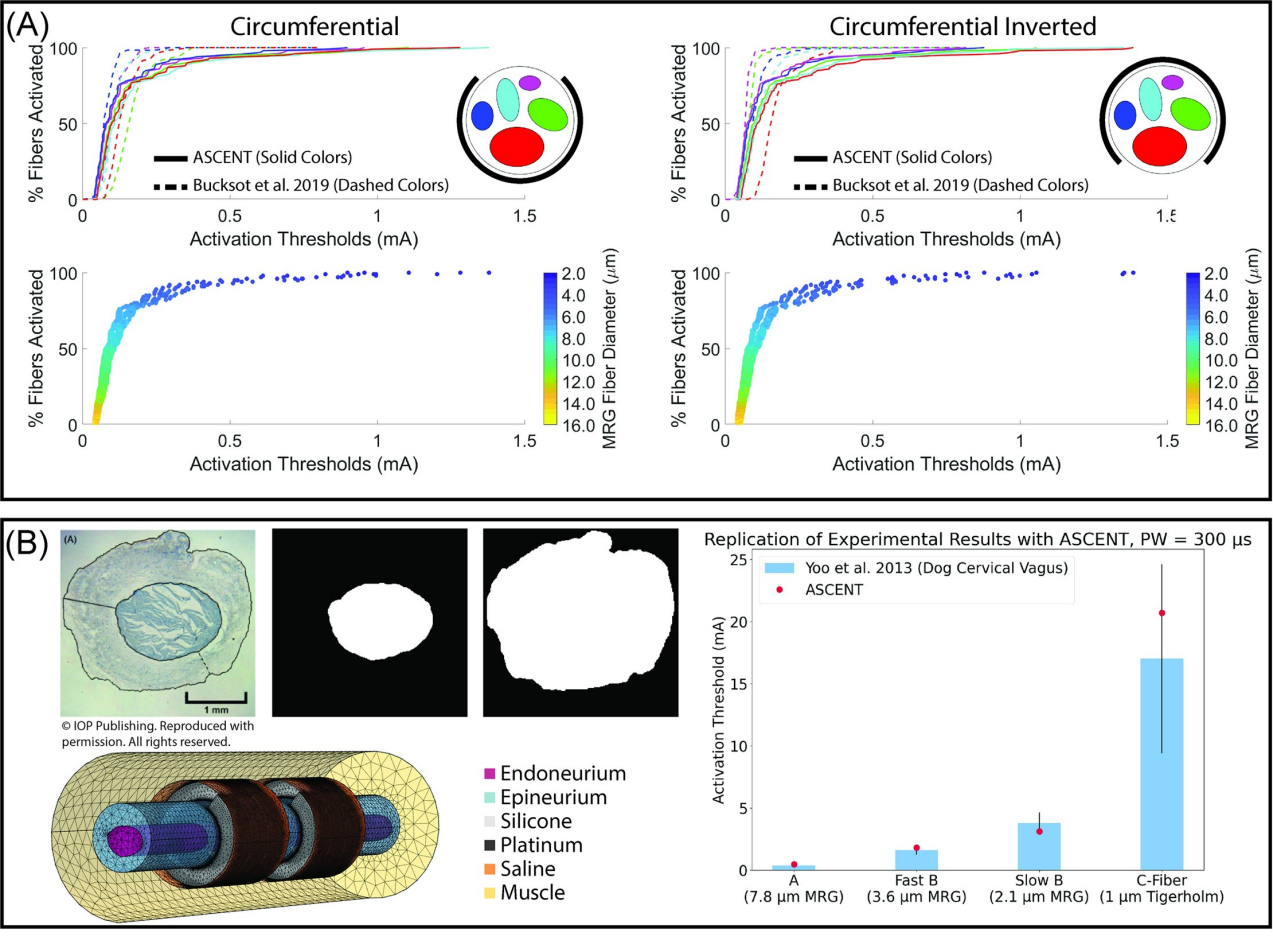
Using ASCENT to replicate previously published computational models and to model an *in vivo* experiment. (A) Recruitment curve for each fascicle in a model of the rabbit sciatic nerve for two rotations of a bipolar cuff electrode [11]. Fiber thresholds are in response to a 100 μs per phase biphasic rectangular pulse (i.e., BIPHASIC_PULSE_TRAIN). We simulated mammalian myelinated fibers at 0.5 μm diameter increments from 2 to 16 μm (i.e., MRG_INTERPOLATION) with 10 fibers of each diameter at consistent locations in each fascicle (i.e., UNIFORM_COUNT). We generated fiber recruitment curves by defining a population of fiber diameters from a normal distribution (8.85 ± 3.1 μm, 100 fibers per fascicle) and rounded each fiber diameter to the nearest 0.5 μm to draw from our modeled activation thresholds randomly. Configuration and input files used to replicate the results are provided in examples/results/bucksot_2019/. (B) Using ASCENT to model electrophysiology study of dog vagus stimulation [40]. We used Photoshop (Adobe Inc., San Jose, CA) to segment a micrograph from one of the animals in the original publication. We placed the nerve in a 2 mm diameter LivaNova helical cuff electrode delivering a 300 μs monophasic rectangular pulse (i.e., MONOPHASIC_PULSE_TRAIN), as used experimentally. We computed activation thresholds for myelinated (i.e., MRG_INTERPOLATION: 2.1, 3.6, and 7.8 μm diameter) and unmyelinated (i.e., TIGERHOLM: 1.0 μm diameter) fiber models, corresponding to the fiber types reported in the original study. We report the modeled threshold of a single fiber of each type at the centroid of the fascicle (i.e., CENTROID) (red dot) overlaid on the *in vivo* ENG thresholds (blue bars with black error bars: mean ± SD) of both myelinated (n = 5 dogs) and unmyelinated (n = 4 dogs) vagus nerve fibers. Our prior modeling studies show that fibers of a consistent diameter placed in the same fascicle have nearly identical thresholds [10]. Configuration and input files used to model the experiment are provided in examples/results/yoo_2013/.

We used ImageJ’s “Analyze Particles” tool [41] to determine the best-fit ellipses of the rabbit sciatic nerve modeled in Bucksot et al. 2019 [11], which we used to define binary images of tissue boundaries using our mock_morphology_generator.py script. We reproduced the electrode geometry, material conductivities, nerve fiber models, and stimulation waveform described in the publication. ASCENT produced fiber recruitment curves that demonstrate preferential recruitment of larger diameter fibers in fascicles that are smallest and closest to the electrode contact (Fig 5A). We obtained similar activation thresholds to the data published by Bucksot et al. 2019, but they were not an exact match due to the incomplete and inconsistent description of model parameters in the publication [11]. Furthermore, ASCENT’s interpolation of the MRG fiber model differs from that in the original publication, resulting in higher thresholds for small diameter myelinated fibers, given our interpolation’s shorter internodal and paranodal lengths (S36 Text).

### 5.3 Modeling *in vivo* vagus nerve stimulation experiment

ASCENT can build biophysically realistic simulations of preclinical animal studies. We built a computational model to complement a previously published study of cervical VNS in dogs [40] (Fig 5B). The simulated activation thresholds correspond closely to the *in vivo* thresholds across four fiber types. These data suggest that the therapeutic mechanisms of VNS in large mammals are driven by activation of A- and/or B-fibers (i.e., large and small myelinated fibers, respectively), since the C-fiber (i.e., unmyelinated fiber) activation thresholds are substantially higher than clinical stimulation amplitudes. The accuracy of ASCENT in reproducing this experimental setup demonstrates that it can be used to predict inter-individual variability in fiber response across animals to inform experimental designs. Additionally, a user could leverage ASCENT to design next generation electrodes and stimulation waveforms to activate preferentially fibers of different types or locations in the nerve cross section or to minimize inter-individual variability in fiber response to stimulation.

### 5.4 Predicting nerve responses where experiments are not yet feasible

**Fig 6:**
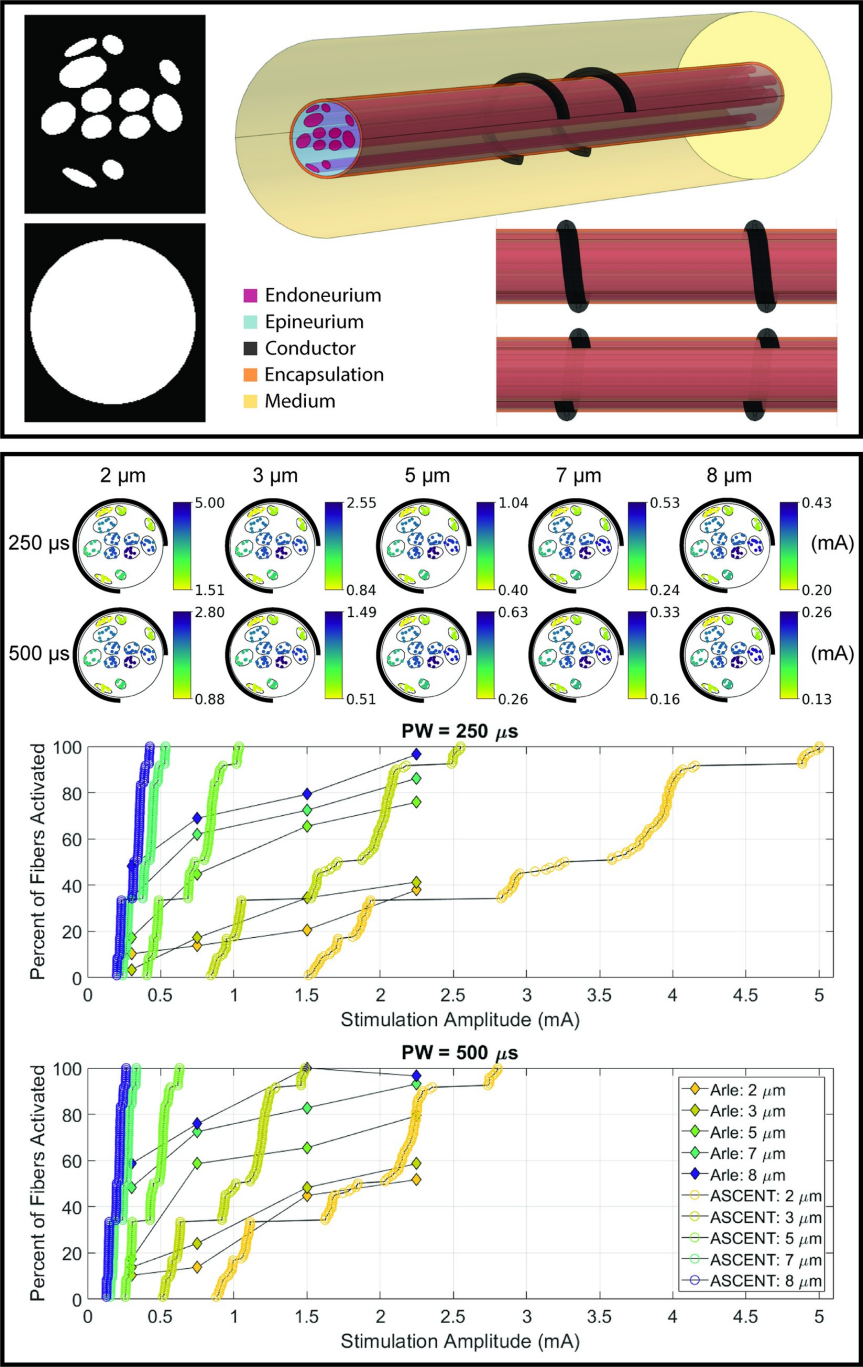
Implementation of a human VNS model as studied in Arle et al. 2016 [39]. Top panel: segmented nerve geometry modeled in the original publication and image of FEM produced with ASCENT. As modeled in Arle et al. 2016 [39], the electrode lacks insulation. Bottom panel: heatmaps and recruitment curves for fiber activation thresholds across fiber diameters and pulse widths. We generated the heatmaps using Query’s heatmaps() method. Consistent with our prior modeling studies [10], the heatmaps show that fibers in the same fascicle with the same diameter have nearly identical thresholds. Configuration and input files used to replicate the results are provided in examples/results/arle_2016/.

ASCENT can predict fiber responses to electrical stimulation where experimental neural recordings are difficult or impossible to obtain. We used ASCENT to simulate activation thresholds of human VNS for the nerve and cuff electrode modeled in Arle et al. 2016 (Fig 6) [39]. ASCENT improves upon Arle’s model implementation in multiple ways. First, their fiber models only contained passive membrane mechanisms, whereas we used biophysically realistic models of myelinated fibers [34] (S21 Text). Second, many of their fibers seemed to be placed outside of the fascicles, in the epineurium; we instead seeded 10 fiber locations in each fascicle (i.e., “UNIFORM_COUNT”). Third, we used more accurate parameter values for material conductivities and perineurium thickness (S27 and S28 Text and Table 1). We obtained activation thresholds over a similar range of amplitudes; however, our data show much steeper recruitment curves, with a smaller amplitude range between onset and saturation thresholds for each fiber diameter.

**Table 1:** Material conductivities used in Arle et al. 2016 [39] and ASCENT.

Material	Arle et al. 2016 [39]	ASCENT
Encapsulation	0.03 [S/m]	0.159 [S/m]
Epineurium	0.054 [S/m]	0.159 [S/m]
Perineurium	0.005668 [S/m]	0.0008703 [S/m]
Endoneurium	{0.08, 0.08, 0.05} [S/m]	{0.167, 0.167, 0.571} [S/m]
Medium	Fat: 0.04 [S/m]	Muscle: {0.086, 0.086, 0.35} [S/m]

## 6 Availability and future directions

The ASCENT pipeline is publicly available for download to any Linux, Mac or Windows machine: the release associated with this paper (v1.0.0), as well as other archived releases, are available through Zenodo (v1.0.0: https://doi.org/10.5281/zenodo.5136631; current release: https://zenodo.org/badge/latestdoi/379064819); the most recent commit—where there may be multiple commits per release—is available through GitHub (https://github.com/wmglab-duke/ascent). The code is documented in a git wiki associated with the code repository (https://github.com/wmglab-duke/ascent/wiki). The project is licensed under the MIT License for non-commercial use (see the LICENSE file in the root of the repository for details).

ASCENT will be used to design experimental and clinical interventions, to interpret experimental results, and as a component of integrated simulations with other models (e.g., organ system models). It will aid in analysis and design of peripheral nerve stimulation therapies including quantification of the effects of intra- and inter-species differences on nerve activation and block, as well as approaches to increase the selectivity and efficiency of activation and block with novel stimulation paradigms and electrode designs.

Future developments of the pipeline will address limitations, including relaxing the assumption of quasi-static conditions for approximating electric fields [16], accounting for the impedance of the electrode-tissue interface [42,43], incorporating variations in nerve cross section that occur along its length, and eliminating dependency on COMSOL. However, the difficulty of building, meshing, and solving such complex geometries should not be under-estimated.

We intend for the ASCENT pipeline to become the standard resource for modeling peripheral nerve stimulation and thereby reduce duplication of efforts, improve accessibility of model-based analysis and design of nerve stimulation, encourage best-practices, and enable reproducibility of simulation data [15,44,45].

## Supporting information

S1 Text Appendix. Metadata required to model an i*n vivo* experiment using the ASCENT pipeline.
(PDF)

S2 Text Appendix. Installation.
(PDF)

S3 Text Appendix. ASCENT data hierarchy.
(PDF)

S4 Text Appendix. Your first run.
(PDF)

S5 Text Appendix. Template for methods reporting.
(PDF)

S6 Text Appendix. Enums.
(PDF)

S7 Text Appendix. JSON configuration files.
(PDF)

S8 Text Appendix. JSON file parameter guide.
(PDF)

S9 Text Appendix. Python utility classes.
(PDF)

S10 Text Appendix. Submitting NEURON jobs.
(PDF)

S11 Text Appendix. Morphology files.
(PDF)

S12 Text Appendix. Python MockSample class for creating binary masks of nerve morphology.
(PDF)

S13 Text Appendix. Python classes for representing nerve morphology (Sample).
(PDF)

S14 Text Appendix. Creating sample-specific nerve morphologies in COMSOL.
(PDF)

S15 Text Appendix. Micro-Leads cuff measurements.
(PDF)

S16 Text Appendix. Library of part primitives for electrode contacts and cuffs.
(PDF)

S17 Text Appendix. Creating custom preset cuffs from instances of part primitives.
(PDF)

S18 Text Appendix. Creating new part primitives.
(PDF)

S19 Text Appendix. Cuff placement on nerve.
(PDF)

S20 Text Appendix. Fiberset.
(PDF)

S21 Text Appendix. Implementation of NEURON fiber models.
(PDF)

S22 Text Appendix. Simulation protocols.
(PDF)

S23 Text Appendix. ModelWrapper class.
(PDF)

S24 Text Appendix. Making geometries in COMSOL (Part class).
(PDF)

S25 Text Appendix. Control of medium surrounding nerve and cuff electrode.
(PDF)

S26 Text Appendix. Java utility classes.
(PDF)

S27 Text Appendix. Defining and assigning materials in COMSOL.
(PDF)

S28 Text Appendix. Definition of perineurium.
(PDF)

S29 Text Appendix. Data interchange between COMSOL and NEURON.
(PDF)

S30 Text Appendix. Python Simulation class.
(PDF)

S31 Text Appendix. NEURON launch.hoc.
(PDF)

S32 Text Appendix. NEURON Wrapper.hoc.
(PDF)

S33 Text Appendix. Data analysis tools.
(PDF)

S34 Text Appendix. Convergence analysis example*.
(PDF)

S35 Text Appendix. Sim4Life validation.
(PDF)

S36 Text Appendix. Comparison of MRG fit to Bucksot et al. 2019.
(PDF)
