# Supplementary material for: ASCENT (Automated Simulations to Characterize Electrical Nerve Thresholds): A pipeline for sample-specific computational modeling of electrical stimulation of peripheral nerves
Source: PLoS Comput Biol. 2021 Sep 7;17(9):e1009285. doi: 10.1371/journal.pcbi.1009285 (PMC8423288; doi:10.1371/journal.pcbi.1009285)
Supplement: S1 Text — Metadata required to model an in vivo experiment using the ASCENT pipeline. (PDF) [file pcbi.1009285.s001.pdf]

1 S1 Text

Appendix. Metadata required to model an *in vivo* experiment using the ASCENT pipeline

1. Detailed specifications / dimensions of the stimulating cuff electrode.
2. Transverse cross section of the nerve where the cuff is placed, stained to visualize the different tissue types (e.g., using Masson's trichrome), with a scale bar (Fig 2 and S11 Text). Different possible sources for defining the nerve sample morphology include:
    a. For best specificity, the nerve would be sampled from the specific animal used in the experiment being modeled. In this case, two colors of tissue dye may be used on the ventral and medial aspects of the nerve to maintain orientation information.
    b. Otherwise, a sample from another animal of the same species could be used at the correct nerve level.
    c. If multiple samples from other animals are available, they could be used to generate a representative nerve model, knowing the range of morphological metrics across individuals using the scripts/mock_morphology_generator.py script (S12 Text).
    d. Lastly, published data could be used.
3. Orientation and rotation of the cuff on the nerve (e.g., cuff closure on the ventral side of the nerve).
4. Fiber diameters
    a. Distributions of fiber diameters may be obtained from literature; otherwise, detailed electromicroscopic studies are required.
    b. The fiber diameters found in the target nerve that will be simulated in NEURON. All diameters or a subset of diameters may be of interest.
    c. Each fiber diameter of interest can be simulated for each fiber location of interest, or specific fiber diameters can be simulated in specific locations.
5. Approximate tissue or fluids surrounding the nerve and cuff (e.g., muscle, fat, or saline).
6. Stimulation waveforms, pulse widths, and other parameters of the electrical signal.
7. If comparing to neural recordings: distance between the stimulation and recording cuffs.
8. If comparing to functional recordings (e.g., EMG): distance from the stimulation cuff to the location where the nerve inserts into the muscle.
