# Supplementary material for: ASCENT (Automated Simulations to Characterize Electrical Nerve Thresholds): A pipeline for sample-specific computational modeling of electrical stimulation of peripheral nerves
Source: PLoS Comput Biol. 2021 Sep 7;17(9):e1009285. doi: 10.1371/journal.pcbi.1009285 (PMC8423288; doi:10.1371/journal.pcbi.1009285)
Supplement: S2 Text — Installation. (PDF) [file pcbi.1009285.s002.pdf]

1 S2 Text

Appendix. Installation

1.1 Installing commercial software

First, these commercial software packages must be manually installed:

- Python 3.7 *(or later; choose 32/64 bit depending on operating system; if not using Miniconda, check "add Python to PATH")*
    - If you already have Python added to your PATH and that installation is version 3.7 or later, there is no need to reinstall Python, and you may use your own preferred Python environment rather than Miniconda. Otherwise, we recommend that you install Miniconda and select the "add to path" option**.**
    - https://www.python.org/downloads/
- Java SE Development Kit 8 (1.8) (need to register for a free account)
    - https://www.oracle.com/java/technologies/javase/javase-jdk8-downloads.html
- COMSOL 5.5 (requires purchase of license; only base package needed, which includes the COMSOL Java API)
    - Once COMSOL 5.5 is installed, alter 'File System Access' permissions via *File → Preferences → Security → Methods and Java Libraries → File System Access → All Files*.
    - https://www.comsol.com/product-download/5.5
- NEURON 7.6 (version 7.7 has since been released, but compatibility has yet to be confirmed; choose appropriate installer depending on operating system; install auxiliary software as prompted by NEURON installer)
    - https://neuron.yale.edu/ftp/neuron/versions/v7.6/
    - If having issues with the NEURON installation, try running the compatibility troubleshooter.

In this stage of development, all programs/commands are run from a command line environment on both MacOS/Linux and Windows operating systems (Bash terminal for MacOS/Linux, Powershell for Windows). For users less familiar with this environment and for the quickest setup, it is suggested that the user install the package management system Miniconda (https://docs.conda.io/en/latest/miniconda.html) (if using MacOS, choose .pkg for ease of use).

If using MacOS to run local NEURON simulations, it may be necessary to install the Xcode Command Line Tools via xcode-select --install, as well as Xquartz (https://www.xquartz.org/releases/XQuartz-2.7.11.html).

Users must also download a text editor or integrated development environment (IDE) of their choosing to view/edit code (e.g., Visual Studio Code (https://code.visualstudio.com/), IntelliJ IDEA

(https://www.jetbrains.com/idea/download/)). The ASCENT installation includes Spyder, so this is only necessary if users wish to use a different program. If users would like to have autocomplete functionality in the IDE of their choice, they must add both the path to the COMSOL installation ending in plugins as well as the path <ASCENT_PROJECT_PATH>/bin/json-20190722.jar to the list of available libraries (usually from within the IDE's project settings).

1.2 Installing ASCENT pipeline

1. First, download or clone the ASCENT pipeline from GitHub (https://github.com/wmglab-duke/ascent) to a desired location that will be referenced in step 3. Downloading is a much simpler process than cloning via Git but does not easily allow for you to get the most recent updates/bug fixes, nor does it allow you to suggest new features/changes. If you are interested in either of these features, you should clone via Git rather than downloading.
    - Downloading:
        1. Click the download button on GitHub (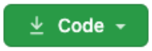

) and choose the location to which you would like to save. Note that you will need to extract the files, as they will be downloaded in a compressed format. When presented with a choice of compression format, ".zip" is a safe choice that most computers will be able to extract.
    - Cloning via Git:
        1. You must first have an account with GitHub, and if you have been granted special permissions to the code repository, you *must* use the email address to which those permissions were granted.
        2. If you have not already done so, add an SSH key to your account (see instructions for GitHub (https://docs.github.com/en/github/authenticating-to-github/connecting-to-github-with-ssh)). This is a required standard authentication method.
        3. In a Miniconda command line environment, navigate to the location to where you would like to clone the code repository (see instructions for navigating the file system from the command line for Mac or Linux (https://www.redhat.com/sysadmin/navigating-linux-filesystem) and Windows (https://blogs.umass.edu/Techbytes/2014/11/14/file-navigation-with-windows-command-prompt/)).
        4. Clone the repository (see instructions for GitHub (https://docs.github.com/en/github/creating-cloning-and-archiving-repositories/cloning-a-repository-from-github)).
        5. For more information on using Git, check out the official documentation (https://git-scm.com/doc).

2. Next, install the Python dependencies:
    - Windows: Open the Miniconda Powershell Prompt from the Windows Start Menu as Administrator and use cd to navigate to the root directory of the pipeline. Then, run "python run install".

- MacOS/Linux: Open a terminal window and use cd to navigate to the root directory of the pipeline. Then, run “python run install”.

After confirming that you are in the correct directory, the script will install the required Python packages: Pillow [1], NumPy [2], Shapely [3], Matplotlib [4], PyClipper [5], pygame [6], QuantiPhy [7], OpenCV [8], PyMunk [9], and SciPy [10], pandas [11], and OpenPyXL [12]. It is crucial that all the packages are installed successfully (check for “successfully installed” for each package). The installation script will also create a shortcut to the newly configured ASCENT Conda “environment” on your project path, which can be used for running the pipeline.

3. Then, configure the environment variables. This step may be completed either manually or automatically (preferred), both of which are described below.
   - Manual setup: Copy the file config/templates/env.json into config/system/env.json (new file). This file holds important paths for software used by the pipeline (see env.json). Then, edit each of the four values as specified below. Use “\\” in Windows and “/” in MacOS/Linux operating systems. Note that the file separators are *operating system dependent*, so even if you installed in step 2 with Unix-like command environment on a Windows machine (e.g., using Git Bash (https://gitforwindows.org/), Cygwin (https://www.cygwin.com/), or a VM with Ubuntu (https://ubuntu.com/)), you will still need to choose the proper file separator for Windows, i.e., “\\”). See example env.json files for both MacOS and Windows (S8 Text).
     - ASCENT_COMSOL_PATH: Path to the COMSOL installation, ending in the directory name “Multiphysics”, as seen in the template.
     - ASCENT_JDK_PATH: Path to the JDK 1.8 installation, ending in “bin”, as seen in the template. Hint: This is the correct path if the directory contains many executables (for Windows: java.exe, etc.; MacOS/Linux: java, etc.).
     - ASCENT_PROJECT_PATH: Path to the root directory of the pipeline, as chosen for step 1.
     - ASCENT_NSIM_EXPORT_PATH: Path to the export location for NEURON “simulation” directories. This path only depends on the user’s desired file system organization.
   - Automatic setup: Upon the initiation of your first run, you will be prompted to enter the above four paths if you did not choose to complete the manual setup. Enter them as prompted, following the guidelines detailed above. Note that you may at any time update paths with “python run env_setup” to rewrite this file if the information should change.
4. The first time you run the pipeline, you must open the COMSOL Server and log in with a username and password of your choosing (arbitrary and not needed thereafter). This can be done by navigating to the bin/ directory in the COMSOL installation and running “comsolmphserver” (Windows) or “./comsol server” (MacOS/Linux).

1.3 References

1. Clark A. Pillow: a modern fork of PIL — Pillow v2.3.0 (PIL fork) [Internet]. 2020 [cited 2020 Apr 20]. Available from: https://pillow.readthedocs.io/en/2.3.0/#
2. Oliphant TE. A Guide to NumPy [Internet]. Trelgol Publishing; 2006. Available from: https://books.google.com/books?id=fKulSgAACAAJ
3. Gillies S. Shapely · PyPI [Internet]. 2019 [cited 2020 Apr 20]. Available from: https://pypi.org/project/Shapely/
4. Hunter JD. Matplotlib: A 2D Graphics Environment. Comput Sci Eng. 2007 May;9(3):90–5.
5. Johnson A, Chalton M, Treyer L, Ratajc G. pyclipper · PyPI [Internet]. 2019 [cited 2020 Apr 20]. Available from: https://pypi.org/project/pyclipper/
6. Shinners P. Pygame Intro — pygame v2.0.0.dev5 documentation [Internet]. [cited 2020 Apr 20]. Available from: https://www.pygame.org/docs/tut/PygameIntro.html
7. Kundert K. QuantiPhy: Physical Quantities — quantiphy 2.10.0 documentation [Internet]. 2020 [cited 2020 Apr 20]. Available from: https://quantiphy.readthedocs.io/en/stable/
8. Bradski G, Daebler A. Learning OpenCV. Computer vision with OpenCV library. 2008 Jan 1;222–64.
9. Blomqvist V. pymunk · PyPI [Internet]. 2019 [cited 2020 Apr 20]. Available from: https://pypi.org/project/pymunk/
10. Virtanen P, Gommers R, Oliphant TE, Haberland M, Reddy T, Cournapeau D, et al. SciPy 1.0: fundamental algorithms for scientific computing in Python. Nat Methods [Internet]. 2020;17(3):261–72. Available from: https://doi.org/10.1038/s41592-019-0686-2
11. The pandas development team. pandas-dev/pandas: Pandas [Internet]. Zenodo; 2020. Available from: https://doi.org/10.5281/zenodo.3509134
12. Gazoni E, Clark C. openpyxl - A Python library to read/write Excel 2010 xlsx/xlsm files [Internet]. 2020. Available from: https://openpyxl.readthedocs.io/en/stable/
