# Supplementary material for: ASCENT (Automated Simulations to Characterize Electrical Nerve Thresholds): A pipeline for sample-specific computational modeling of electrical stimulation of peripheral nerves
Source: PLoS Comput Biol. 2021 Sep 7;17(9):e1009285. doi: 10.1371/journal.pcbi.1009285 (PMC8423288; doi:10.1371/journal.pcbi.1009285)
Supplement: S3 Text — ASCENT data hierarchy. (PDF) [file pcbi.1009285.s003.pdf]

1 S3 Text

Appendix. ASCENT data hierarchy

Each execution of the ASCENT pipeline requires a ***Run*** JavaScript Object Notation (JSON) configuration file (<run_index>.json) that contains indices for a user-defined set of JSON files. Specifically, a JSON file is defined for each hierarchical domain of information: (1) ***Sample***: for processing segmented two-dimensional transverse cross-sectional geometry of a nerve sample, (2) ***Model*** (COMSOL parameters): for defining and solving three-dimensional FEM, including geometry of nerve, cuff, and medium, spatial discretization (i.e., mesh), materials, boundary conditions, and physics, and (3) ***Sim*** (NEURON parameters): for defining fiber models, stimulation waveforms, amplitudes, and durations, intracellular test pulses (for example, when seeking to determine block thresholds), parameters for the binary search protocol and termination criteria for thresholds, and flags to save state variables. These configurations are organized hierarchically such that ***Sample*** does not depend on ***Model*** or ***Sim***, and ***Model*** does not depend on ***Sim***; thus, changes in ***Sim*** do not require changes in ***Model*** or ***Sample***, and changes in ***Model*** do not require changes in ***Sample*** (Figure A).

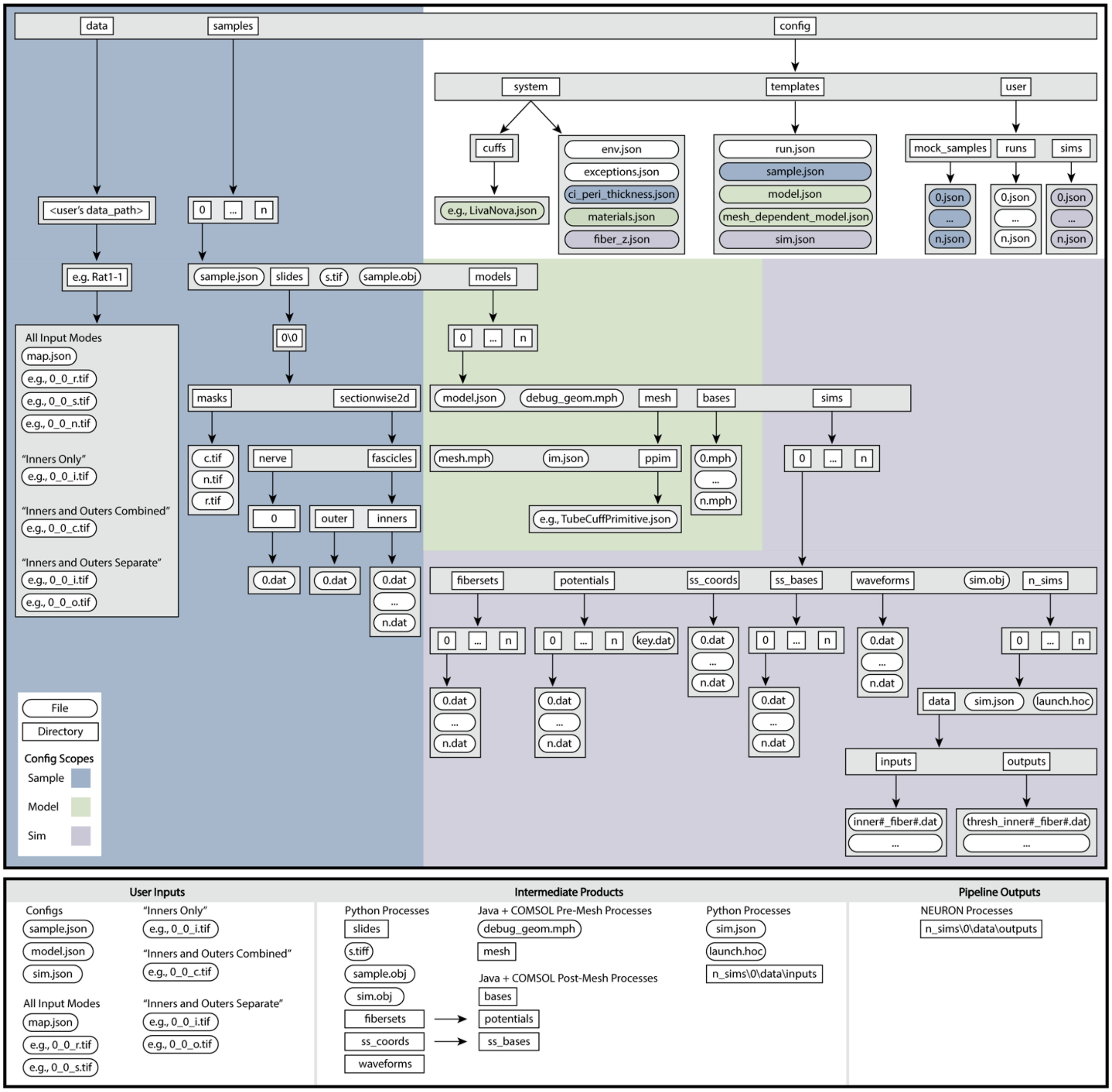

Figure A. ASCENT pipeline file structure in the context of ***Sample*** (blue), ***Model*** (green), and ***Sim*** (purple) configurations. S7 Text describes the JSON configuration files and their contents, and S8 Text details the syntax and data types of the key-value parameter pairs.

1.1 Batching and sweeping of parameters

ASCENT enables the user to batch rapidly simulations to sweep cuff electrode placement on the nerve, material properties, stimulation parameters, and fiber types. The first process of ASCENT prepares ready-to-submit NEURON simulations to model response of fibers to extracellular stimulation. The second process of ASCENT uses Python to batch NEURON jobs to a personal computer or compute cluster to simulate fiber response to extracellular stimulation. Each task

submitted to a CPU simulates the response of a single fiber to either a set of finite amplitudes or a binary search for threshold of activation or block, therefore creating an “embarrassingly parallel” workload.

Groups of fibers from the same ***Sample***, ***Model***, ***Sim***, waveform, contact weight (i.e., “src_weights” in ***Sim***), and fiberset (i.e., a group of fibers with the same geometry and channels and occupy different (x,y)-locations in the nerve cross section) are organized in the same n_sim/ directory.

A ***Run*** creates simulations for a single ***Sample*** and all pairs of listed ***Model(s)*** and ***Sim(s)***. A user can pass a list of ***Run*** configurations in a single system call with “python run pipeline <run_indices>” to simulate multiple ***Sample*** configurations in the same system call.

Sample and ***Model*** cannot take lists of parameters. Rather, if the user would like to assess the impact of ranges of parameters for ***Sample*** or ***Model***, they must create additional ***Sample*** and ***Model*** configuration files for each parameter value.

Sim can contain lists of parameters in “active_srcs” (i.e., cuff electrode contact weightings), “fibers”, “waveform”, and “supersampled_bases”.
