# Supplementary material for: ASCENT (Automated Simulations to Characterize Electrical Nerve Thresholds): A pipeline for sample-specific computational modeling of electrical stimulation of peripheral nerves
Source: PLoS Comput Biol. 2021 Sep 7;17(9):e1009285. doi: 10.1371/journal.pcbi.1009285 (PMC8423288; doi:10.1371/journal.pcbi.1009285)
Supplement: S4 Text — Your first run. (PDF) [file pcbi.1009285.s004.pdf]

1 S4 Text

Appendix. Your first run

How to run the ASCENT pipeline, after completing the initial setup.

The user provides binary mask inputs for the nerve and saves ***Sample*** (i.e., sample.json), ***Model(s)*** (i.e., model.json), and ***Sim(s)*** (i.e., sim.json) JSON configurations in directories, relative to the project path defined in config/system/env.json. The directory names must use indices that are consistent with the indices of ***Sample***, ***Model(s)***, and ***Sim(s)*** defined in ***Run***.

1. **Masks*:*** User populates input/<NAME>/ (e.g., “Rat1-1”, which must match “sample” parameter in ***Sample***) with binary masks of neural tissue boundaries using either:
    a. Segmented histology (S11 Text and Fig 2), or
    b. Running the mock_morphology_generator.py script (S12 Text).
        i. Copy mock_sample.json from config/templates/ to config/user/mock_samples/ as <mock_sample_index>.json and update file contents, including the “NAME” parameter used to construct the destination path for the output binary masks, which serve as inputs to the pipeline.
        ii. Call “python run mock_morphology_generator <mock_sample_index>”.
        iii. The program saves a copy of the user’s mock_sample.json and binary masks in input/<NAME>/.
2. ***For one Sample:*** User copies sample.json from config/templates/ to samples/<sample_index>/ as sample.json and edits its contents to define the processing of binary masks to generate the two-dimensional cross section geometry of the nerve in the FEM. In particular, change “sample” to match <NAME>, the “scale_bar_length” parameter for s.tif (i.e., length in microns of your scale bar, which is oriented horizontally), and “mask_input” in ***Sample*** accordingly (S8 Text). You have now created the directory for your first sample: sample #<sample_index>.
3. ***For each Model:*** User copies model.json from config/templates/ to samples/<sample_index>/models/<model_index>/ as model.json and edits its contents to define the three dimensional FEM.
    a. ***Preset:*** User defines a new “preset” cuff JSON file, which contains instructions for creating their cuff electrode, and saves it as config/system/cuffs/<preset_str>.json.
    b. The <preset_str>.json file name must be assigned to the “preset” parameter in ***Model*** (S8 Text).
4. ***For each Sim:*** User copies sim.json from config/templates/ to config/user/sims/ as <sim_index>.json and edits its contents to inform the NEURON simulations (S8 Text).
5. ***Run:*** User copies run.json from config/templates/ to config/user/runs/ as <run_index>.json and edits the indices for the created ***Sample***, ***Model(s)***, and ***Sim(s)*** configurations (S8 Text).
6. The pipeline is run from the project path (i.e., the path to the root of the ASCENT pipeline, which is defined in config/system/env.json (S8 Text)) with the command “python run

pipeline <run indices>", where <run indices> is a list of space-separated ***Run*** indices (if multiple ***Sample*** indices, one ***Run*** for each). The pipeline outputs ready-to-submit NEURON simulations and associated ***Run*** file(s) to the "ASCENT_NSIM_EXPORT_PATH" directory as defined in config/system/env.json (S8 Text). NEURON simulations are run locally or submitted to a computer cluster with the command "python submit.py <run indices>" from the export directory.

1.1 Task given to beta testers

We sent the pipeline code, manuscript, and the following task to beta testers (both within our lab at Duke and externally). Following this task and verifying the threshold value is a suitable way to familiarize yourself with the ASCENT code and documentation.

We provided segmented histology of a rat cervical vagus nerve (examples/beta_task/). Please simulate activation thresholds in response to a charge balanced, biphasic pulse (PW1 = 100 µs, interphase gap of 100 µs, PW2 = 400 µs) using Purdue's bipolar cuff design.

- MRG 8.7 µm diameter fibers
- Fibers placed in nerve cross section using a 6 spoke wheel with 2 fibers per spoke
- Custom material for surrounding medium with isotropic conductivity 1/20 [S/m]

After your thresholds have been computed, build a heatmap for the threshold at each fiber location using the example script: examples/analysis/heatmap_monofasc.py.

Through this exercise, you will:

- Place and name binary masks of the nerve morphology in the proper directories
    - Binary masks provided
- Define and assign a custom material
- Build and solve a finite element model
- Define placement of fibers in the nerve cross section
- Parameterize your custom stimulation waveform
- Simulate activation thresholds for a specific fiber model by submitting NEURON simulations locally or to a computer cluster
- View and analyze your data
    - Plot samples with color-coded fiber (x,y)-coordinates
    - Heatmap of fiber thresholds

For the following exercise, we ask that you please attempt to accomplish the prescribed modeling tasks with the paper and associated supplemental documentation as your primary reference.

Check: Threshold for inner0_fiber0 (thresh_inner0_fiber0.dat) should be -0.028732 mA

We provided ***Sample***, ***Model***, and ***Sim*** JSON files for the solution in examples/beta_task/.
