# Supplementary material for: ASCENT (Automated Simulations to Characterize Electrical Nerve Thresholds): A pipeline for sample-specific computational modeling of electrical stimulation of peripheral nerves
Source: PLoS Comput Biol. 2021 Sep 7;17(9):e1009285. doi: 10.1371/journal.pcbi.1009285 (PMC8423288; doi:10.1371/journal.pcbi.1009285)
Supplement: S5 Text — Template for methods reporting. (PDF) [file pcbi.1009285.s005.pdf]

1 S5 Text

Appendix. Template for methods reporting

For uses of ASCENT, either stand-alone in its publicly available form, as a starting point upon which further work is developed, or as a component of a system (e.g., connected with other organ models or engineering optimization techniques), we ask that the author(s) cite the ASCENT publication and indicate the release of ASCENT used (e.g., ASCENT v1.0.0).

The following guidelines are to help users adhere to FAIR principles when using ASCENT and thereby enable model reproducibility and reduce duplication of efforts [1]. With published modeling studies using ASCENT, we ask that users provide all code and input files required to reproduce their work.

To streamline dissemination of work, upon acceptance of a manuscript to a journal, we encourage users to either make their fork/branch of the ASCENT repository publicly available, or to make a "Merge Request" to the ASCENT GitHub repository (i.e., if your developments would be of general interest to ASCENT users) so that we can review and incorporate your changes to our public repository.

1.1 Configuration files

Provide the JSON configuration files used as inputs to ASCENT.

- sample.json
- model.json
- sim.json
- mock_sample.json (if applicable)
- map.json (if serial nerve sections are used to create 3D representation of the nerve in future ASCENT releases)
- ci_peri_thickness.json (if using new relationships between perineurium thickness and inner diameter not already in the ASCENT repository)
- materials.json (if using new materials that have been added to this file and referenced in model.json rather than explicitly defined in model.json)
- fiber_z.json (if using fiber models with ultrastructure that is not integrated in the ASCENT repository)

1.2 Nerve segmentation files

Indicate the software and methods used to generate the binary image inputs (e.g., Adobe Photoshop).

If defining nerve geometry with segmented binary images of nerve microanatomy, indicate the methods for the technique used to obtain the nerve images (i.e., histology, Micro-CT, ultrasound, etc.) and to segment the tissue boundaries.

If referencing a dataset of published nerve microanatomy, cite the original source, include statements to justify the relevance of the source to define nerve tissue boundaries for your model(s), and report high-level statements for the methods used. Indicate the methods used to segment the tissue boundaries.

If using our mock_morphology_generator.py script, please indicate so, and make a statement regarding the methods used to populate the nerve with fascicles. Present either your own data or cite previously published literature used to inform the nerve diameter and the number, size, and placement of fascicle(s) in the nerve.

Explicitly indicate in your methods if you used any correction for tissue shrinkage in preparing nerve inputs for modeling in the FEM. If so, cite literature justifying your assumption. Additionally, indicate any methods used to deform the nerve to fit within the cuff electrode in ASCENT.

Provide the image files used as inputs to ASCENT.

- n.tif (if applicable)
- i.tif or o.tif, both i.tif and o.tif, or c.tif (as applicable)
- s.tif
- a.tif (if applicable)

1.3 Cuff

If the “preset” cuff configuration file used to define the cuff electrode is not publicly available in the ASCENT repository, please provide it with your materials.

If new part primitives were created to represent the cuff electrode (S18 Text), please include copies of Java code that perform the FEM operations for the new parts. These sections of code should be from src/model/Part.java as “cases” (i.e., in switch-case statement) for new parts in the createCuffPartPrimitive() and createCuffPartInstance() methods.

Indicate how the cuff was placed on the nerve. Specifically, state the longitudinal placement of the cuff and how the cuff rotation was determined (e.g., the cuff rotation modes, or used a.tif to rotate the cuff to replicate in vivo cuff rotation).

1.4 Materials

Cite the original source for each conductivity value used to define materials as indicated in materials.json and/or model.json.

1.5 Domains

Report the model length and radius, along with a statement to justify your model's dimensions (i.e., convergence studies) (S34 Text). Report the presence and dimensions of any cuff fill domains (e.g., saline, encapsulation, or mineral oil between the nerve and the cuff electrode).

1.6 Perineurium

Report if a thin-layer approximation or a finite thickness material was used to define the perineurium. If a thin-layer approximation was used, indicate how the thickness of the perineurium was determined (e.g., measured from histology, or previously published relationship between inner diameter and perineurium thickness).

1.7 Mesh

Indicate the method used to mesh the FEM in addition to the number of domain elements. Include a statement to justify your model's meshing parameters (i.e., convergence studies) (S34 Text).

1.8 Solution

Indicate that the FEM was solved using Laplace's equation once for each contact delivering 1 mA of current. Indicate if the outer surfaces of the model were grounded or set to insulation.

1.9 HOC/MOD files

If using novel fiber model ultrastructure or channel mechanisms, please share the code required to implement it in ASCENT.

1.10 Waveform

State the stimulation waveform shape, timestep, and simulation time used. Be certain that the timestep chosen is short enough to simulate accurately fiber response to stimulation.

1.11 NEURON simulations

If computing thresholds of activation and/or block, state the search algorithm used (e.g., binary search) and the exit criteria (e.g., tolerance).

1.12 Analysis

State the methods and software tools used to analyze and present data.

1.13 References

1. Wilkinson MD, Dumontier M, Aalbersberg IJJ, Appleton G, Axton M, Baak A, et al. The FAIR Guiding Principles for scientific data management and stewardship. Sci data. 2016 Mar;3:160018. Available from: https://doi.org/10.1038/sdata.2016.18 PMID: 26978244
