# Supplementary material for: ASCENT (Automated Simulations to Characterize Electrical Nerve Thresholds): A pipeline for sample-specific computational modeling of electrical stimulation of peripheral nerves
Source: PLoS Comput Biol. 2021 Sep 7;17(9):e1009285. doi: 10.1371/journal.pcbi.1009285 (PMC8423288; doi:10.1371/journal.pcbi.1009285)
Supplement: S6 Text — Enums. (PDF) [file pcbi.1009285.s006.pdf]

1 S6 Text

Appendix. Enums

In the Python portions of the pipeline we use Enums (https://docs.python.org/3/library/enum.html) which are "… a set of symbolic names (members) bound to unique, constant values. Within an enumeration, the members can be compared by identity, and the enumeration itself can be iterated over." Enums improve code readability and are useful when a parameter can only assume one value from a set of possible values.

We store our Enums in src/utils/enums.py. While programming in Python, Enums are used to make interfacing with our JSON parameter input and storage files easier. We recommend that as users expand upon ASCENT's functionality that they continue to use Enums, adding to existing classes or creating new classes when appropriate.
