# Supplementary material for: ASCENT (Automated Simulations to Characterize Electrical Nerve Thresholds): A pipeline for sample-specific computational modeling of electrical stimulation of peripheral nerves
Source: PLoS Comput Biol. 2021 Sep 7;17(9):e1009285. doi: 10.1371/journal.pcbi.1009285 (PMC8423288; doi:10.1371/journal.pcbi.1009285)
Supplement: S7 Text — JSON configuration files. (PDF) [file pcbi.1009285.s007.pdf]

1 S7 Text

Appendix. JSON configuration files

We store parameters in JSON configuration files because the JSON format is accessible, readable, and well-documented for metadata interchange. The configuration files inform the pipeline in its operations and provide a traceable history of the parameters used to generate data.

For each JSON file, we provide a brief overview, a statement of where the file must be placed in the directory structure, and a description of its contents. For a detailed description of each JSON file (i.e., which parameters are required or optional, known value data types, and known values), see S8 Text. Though JSON does not allow comments, users may want to add notes to a JSON file (e.g., to remember what a ***Sample***, ***Model***, or ***Sim*** file was used to accomplish). The user can simply add a key to the JSON file that is not in use (e.g., “notes”) and provide a value (String) with a message.

1.1 User configuration files

1.1.1 run.json

The run.json file is passed to *pipeline.py* to instruct the program which ***Sample***, ***Model(s)***, and ***Sim(s)*** to run. All run.json files are stored in the config/user/runs/ directory. Since each ***Sample***, ***Model***, and ***Sim*** is indexed, their indices are used as the identifying values in run.json. Additionally, the file contains break points that enable the user to terminate the pipeline at intermediate processes, flags for user control over which COMSOL files are saved, flags to selectively build a “debug_geom.mph” file of just the nerve or cuff electrode, and flags to recycle previous meshes where appropriate. Lastly, the run.json file reports which ***Model*** indices were generated successfully in COMSOL and indicates if the user is submitting the NEURON jobs to a SLURM cluster or locally.

1.1.2 sample.json

An example ***Sample*** configuration file is stored in config/templates/ for users to reference when defining their own input nerve morphology from histology or from the mock nerve morphology generator (S8 and S12 Text). A user’s sample.json file is saved in the samples/<sample_index>/ directory. The file contains information about the sample’s properties and how to process the nerve morphology before placing the nerve in a cuff electrode. The pipeline’s processes informed by ***Sample*** output the Python object sample.obj.

The information in ***Sample*** must parallel how the sample morphology binary images are saved on the user’s machine (sample name, path to the sample). The user must define the “mask_input” parameter in ***Sample*** to indicate which set of input masks will be used. ***Sample*** also contains

parameters that determine how the morphology is processed before being represented in the FEM such as shrinkage correction (“shrinkage”), required minimum fascicle separation (“boundary_separation”), presence of epineurium (“nerve”), perineurium thickness (“ci_perineurium_thickness”), deformation method (“deform”), reshaped nerve profile (“reshape_nerve”), and parameters for specifying CAD geometry input file formats for nerve morphology (“write”). ***Sample*** also contains information about the binary image of the scale bar (i.e., “scale_bar_length”).

The value of the “ci_perineurium_thickness” in ***Sample*** refers to a JSON Object in config/system/ci_peri_thickness.json that contains coefficients for linear relationships between inner diameter and perineurium thickness (i.e., $thk_{peri,inner}$ = a*($diameter_{inner}$) + b). In ci_peri_thickness.json, we provided a “PerineuriumThicknessMode” named “GRINBERG_2008”, which defines perineurium thickness as 3% of inner diameter [1], and relationships for human, pig, and rat vagus nerve perineurium thickness (i.e., “HUMAN_VN_INHOUSE_200601”, “PIG_VN_INHOUSE_200523”, and “RAT_VN_INHOUSE_200601”) [2]. As additional vagus nerve morphometry data become available, users may define perineurium thickness with new models by adding the coefficients to this JSON file.

1.1.3 mock_sample.json

The mock_sample.json file, which is stored in the file structure in config/user/mock_samples/<mock_sample_index>/, is used to define binary segmented images that serve as inputs to the pipeline. In the “populate” JSON Object, the user must define the “PopulateMode” (e.g., EXPLICIT, TRUNCNORM, UNIFORM defined by the “mode” parameter), which defines the process by which the nerve morphology is defined in the MockSample Python class. Each “PopulateMode” requires a certain set of parameters to define the nerve and to define and place the fascicles; the set of parameters for each “PopulateMode” are defined in config/templates/mock_sample_params_all_modes.json.

Probabilistic “PopulateModes” (i.e., TRUNCNORM, UNIFORM) populate an elliptical nerve with elliptical fascicles of diameter and eccentricity defined by a statistical distribution. Since the nerve morphology parameters are defined probabilistically, a “seed” parameter is required for the random number generator to enable reproducibility. The fascicles are placed at randomly chosen locations within the nerve using a disk point picking method; the fascicles are placed at a rotational orientation randomly chosen from 0-360°. If a fascicle is placed in the nerve without maintaining a user-defined “min_fascicle_separation” distance from the other fascicles and the nerve, another randomly chosen point within the nerve is chosen until either a location that preserves a minimum separation is achieved or the program exceeds a maximum number of attempts (“max_attempt_iter”).

The EXPLICIT “PopulateMode” populates an elliptical nerve with elliptical fascicles of user-defined sizes, locations, and rotations. The program validates that the defined fascicle ellipses are at least “min_fascicle_separation” distance apart; otherwise, if the conditions are not met, the program throws an error.

1.1.4 model.json

An example ***Model*** configuration file is stored in config/templates/ for users to reference when creating their own FEMs. As such, model.json, which is stored in the file structure in samples/<sample_index>/models/<model_index>/, contains information to define an FEM uniquely. ***Model*** defines the cuff electrode geometry and positioning, the simulated environment (e.g., surrounding medium dimensions, material properties (including temperature and frequency factors of material conductivity), and physics), the meshing parameters (i.e., how the volume is discretized), and output statistics (e.g., time required to mesh, mesh element quality measures).

1.1.5 sim.json

An example ***Sim*** configuration file is stored in config/templates/ for users to reference when creating their own simulations of fiber responses to stimulation for a sample in a FEM. All simulation configuration files are stored in the config/user/sims/ directory. ***Sim*** defines fiber types, fiber locations in the FEM, fiber length, extracellular (e.g., pulse repetition frequency) and intracellular stimulation, and input parameters to NEURON (e.g., parameters to be saved in the output, binary search algorithm bounds and resolution). Since users may want to sweep parameters at the ***Sim*** configuration level (e.g., fiber types, fiber locations, waveforms), a pared down copy of ***Sim*** that contains a single value for each parameter (rather than a list) is saved within the corresponding n_sims/ directory (S3 Text). These pared down files are provided for convenience, so that the user can inspect which parameters were used in a single NEURON simulation, and they do not hold any other function within the pipeline.

1.1.6 query_criteria.json

In data analysis, summary, and plotting, the user needs to inform the program which output data are of interest. The query_criteria.json file stores the “criteria” for a user’s search through previously processed data. The query_criteria.json file may be used to guide the Query class’s searching algorithm in the run() method. We suggest that all query_criteria.json-like files are stored in the config/user/query_criteria/ directory; however, the location of these files is arbitrary, and when initializing the Query object, the user must manually pass in the path of either the query_criteria.json-like file or the hard-coded criteria as a Python dictionary. An instance of the Query class contains the “criteria” and an empty _result, which is populated by Query’s run() method with found indices of ***Sample***, ***Model***, and ***Sim*** that match the criteria given.

Query’s run() method loops through all provided indices (i.e., ***Sample***, ***Model***, ***Sim***) in the query criteria, and calls _match() when a possible match is found. Note that the presence of an underscore in the _match() method name indicates that it is for internal use only (not to be called by external files). The _match() method compares its two inputs, (1) query_criteria.json and (2) either ***Sample*** (i.e., sample.json), ***Model*** (i.e., model.json), or ***Sim*** (sim.json); the two JSON files are loaded into memory as Python dictionaries. The method returns a Boolean indicating if the input configuration file satisfies the restricted parameter values defined in query_criteria.json. The user may explicitly specify the indices of the ***Sample***, ***Model***, and ***Sim*** configuration files of interest simultaneously with restricted criteria for specific parameter values. The indices added

will be returned in addition to matches found from the search criteria in the ***Sample***, ***Model***, and ***Sim*** criteria JSON Objects.

The query_criteria.json file contains JSON Objects for each of the ***Sample***, ***Model***, and ***Sim*** configuration files. Within each JSON Object, parameter keys can be added with a desired value that must be matched in a query result. If the parameter of interest is found nested within a JSON Object structure or list in the configuration file, the same hierarchy must be replicated in the query_criteria.json file.

The query_criteria.json parameter "partial_matches" is a Boolean indicating whether the search should return indices of ***Sample***, ***Model***, and ***Sim*** configuration files that are a partial match, i.e., the parameters in query_criteria.json are satisfied by a subset of parameters listed in the found JSON configuration.

The query_criteria.json parameter "include_downstream" is a Boolean indicating whether the search should return indices of downstream (***Sample***>***Model***>***Sim***) configurations that exist if match criteria are not provided for them. For example, if only criteria for a ***Sample*** and ***Model*** are provided, Query will return the indices of ***Sample*** and ***Model*** that match the criteria. In addition, the indices of the ***Sims*** downstream of the matches are included in the result if "include_downstream" is true (since the user did not specify criteria for ***Sim***). Otherwise, if "include_downstream" is false, no ***Sim*** indices are returned.

1.2 config/system/

1.2.1 env.json

The env.json file stores the file paths for:

- COMSOL
- Java JDK
- The project path (i.e., the path to the root of the ASCENT pipeline)
- Destination directory for NEURON simulations to run (this could be the directory from which the user calls NEURON, or an intermediate directory from which the user will move the files to a compute cluster)

When the pipeline is run, the key-value pairs are stored as environment variables so that they are globally accessible.

1.2.2 exceptions.json

The exceptions.json file contains a list of exceptions that are intentionally thrown in the Python portion of the pipeline. Each error has its own "code" (index), "text" (informative message hinting to the reason the program failed), and a "source" (indicating in which Python class and method the error occurred). As developers add new methods to Python classes that inherit the Exceptionable class, appending errors onto exceptions.json that are called from Python code file (i.e., self.throw(<exception index>)) will help give informative feedback to the user.

1.2.3 materials.json

The materials.json file contains default values for material properties that can be assigned to each type of neural tissue, each electrode material, the extraneural medium, and the medium between the nerve and inner cuff surface. The materials are referenced by using their labels in the "conductivities" JSON Object of ***Model***.

1.2.4 fiber_z.json

The fiber_z.json file defines z-coordinates to be sampled along the length of the FEM for different fiber types to be simulated in NEURON. In some instances, the section lengths are a single fixed value. In other instances, such as the MRG model [3], the section lengths are defined for each fiber diameter in a discrete list. Section lengths can also be a continuous function of a parameter, such as fiber diameter, defined as a mathematical relationship in the form of a string to be evaluated in Python. Additionally, the file contains instructions (e.g., flags) that corresponds to fiber-type specific operations in NEURON.

1.2.5 ci_perineurium_thickness.json

In the case of fascicles with exactly one inner perineurium trace for each outer perineurium trace, to reduce the required computational resources, the pipeline can represent the perineurium using a thin layer approximation in COMSOL (S28 Text). Specifically, if ***Model's*** "use_ci" parameter is true, the perineurium is modeled as a surface with a sheet resistance (termed "contact impedance" in COMSOL) defined by the product of the resistivity and thickness. The thickness is calculated as half of the difference between the effective circular diameters of the outer and inner perineurium traces. If each fascicle is only defined by a single trace (rather than inner and outer perineurium traces), the user chooses from a list of modes in ***Sample*** for assigning a perineurium thickness (e.g., 3% of fascicle diameter [1], "ci_perineurium_thickness" parameter in ***Sample***).

1.2.6 mesh_dependent_model.json

Since meshing can take substantial time and RAM, if the FEM has the same geometry and mesh parameters as a previous model, this JSON file allows the mesh to be reused if the mesh.mph file is saved. In mesh_dependent_model.json, the keys match those found in ***Model***, however, instead of containing parameter values, each key's value is a Boolean indicating true if the parameter value must match between two ***Model*** configurations to recycle a mesh, or false if a different parameter value would not prohibit a mesh be reused. The mesh_dependent_model.json file is used by our ModelSearcher Java utility class (S26 Text).

1.3 References

1. Grinberg Y, Schiefer MA, Tyler DJ, Gustafson KJ. Fascicular perineurium thickness, size, and position affect model predictions of neural excitation. IEEE Trans Neural Syst Rehabil Eng. 2008 Dec;16(6):572–81. Available from: https://doi.org/10.1109/tnsre.2008.2010348 PMID: 19144589
2. Pelot NA, Goldhagen GB, Cariello JE, Musselman ED, Clissold KA, Ezzell JA, et al.

Quantified Morphology of the Cervical and Subdiaphragmatic Vagus Nerves of Human, Pig, and Rat. Front Neurosci [Internet]. 2020;14:1148. Available from: https://doi.org/10.3389/fnins/2020.601479 PMID: 33250710
3. McIntyre CC, Richardson AG, Grill WM. Modeling the excitability of mammalian nerve fibers: influence of afterpotentials on the recovery cycle. J Neurophysiol. 2002 Feb;87(2):995–1006. Available from: https://doi.org/10.1152/jn.00353.2001 PMID: 11826063
