# Supplementary material for: ASCENT (Automated Simulations to Characterize Electrical Nerve Thresholds): A pipeline for sample-specific computational modeling of electrical stimulation of peripheral nerves
Source: PLoS Comput Biol. 2021 Sep 7;17(9):e1009285. doi: 10.1371/journal.pcbi.1009285 (PMC8423288; doi:10.1371/journal.pcbi.1009285)
Supplement: S8 Text — JSON file parameter guide. (PDF) [file pcbi.1009285.s008.pdf]

1 S8 Text

Appendix. JSON file parameter guide

Notes:

See S7 Text for a general overview of the contents and usage of each of the following JSON files used in ASCENT.

“//" is not valid JSON syntax; comments are not possible in JSON. However, we sparingly used this notation in the JSON examples below to provide context or more information about the associated line. Each value following a key in the syntax denotes the *type* of the value, not its literal value: “[<Type X>, …]” syntax indicates that the type is an array of Type X. Occasionally, a single value may be substituted for the list if only a single value is desired, but this functionality differs between keys, so be sure to read the documentation before attempting for any given key-value pair. If a parameter is optional, the entire key-value can be omitted from the JSON file and the default value will be used.

For calculated values, the user can either keep the key with a dummy value or remove the key entirely when setting up JSON files for a pipeline run.

JSON Object names, keys, and values (e.g., filename strings) are case-sensitive.

The order of key-value pairs within a JSON Object does not matter. Saving/loading the file to/from memory is likely to reorder the contents of the file.

1.1 run.json

1. Named file: config/user/runs/<run_index>.json
2. Purpose: Instructs the pipeline on which input data and user-defined parameters to use in a single program “run”, where one “run” configuration serves a single ***Sample*** and a list of ***Model(s)*** and ***Sims(s)***. Enables operational control (breakpoints, which FEM files to save/discard). Keeps track of successful/failed FEMs in Java.
3. Syntax: To declare this entity in config/user/runs/, use the following syntax:

```
{
 "submission_context": String,
 "sample": Integer, // note, only one value here!
 "models": [Integer, ...], // pipeline will create all pairwise combos of …
 "sims": [Integer, ...],   // … models and sims
 "recycle_meshes": Boolean,
 "break_points": {
```

```
   "pre_java": Boolean, // before Runner's handoff() method to Java/COMSOL
   "pre_geom_run": Boolean, // immediately before geometry operations
   "post_geom_run": Boolean, // immediately after geometry operations
   "pre_mesh_proximal": Boolean, // immediately before mesh prox operations
   "post_mesh_proximal": Boolean, // immediately post mesh prox operations
   "pre_mesh_distal": Boolean, // immediately before mesh dist operations
   "post_mesh_distal": Boolean, // immediately post mesh dist operations
   "post_material_assign": Boolean, // immediately post assigning materials
   "pre_loop_currents": Boolean // immediately before solving for bases
 },
 "models_exit_status": [Boolean, ...], // one entry for each Model
 "keep": {
   "debug_geom": Boolean,
   "mesh": Boolean,
   "bases": Boolean
 },
 "partial_fem": {
   "cuff_only": Boolean,
   "nerve_only": Boolean
 },
 "local_avail_cpus": Integer,
 "override_compiled_mods": Boolean
}
```

4. Properties:

"submission_context": The value (String) of this property tells the system how to submit the n_sim NEURON jobs based on the computational resources available. Value can be either "cluster" or "local". Required.

"sample": The value (Integer) of this property sets the sample configuration index ("***Sample***"). Note that this is only ever one value. To loop ***Samples***, create a ***Run*** for each. Required.

"models": The value ([Integer, ...]) of this property sets the model configuration indices ("***Model***"). Required.

"sims": The value ([Integer, ...]) of this property sets the simulation configuration indices ("***Sim***"). Required.

"recycle_meshes": The value (Boolean) of this property instructs the pipeline to search for mesh matches for recycling a previously generated FEM mesh if set to true. If this property is not specified, the default behavior of the pipeline is false, meaning that it will not search for and

recycle a mesh match (see ModelSearcher (S26 Text) and mesh_dependent_model.json (S7 Text)). Optional.

“break_points”: The value (Boolean) of each breakpoint results in the program terminating or continuing with the next ***Model*** index. In Runner, the program checks that at most one breakpoint is true and throws an exception otherwise. The breakpoint locations enable the user to run only up to certain steps of the pipeline, which can be particularly useful for debugging. If a breakpoint is not defined, the default behavior is false (i.e., the pipeline continues beyond the breakpoint). Optional.

“models_exit_status”: The value ([Boolean, ...]) of this property indicates if Java successfully made the FEMs for the corresponding model indices (“models” property). The user does not need to include this property before performing a run of the pipeline, as it is automatically added in Java (COMSOL FEM processes) and is then used to inform Python operations for making NEURON simulations. The value will contain one value for each ***Model*** listed in “models”. If a ***Model*** fails, the pipeline will skip it and proceed to the next one. Automatically added.

“keep”: The value (Boolean) of each property results in the program keeping or deleting large COMSOL *.mph files for the “debug_geom.mph”, “mesh.mph” and bases/ for a given ***Model***. If a keep property is not defined, the default behavior is true and the associated *.mph file is saved. If “mesh.mph” is saved, the file can later be used if another ***Model*** is a suitable “mesh match” and “recycle_meshes” is true (see ModelSearcher (S26 Text) and mesh_dependent_model.json (S7 Text)). If bases/ are saved, a new ***Sim*** for a previously computed ***Sample*** and ***Model*** can be probed along new fibersets/ to create potentials/. Optional.

“partial_fem”: The value (Boolean) of each property results in the program terminating after building the COMSOL FEM geometry for only the cuff (“cuff_only”) or only the nerve (“nerve_only”). The program terminates after the “debug_geom.mph” file is created. If the “partial_fem” JSON Object is not included, the value of each Boolean is treated as false, meaning that the “debug_geom.mph” file will contain the nerve and cuff electrode. An error is thrown if the user may set both values for “cuff_only” and “nerve_only” to true. To build the geometry of both the cuff and the nerve, but not proceed with meshing or solving the FEM, the user should set the value for “post_geom_run” under “break_points” to true. Optional.

“local_avail_cpus”: The value (Integer) sets the number of CPUs that the program will take if the “submission_context” is “local”. We check that the user is not asking for more than one less that the number of CPUs of their machine, such that at least one CPU is left for the machine to perform other processes. Optional, but if using submitting locally, the program will take all CPUs except 1 if this value is not defined.

“override_compiled_mods”: The value (Boolean) indicates if the program will override previously compiled *.mod files (i.e., files defining channel mechanisms in NEURON) with each system call of submit.py. Optional, but if the key is omitted the program will not override previously compiled *.mod files.

5. Example:


```
{
 "submission_context": “cluster”,
 "sample": 62,
 "models": [0],
 "sims": [99],
 "recycle_meshes": true,
 "break_points": {
   "pre_java": false,
   "pre_geom_run": false,
   "post_geom_run": true,
   "pre_mesh_proximal": false,
   "post_mesh_proximal": false,
   "pre_mesh_distal": false,
   "post_mesh_distal": false,
   "post_material_assign": false,
   "pre_loop_currents": false
 },
 "models_exit_status": [true],
 "keep": {
   "debug_geom": true,
   "mesh": true,
   "bases": true
 },
 "partial_fem": {
   "cuff_only": false,
   "nerve_only": false
 },
 "local_avail_cpus": 3,
 "override_compiled_mods": false
}
```


1.2 sample.json

1. Named file: samples/<sample_index>/sample.json
2. Purpose: Instructs the pipeline on which sample-specific input data and user-defined parameters to use for processing nerve sample morphology inputs in preparation for 3D representation of the sample in the FEM (***Sample***) (S7 and S14 Text).
3. Syntax: To declare this entity in samples/<sample_index>/sample.json, use the following syntax:

```
{
 "sample": String,
 "sex": String,
 "level": String,
 "samples_path": [
   String
 ],
 "scale": {
   "scale_bar_length": Double,
   "shrinkage": Double
 },
 "boundary_separation": {
   "fascicles": Double,
   "nerve": Double
 },
 "modes": {
   "mask_input": String,
   "nerve": String,
   "deform": String,
   "ci_perineurium_thickness": String,
   "reshape_nerve": String
 },
 "morph_count": Integer,
 "deform_ratio": Double,
 "Morphology": {
   "Nerve": {
       "Area": Double
   },
   "Fascicles": [
     {
       "outer": {
         "area": Double,
         "x": Double,
         "y": Double,
         "a": Double,
         "b": Double,
         "angle": Double
       },
       "inners": [
         {
           "area": Double,
           "x": Double,
           "y": Double,
           "a": Double,
```

```
          "b": Double,
          "angle": Double
        },
        ...
      ]
    },
    ...
  ]
 }
}
```

4. Properties:

“sample”: The value (String) of this property sets the sample name/identifier (e.g., “Rat1-1”) to relate to bookkeeping for input morphology files (S3 Text Figure A). The value must match the directory name in input/<NAME>/ that contains the input morphology files. Required.

“sex”: The value (String) of this property assigns the sex of the sample. Optional, for user records only.

“level”: The value (String) of this property assigns the location of the nerve sample (e.g., cervical, abdominal, pudendal). Optional, for user records only.

“samples_path”: The value ([String, ...]) is the path to the directory containing input morphology files for all samples for a given project. Required.

“scale”

- “scale_bar_length”: The value (Double, units: micrometer) is the length of the scale bar in the binary image of the scale bar provided (s.tif, note that the scale bar must be oriented horizontally). Required.
- “shrinkage”: The value (Double) is the shrinkage correction for the nerve morphology binary images provided as a decimal (e.g., 0.20 results in a 20% expansion of the nerve, and 0 results in no shrinkage correction of the nerve). Required, must be greater than 0.

“boundary_separation”

- “fascicles”: The value (Double, units: micrometer) is the minimum distance required between boundaries of adjacent fascicles (post-deformation, see Deformable in S13 Text). Required for samples with multiple fascicles.
  - Note that this is a distinct parameter from “min_fascicle_separation” in mock_sample.json, which controls the minimum distance between fascicles in the binary image mask inputs to the pipeline, which is later deformed to fit in the cuff electrode using Deformable (S13 Text).

- “nerve”: The value (Double, units: micrometer) is the minimum distance required between the boundaries of a fascicle and the nerve (post-deformation, see Deformable in S13 Text). Required if “nerve” in ***Sample*** is “PRESENT”.

“modes”:

- “mask_input”: The value (String) is the “MaskInputMode” that tells the program which segmented histology images to expect as inputs for fascicles. Required.
    - As listed in Enums (S6 Text), modes include:
        1. “INNERS”: Program expects segmented images of only inner fascicle boundaries.
        2. “INNER_AND_OUTER_SEPARATE”: Program expects segmented image of inners in one file and segmented image of outers in another file.
        3. “INNER_AND_OUTER_COMPILED”: Program expects a single segmented image containing boundaries of both inners and outers.
- “nerve”: The value (String) is the “NerveMode” that tells the program if there is an outer nerve boundary (epineurium) segmented image to incorporate into the model. Required.
    - As listed in Enums (S6 Text), known modes include:
        1. “PRESENT”: Program expects a segmented image for a nerve (n.tif) to incorporate into the model. The value must be PRESENT if multifascicular nerve, but can also be PRESENT if monofascicular.
        2. “NOT_PRESENT”: Program does not try to incorporate a nerve boundary into the model. The value cannot be NOT_PRESENT if multifascicular nerve, but can be NOT_PRESENT if monofascicular.
- “deform”: The value (String) is the “DeformationMode” that tells the program which method to use to deform the nerve within the cuff. If the “NerveMode” (i.e., “nerve” parameter) is defined as “NOT_PRESENT” then the “DeformationMode” (i.e., “deform” parameter) must be “NONE”. Required.
    - As listed in Enums (S6 Text), known modes include:
        1. “NONE”: The program does not deform the nerve when it is placed in the cuff electrode. In the pipeline’s current implementation, this should be the value for all nerve samples without epineurium (i.e., “NOT_PRESENT” for “nerve”).
        2. “PHYSICS”: The program uses a physics-based deformation of the nerve to the final inner profile of the nerve cuff, morphing from the original trace towards a circular trace. In the pipeline’s current implementation, this is the required “DeformationMode” for modeling compound nerve samples. See “deform_ratio” below; if deform_ratio = 0, then the original nerve trace is used and if deform_ratio = 1, then the nerve trace will be made circular.
- “ci_perineurium_thickness”: The value (String) is the “PerineuriumThicknessMode” that tells the program which method to use to define perineurium thickness. Required.
    - As listed in Enums (S6 Text), known “PerineuriumThicknessModes” include:
        - “MEASURED”: The program determines the average thickness of the perineurium using the provided inner and outer boundaries (i.e.,

        “MaskInputMode”: INNER_AND_OUTER_SEPARATE or INNER_AND_OUTER_COMPILED). The thickness is determined by finding the half the difference in diameters of the circles with the same areas as the inner and outer traces of the fascicle.
    - Linear relationships between fascicle diameter and perineurium thickness as stored in config/system/ci_peri_thickness.json:
        - “GRINBERG_2008”
        - “PIG_VN_INHOUSE_200523”
        - “RAT_VN_INHOUSE_200601”
        - “HUMAN_VN_INHOUSE_200601”
- “reshape_nerve”: The value (String) is the “ReshapeNerveMode” that tells the program which final nerve profile (post-deformation) to use when “deform” is set to “PHYSICS”. Required.
    - As listed in Enums (S6 Text), known “ReshapeNerveModes” include:
        - “CIRCLE”: The program creates a circular nerve boundary with a preserved cross-sectional area (i.e., for multifascicular nerves/nerves that have epineurium).
        - “NONE”: The program does not deform the nerve boundary (i.e., for monofascicular nerves/nerves that do not have epineurium).

“morph_count”: The value (Integer) can be used to set the number of intermediately deformed nerve traces between the boundary_start and boundary_end in Deformable (S13 Text) if the “DeformationMode” is “PHYSCIS”. An excessively large number for morph_count will slow down the deformation process, though a number that is too small could result in fascicles “escaping” from the nerve. Optional; if not specified default value is 36.

“deform_ratio”: The value (Double, range: 0 to 1) can be used to deform a nerve if “DeformationMode” is “PHYSICS” to an intermediately deformed boundary_end. For example, if the “ReshapeNerveMode” is “CIRCLE” and deform_ratio = 1, then the boundary_end will be a circle. However, if the “ReshapeNerveMode” is “CIRCLE” and deform_ratio = 0, the boundary_end will be the original nerve trace. A value between 0 and 1 will result in a partially deformed nerve “on the way” to the final circle nerve boundary. Optional, but default value is 1 if not defined explicitly.

“Morphology”: This JSON Object is used to store information about the area and best-fit ellipse information of the nerve and fascicles (outer and inners). The user does not set values to these JSON structures, but they are a convenient record of the nerve morphometry for assigning model attributes based on the anatomy and data analysis. Units: $micrometer^2$ (area); micrometer (length). User does NOT manually set these values. Automatically populated.

5. Example:

```
{
  "sample": "Rat16-3", // monofascicular nerve without epineurium
```

```
    "sex": "M",
    "level": "Cervical",
    "samples_path": [
      "samples"
    ],
    "scale": {
      "scale_bar_length": 500,
      "shrinkage": 0.2
    },
    "modes": {
      "mask_input": "INNER_AND_OUTER_COMPILED",
      "nerve": "NOT_PRESENT",
      "deform": "NONE",
      "ci_perineurium_thickness": "MEASURED",
      "reshape_nerve": "NONE"
    },
    "Morphology": {
      "Nerve": null,
      "Fascicles": [
        {
          "outer": {
            "area": 3822.2482582148805,
            "x": 0.9345049858093262,
            "y": 0.547715425491333,
            "a": 53.227294921875,
            "b": 95.37359619140625,
            "angle": 12.440727233886719
          },
          "inners": [
            {
              "area": 3569.204913019861,
              "x": 1.0080997943878174,
              "y": 0.5349266529083252,
              "a": 51.09575653076172,
              "b": 93.090087890625,
              "angle": 12.47867107391357
            }
          ]
        }
      ]
    }
}
```

1.3 mock_sample.json

1. Named file: config/user/<mock_sample_index>.json
2. Purpose: Instructs the pipeline on which user-defined parameters to use for synthesizing nerve sample morphology inputs (i.e., 2D binary images of segmented nerve morphology) for a single sample in preparation for 3D representation of the nerve in the FEM.
3. Syntax: To declare this entity in config/user/<mock_sample_index>.json, use the following syntax:

Note: Eccentricity (e) is defined as a function of the major (a-) and minor (b-) axes as follows:

$$e = \sqrt{1 - \frac{b^2}{a^2}}$$

```
{
  "global": {
    "NAME": String
  },
  "scalebar_length": Double,
  "nerve": {
    "a_nerve": Double,
    "b_nerve": Double,
    "rot_nerve": Double
  },
  "figure": {
    "fig_margin": Double,
    "fig_dpi": Integer
  },

  // EXAMPLE POPULATE Parameters for EXPLICIT
  "populate": {
    "mode": "EXPLICIT",
    "min_fascicle_separation": 5,
    "Fascicles": [
      {
        "centroid_x": Double,
        "centroid_y": Double,
        "a": Double,
        "b": Double,
        "rot": Double
      },
      ...
    ]
```

```
  },

  // EXAMPLE POPULATE Parameters for TRUNCNORM
  "populate": {
    "mode": "TRUNCNORM",
    "mu_fasc_diam": Double,
    "std_fasc_diam": Double,
    "n_std_diam_limit": Double,
    "num_fascicle_attempt": Integer,
    "num_fascicle_placed": Integer,
    "mu_fasc_ecc": Double,
    "std_fasc_ecc": Double,
    "n_std_ecc_limit": Double,
    "max_attempt_iter": Integer,
    "min_fascicle_separation": Double,
    "seed": Integer
  },

  // EXAMPLE POPULATE Parameters for UNIFORM
  "populate": {
    "mode": "UNIFORM",
    "lower_fasc_diam": Double,
    "upper_fasc_diam": Double,
    "num_fascicle_attempt": Integer,
    "num_fascicle_placed": Integer,
    "lower_fasc_ecc": Double,
    "upper_fasc_ecc": Double,
    "max_attempt_iter": Integer,
    "min_fascicle_separation": Double,
    "seed": Integer
  }
}
```

4. Properties:

“global”: The global JSON Object contains key-value pairs to document characteristics of the sample being synthesized. Required.

- “NAME”: The value (String) of this property sets the sample name/identifier (e.g., Pig1-1) to relate to bookkeeping. The value will match the directory name in input/ containing the synthesized morphology files created by the mock_morphology_generator.py. Required.

“scalebar_length”: The value (Double, units: micrometer) is the desired length of the scale bar in the generated binary image of the scale bar (s.tif). Required.

“nerve”: The nerve JSON Object contains key-value pairs for the elliptical nerve’s size and rotation. Required.

- “a_nerve”: Value is the nerve ellipse axis ‘a’ (Double, units: micrometer). Required.
- “b_nerve”: Value is the nerve ellipse axis ‘b’ (Double, units: micrometer). Required.
- “rot_nerve”: Value is the nerve ellipse axis rotation (Double, units: degrees). Positive angles are counter-clockwise and negative are clockwise, relative to orientation with a-axis aligned with +x-direction. Required.

“figure”: The figure JSON Object contains key-value pairs for parameters that determine the size and resolution of the generated binary masks. Required.

- “fig_margin”: The value (Double, >1 otherwise an error is thrown) sets the x- and y-limits of the binary masks generated. The limits are set relative to the maximum nerve ellipse axis dimension (+/- fig_margin*max(a_nerve, b_nerve) in both x- and y-directions). Required.
- “fig_dpi”: The value (Integer) is the “dots per inch” resolution of the synthesized binary images. Higher resolutions will result in more accurate interpolation curves of inners in COMSOL. We recommend >1000 for this value. Required.

“populate”: The populate JSON Object contains key-value pairs for parameters that determine how the nerve contents (i.e., fascicle inners) are populated. Required.

- “mode”: The value (String) is the “PopulateMode” that tells the program which method to use to populate the nerve. Required.
  - As listed in Enums (S6 Text), known “PopulateModes” include:
    - “EXPLICIT”: Populates the nerve with elliptical inners that are defined explicitly by the user.
      - “min_fascicle_separation”: The value (Double, units: micrometer) determines the minimum distance between fascicle boundaries in the binary mask image that the pipeline will allow without throwing an error. This value controls the separation required between fascicles in the binary mask only. There is a separate parameter for forcing a distance (post-deformation) of the nerve between fascicles and between fascicles and the nerve boundary in sample.json. Required.
      - “Fascicles”: The value List[JSON Object] contains a JSON Object for each inner. Required. Within each JSON Object, the following key-value pairs are present:
        - “centroid_x”: Value (Double, units: micrometer) is the x-coordinate of the centroid of the best-fit ellipse of the Trace. Required.
        - “centroid_y”: Value (Double, units: micrometer) is the y-coordinate of the centroid of the best-fit ellipse of the Trace. Required.
        - “a”: Value is the ellipse axis ‘a’ (Double, units: micrometer). Required.

        - “b”: Value is the ellipse axis ‘b’ (Double, units: micrometer). Required.
        - “rot”: Value is the ellipse axis rotation (Double, units: degrees). Positive angles are counter-clockwise and negative are clockwise, relative to orientation with a-axis aligned with +x-direction. Required.

- “TRUNCNORM”: Places fascicles in the nerve with size and eccentricity randomly based on a truncated normal distribution. Rotation of fascicles is randomly drawn from uniform distribution from 0 to 360 degrees.
    - “mu_fasc_diam”: The value (Double, units: micrometer) is the mean fascicle diameter in the distribution. Required.
    - “std_fasc_diam”: The value (Double, units: micrometer) is the standard deviation of fascicle diameter in the distribution. Required.
    - “n_std_diam_limit”: The value (Double) is the limited number of standard deviations for the truncated normal fascicle diameter distribution. The value does not need to be an integer. Required.
    - “num_fascicle_attempt”: The value (Integer) is the number of different fascicles from the distribution that the program will attempt to place. Required.
        - If “num_fascicle_attempt” does not equal “num_fascicle_placed”, a warning is printed to the console instructing user to reduce the number, size, and/or separation of the fascicles.
    - “num_fascicle_placed”: The value (Integer) is the number of successfully placed fascicles in the nerve cross section. Automatically populated.
        - If “num_fascicle_attempt” does not equal “num_fascicle_placed”, a warning is printed to the console instructing user to reduce the number, size, and/or separation of the fascicles.
    - “mu_fasc_ecc”: The value (Double) is the mean fascicle eccentricity in the distribution. Must be <= 1 and > 0. Set to 1 for circles. Required.
    - “std_fasc_ecc”: The value (Double, units: micrometer) is the standard deviation of fascicle eccentricity in the distribution. Required.
    - “n_std_ecc_limit”: The value (Double) is the limited number of standard deviations for the truncated normal fascicle eccentricity distribution. Required.
    - “max_attempt_iter”: The value (Integer) is the number of different random locations within the nerve that the program will attempt to

place a fascicle before skipping it (presumably because it cannot possibly fit in the nerve). We recommend using 100+. Required.
    - "min_fascicle_separation": The value (Double, units: micrometer) determines the minimum distance between fascicle boundaries in the binary mask image that the pipeline will allow without throwing an error. This value controls the separation required between fascicles in the binary mask only. There is a separate parameter for forcing a distance (post-deformation) of the nerve between fascicles and between fascicles and the nerve boundary in sample.json. Required.
    - "seed": The value (Integer) initiates the random number generator. Required.

- "UNIFORM": Places fascicles in the nerve with size and eccentricity randomly based on a uniform distribution. Rotation of fascicles is randomly drawn from uniform distribution from 0 to 360 degrees.
    - "lower_fasc_diam": The value (Double, units: micrometer) is the lower limit of the uniform distribution for fascicle diameter. Required.
    - "upper_fasc_diam": The value (Double, units: micrometer) is the upper limit of the uniform distribution for fascicle diameter. Required.
    - "num_fascicle_attempt": The value (Integer) is the number of different fascicles from the distribution that the program will attempt to place. Required.
        - If "num_fascicle_attempt" does not equal "num_fascicle_placed", a warning is printed to the console instructing user to reduce the number, size, and/or separation of the fascicles.
    - "num_fascicle_placed": The value (Integer) is the number of successfully placed fascicles in the nerve cross section. Automatically populated.
        - If "num_fascicle_attempt" does not equal "num_fascicle_placed", a warning is printed to the console instructing user to reduce the number, size, and/or separation of the fascicles.
    - "lower_fasc_ecc": The value (Double) is the lower limit of the uniform distribution for fascicle eccentricity. Must be <= 1 and > 0. Set to 1 for circles. Required.
    - "upper_fasc_ecc": The value (Double) is the upper limit of the uniform distribution for fascicle eccentricity. Must be <= 1 and > 0. Set to 1 for circles. Required.
    - "max_attempt_iter": The value (Integer) is the number of different random locations within the nerve that the program will attempt to

place a fascicle before skipping it (presumably because it cannot possibly fit in the nerve). We recommend using 100+. Required.

- “min_fascicle_separation”: The value (Double, units: micrometer) determines the minimum distance between fascicle boundaries in the binary mask image that the pipeline will allow without throwing an error. This value controls the separation required between fascicles in the binary mask only. There is a separate parameter for forcing a distance (post-deformation) of the nerve between fascicles and between fascicles and the nerve boundary in sample.json. Required.
- “seed”: The value (Integer) initiates the random number generator. Required.

5. Example:


```
{
 "global": {
   "NAME": "Alien1-1"
 },
 scalebar_length": 1000,
 "nerve": {
   "a_nerve": 2200,
   "b_nerve": 1800,
   "rot_nerve": 0
 },
 "figure": {
   "fig_margin": 1.2,
   "fig_dpi": 1000
 },
 "populate": {
   "mode": "UNIFORM",
   "lower_fasc_diam": 400,
   "upper_fasc_diam": 500,
   "num_fascicle_attempt": 15,
   "num_fascicle_placed": 15,
   "lower_fasc_ecc": 0.5,
   "upper_fasc_ecc": 0.7,
   "max_attempt_iter": 100,
   "min_fascicle_separation": 10,
   "seed": 120
 }
}
```

1.4 model.json

1. Named file: samples/<sample_index>/models/<model_index>/model.json
2. Purpose: Contains user-defined modes and parameters to use in constructing the FEM (***Model***). We provide parameterized control of model geometry dimensions, cuff electrode, material assignment, spatial discretization of the FEM (mesh), and solution. Additionally, model.json stores meshing and solving statistics.
3. Syntax: To declare this entity in samples/<sample_index>/models/<model_index>/model.json, use the following syntax:

```
{
 "modes": {
   "rho_perineurium": String,
   "cuff_shift": String,
   "fiber_z": String,
   "use_ci": Boolean
 },
 "medium": {
   "proximal": {
     "distant_ground": Boolean,
     "length": Double,
     "radius": Double
   },
   "distal": {
     "exist": Boolean,
     "distant_ground": Boolean,
     "length": Double,
     "radius": Double,
     "shift": {
       "x": Double,
       "y": Double,
       "z": Double
     }
   }
 },
 "inner_interp_tol": Double,
 "outer_interp_tol": Double,
 "cuff": {
   "preset": String,
   "rotate": {
     "pos_ang": Double,
     "add_ang": Double
   },
   "shift": {
```

```
      "x": Double,
      "y": Double,
      "z": Double
    }
  },
  "min_radius_enclosing_circle": Double,
  "mesh": {
    "name": String,
    "shape_order": Integer,
    "proximal": {
      "type": {
        "im": String,
        "name": String
      },
      "hmax": Double,
      "hmin": Double,
      "hgrad": Double,
      "hcurve": Double,
      "hnarrow": Double,
      "mesh_time": Double
    },
    "distal": {
      "type": {
        "im": String,
        "name": String
      },
      "hmax": Double,
      "hmin": Double,
      "hgrad": Double,
      "hcurve": Double,
      "hnarrow": Double,
      "mesh_time": Double
    },
    "stats": {
      "quality_measure": String,
      "number_elements": Double,
      "min_quality": Double,
      "mean_quality": Double,
      "min_volume": Double,
      "volume": Double
    }
  },
  "frequency": Double,
  "temperature": Double,
```

```
 "conductivities": {
   "recess": String,
   "medium": String,
   "fill": String,
   "insulator": String,
   "conductor": String,
   "endoneurium": String,
   "perineurium": {
     "label": String,
     "value": Double
   },
   "epineurium": String
 },
 "solver": {
   "sorder": String,
   "name": String
 },
 "solution": {
   "sol_time": Double
 }
}
```

4. Properties:

“modes”

- “rho_perineurium”: The value (String) is the “PerineuriumResistivityMode” that tells the program how to calculate the perineurium conductivity in a frequency and/or temperature dependent way. Required.
    - As listed in Enums (S6 Text), known “PerineuriumResistivityModes” include:
        - “RHO_WEERASURIYA”: Program uses mean of circuits C and D from Weerasuriya 1984 [1] (frog sciatic nerve) (S28 Text) to adjust perineurium conductivity to account for temperature and frequency (which are both stored in model.json).
        - “MANUAL”: Program uses the user-defined value of conductivity in “conductivities” (under “perineurium”) with no automated correction for frequency.
- “cuff_shift”: The value (String) is the “CuffShiftMode” that tells the program how to shift the cuff on the nerve (S17 and S19 Text). Required.
    - As listed in Enums (S6 Text), known modes include:
        - “NAIVE_ROTATION_MIN_CIRCLE_BOUNDARY”: Program shifts the cuff to within a user-defined distance of the minimum bounding circle of the nerve sample. The direction of the shift is defined in the preset cuff JSON file (S17 and S19 Text).

    - “NAIVE_ROTATION_TRACE_BOUNDARY”: Program shifts the cuff to within a user-defined distance of the nerve trace boundary. The direction of the shift is defined in the preset cuff JSON file (S17 and S19 Text).
    - “AUTO_ROTATION_MIN_CIRCLE_BOUNDARY”: Program shifts/rotates the cuff to within a user-defined distance of the minimum bounding circle of the nerve sample to align with the Slide’s “fascicle_centroid”. The direction of the shift is defined in the preset cuff JSON file (S17 and S19 Text).
    - “AUTO_ROTATION_MIN_TRACE_BOUNDARY”: Program shifts/rotates the cuff to within a user-defined distance of the nerve trace boundary to align with the Slide’s “fascicle_centroid”. The direction of the shift is defined in the preset cuff JSON file (S17 and S19 Text).
    - “NONE”: Program keeps both the nerve centroid and cuff centered at (x,y)=(0,0) and no cuff rotation is performed (S17 and S19 Text).
- “fiber_z”: The value (String) is the “FiberZMode” that tells the program how to seed the NEURON fibers along the length of the FEM. In the current implementation of the pipeline, this value must be “EXTRUSION”. Required.
- “use_ci”: The value (Boolean), if true, tells the program whether to model fascicles (outer) that have a single inner as a sheet resistance (i.e., COMSOL “contact impedance”) to simplify the meshing of the model. If the value is false, the program will mesh the perineurium of all fascicles. Note that fascicles that have more than one inner per outer always have a meshed perineurium.

“medium”: The medium JSON Object contains information for the size, position, and boundary conditions (i.e., grounded external surface) of the proximal (i.e., cylinder containing the full length of nerve and the cuff electrode) and distal (i.e., surrounding) medium domains. Required.

- “proximal”: The proximal JSON Object has keys “distant_ground” (Boolean) that sets the outer surface to ground if true (if false, sets the outer surface to “current conservation”, i.e., perfectly insulating), “length” (Double, units: micrometer), and “radius” (Double, units: micrometer) of a cylindrical medium. The proximal cylindrical medium is centered at the origin (x,y,z)=(0,0,0), where the (x,y)-origin is the centroid of the nerve sample (i.e., best-fit ellipse of the Trace/Nerve), and extends into only the positive z-direction for the length of the nerve. A warning is printed to the console if the user sets “distant_ground” to true AND a distal domain exists (see “distal” below). Required.
- “distal”: The distal JSON Object has keys analogous to the “proximal” JSON Object, but contains additional keys “exist” (Boolean) and “shift” (JSON Object containing key-values pairs (Doubles)). The “exist” key allows the user to toggle the existence of the distal medium, which is intended to be used to apply coarser meshing parameters than in the proximal medium (which contains the nerve and cuff electrode along the full length of the nerve). The “shift” JSON Object provides control for the user to move the distal medium around the proximal medium (x,y,z) (Double, units: micrometer). Optional.

“inner_interp_tol”: The value (Double) sets the relative tolerance for the representation of the inner trace(s) in COMSOL. When the value is set to 0, the curve is jagged, and increasing the

value of this parameter increases the smoothness of the curve. COMSOL's "closed curve" setting interpolates the points of the curve with continuous first- and second-order derivatives. Generally, we find an interpolation tolerance of ~0.01 to be appropriate, but the user should check that the interpolation tolerance is set correctly for their input nerve sample morphology. Required.

"outer_interp_tol": The value (Double) sets the relative tolerance for the representation of the outer trace(s) in COMSOL. When the value is set to 0, the curve is jagged, and increasing the value of this parameter increases the smoothness of the curve. COMSOL's "closed curve" setting interpolates the points of the curve with continuous first- and second-order derivatives. Generally, we find an interpolation tolerance of ~0.01 to be appropriate, but the user should check that the interpolation tolerance is set correctly for their input nerve sample morphology. Required.

"cuff": The cuff JSON Object contains key-value pairs that define which cuff to model on the nerve in addition to how it is placed on the nerve (i.e., rotation and translation). If the user would like to loop over preset cuff designs, then they must create a ***Model*** (model index) for each cuff preset. Required.

- "preset": The value (String) indicates which cuff to model, selected from the list of filenames of the "preset" cuffs in config/system/cuffs/<filename>.json (Fig 3A and S17 Text). Required.
- "rotate": Contains two keys: "pos_ang" (automatically populated based on "CuffShiftMode", i.e., "cuff_shift" parameter in ***Model***) and "add_ang" (optionally set by user to rotate cuff by an additional angle) (S19 Text).
    - "pos_ang" (Double, units: degrees) is calculated by the pipeline for the "AUTO" CuffShiftModes (S19 Text).
    - "add_ang" (Double, units: degrees) is user-defined and adds additional rotation in the counterclockwise direction. If the parameter is not specified, the default value is 0. Optional.
- "shift": Contains three keys: "x", "y", and "z". Automatically calculated based on "CuffShiftMode".
    - Each key defines the translation of the cuff in the Cartesian coordinate system (Double, units: micrometer). The values are automatically populated by the pipeline based on the "CuffShiftMode" (i.e., "cuff_shift" parameter within "modes"). Origin (x,y)=(0,0) corresponds to the centroid of the nerve sample (or fascicle best-fit ellipse of the Trace if monofascicular), and shift in z-direction is along the proximal medium's (i.e., the nerve's) length relative to the cuff "Center" (defined in preset JSON file).

"min_radius_enclosing_circle": The value (Double, units: micrometer) is automatically defined in the program with the radius of the minimum bounding circle of the sample, which is used for placing the cuff on the nerve for certain CuffShiftModes. Automatically calculated.

"mesh": The mesh JSON Object contains key-value pairs that define COMSOL's meshing parameters (required, user-defined) and resulting meshing statistics (automatically calculated).

- "name": Mesher identity (String) to use. In current pipeline implementation, only COMSOL is supported (e.g., "COMSOL Multiphysics 5.5"). Required.
- "shape_order": Order of geometric shape functions (Integer) (e.g., quadratic = 2). Required.
- "proximal": Meshing parameters for the proximal cylindrical domain (as defined in "medium"). Required (S25 Text).
    - "type": JSON Object containing parameters/definitions specific to meshing discretization method (e.g., free tetrahedral "ftet"). We recommend free tetrahedral meshes. Required (S25 Text).
        - "im": COMSOL indexing prefix (String) (e.g., free tetrahedral "ftet"). Required.
        - "name": COMSOL system name (String) for the created mesh (e.g., "FreeTet"). Required.
    - "hmax": Maximum element size (Double, units: micrometer). We recommend between 1000-4000. Required.
    - "hmin": Minimum element size (Double, units: micrometer). We recommend 1. Required.
    - "hgrad": Maximum element growth (Double). We recommend between 1.8-2.5. Required.
    - "hcurve": Curvature factor (Double). We recommend 0.2. Required.
    - "hnarrow": Resolution of narrow regions (Double). We recommend 1. Required.
    - "mesh_time": CPU time required for the mesh to build (Double, units: milliseconds). Automatically populated.
- "distal": Meshing parameters for the distal cylindrical domain (as defined in "medium"). Required if distal domain present (see "medium").
    - "type": JSON Object containing parameters/definitions specific to meshing discretization method (e.g., free tetrahedral "ftet"). We recommend free tetrahedral meshes. Required (S25 Text).
        - "im": COMSOL indexing prefix (String) (e.g., free tetrahedral "ftet"). Required.
        - "name": COMSOL system name (String) for the created mesh (e.g., "FreeTet"). Required.
    - "hmax": Maximum element size (Double, units: micrometer). We recommend between 1000-4000. Required.
    - "hmin": Minimum element size (Double, units: micrometer). We recommend 1. Required.
    - "hgrad": Maximum element growth (Double). We recommend between 1.8-2.5. Required.
    - "hcurve": Curvature factor (Double). We recommend 0.2. Required.
    - "hnarrow": Resolution of narrow regions (Double). We recommend 1. Required.
    - "mesh_time": CPU time required for the mesh to build (Double, units: milliseconds). Automatically populated.

- “stats”: Meshing statistics. See COMSOL documentation for more details. Automatically populated.
    - “quality_measure”: (String) (e.g., “skewness”, “maxangle”, “volcircum”, “vollength”, “condition”, or “growth”)
    - “number_elements”: (Integer)
    - “min_quality”: (Double)
    - “mean_quality”: (Double)
    - “min_volume”: (Double)
    - “volume”: (Double)

“frequency”: Defines the frequency value used for frequency-dependent material conductivities (Double, unit: Hz) (S28 Text). Required only if “PerineuriumResistivityMode” is “RHO_WEERASURIYA”.

“temperature”: Defines the temperature of the nerve environment, which is used to define temperature-dependent material conductivity and ion channel mechanisms in NEURON (Double, unit: Celsius). Required (user must verify *.MOD files will adjust Q10 values for temperature, verified temperature is 37 °C).

“conductivities”: The conductivities JSON Object contains key-value pairs that assign materials (either previously defined in materials.json (String), or explicitly defined in place (JSON Object)) to material functions (i.e., cuff “insulator”, contact “conductor”, contact “recess”, cuff “fill”, “endoneurium”, “perineurium”, “epineurium”). All materials functions that are assigned to domains in the part primitives are required. If a contact primitive contains a selection for recessed domain, the recess material must be assigned even if there is no recessed domain in the preset’s parameterized implementation.

- If the material is defined in place, the JSON Object value is of the following structure:
    - “label”: Communicates type of material assigned to domains (String). The label is used to assign materials to domains (S27 Text). Required.
    - “value”: Required. Material conductivity for isotropic materials (String, unit: S/m) OR (String, “anisotropic”) with additional keys:
        - “sigma_x”, “sigma_y”, “sigma_z” each with values (String, unit: S/m). Required.

“solver”: The solver JSON Object contains key-value pairs to control the solver. Required.

- “sorder”: Order of solution shape functions (String) (e.g., quadratic). Required.
- “name”: Solver identity (String) to use. In current pipeline implementation, only COMSOL is supported (e.g., “COMSOL Multiphysics 5.5”). Required.

“solution”: The solution JSON Object contains key-value pairs to keep record of FEM solver processes. Currently, the only information stored in this JSON Object is the CPU solution time (“sol_time”, units: milliseconds). Optional.

5. Example:

{
 "modes": {
  "rho_perineurium": "RHO_WEERASURIYA",
  "cuff_shift": " AUTO_ROTATION_MIN_CIRCLE_BOUNDARY",
  "fiber_z": "EXTRUSION",
  "use_ci": true
 },
 "medium": {
  "proximal": {
   "distant_ground": false,
   "length": 25000,
   "radius": 3000
  },
  "distal": {
   "exist": true,
   "distant_ground": true,
   "length": 25000,
   "radius": 5000,
   "shift": {
    "x": 0,
    "y": 0,
    "z": 0
   }
  }
 },
 "inner_interp_tol": 0.01,
 "outer_interp_tol": 0.01,
 "cuff": {
  "preset": "CorTec300.json",
  "rotate": {
   "add_ang": 0,
   "pos_ang": 143.1780701822382
  },
  "shift": {
   "x": 13.596123436622277,
   "y": 62.55712593704458,
   "z": 0
  }
 },
 "min_radius_enclosing_circle": 78.985163169824,
 "mesh": {
  "name": "COMSOL Multiphysics 5.5",
  "shape_order": 2,
  "proximal": {

"type": {
"im": "ftet",
"name": "FreeTet"
},
"hmax": 4000,
"hmin": 10,
"hgrad": 1.8,
"hcurve": 0.2,
"hnarrow": 1,
"mesh_time": 35186.652381
},
"distal": {
"type": {
"im": "ftet",
"name": "FreeTet"
},
"hmax": 4000,
"hmin": 10,
"hgrad": 1.8,
"hcurve": 0.2,
"hnarrow": 1,
"mesh_time": 3196.938087
},
"stats": {
"quality_measure": "vollength",
"number_elements": 1171823,
"min_quality": 0.244,
"mean_quality": 0.7237,
"min_volume": 19.42,
"volume": 1954000000000.0
}
},
"frequency": 1, // in this example, no change in material properties occurs
"temperature": 37,
"conductivities": {
"recess": "saline",
"medium": "muscle",
"fill": "saline",
"insulator": "silicone",
"conductor": "platinum",
"endoneurium": "endoneurium",
"perineurium": {
"label": "RHO_WEERASURIYA @ 1 Hz",
"value": "0.0008703220191470844"

```
  },
  "epineurium": "epineurium"
 },
 "solver": {
  "sorder": "quadratic",
  "name": "COMSOL Multiphysics 5.5"
 },
 "solution": {
  "sol_time": 194028.727942
 }
}
```

1.5 sim.json

1. Named file: config/user/sims/<sim_index>.json
2. Purpose: Instructs the pipeline on which user-defined parameters to use in constructing NEURON simulation directories (***Sim***). We provide parameterized control of cuff electrode contact weighting, fiber model and placement in the FEM, extracellular stimulation waveform, intracellular stimulation, flags to indicate which outputs to save (e.g., state variables of channel gating mechanisms, transmembrane potential, intracellular stimulation), and stimulation threshold-finding protocol (S22, S32, and S33 Text).
3. Syntax: To declare this entity in config/user/sims/, use the following syntax:

```
{
 "n_dimensions": Integer,
 "active_srcs": {
  "CorTec300.json": [[Double, Double]], // for example
  "default": [[1, -1]]
 },
 "fibers": {
  "plot": Boolean,
  "mode": String,
  "xy_trace_buffer": Double,
  "z_parameters": {

   // EXAMPLE diameter parameter for defining fixed diameter fibers
   "diameter": [Double],

   // EXAMPLE diameter parameter for TRUNCNORM (i.e., diameters from a truncated normal
   // distribution) which is only compatible for “MRG_INTERPOLATION” myelinated or
   // unmyelinated fiber types)
   “diameter”: {
    “mode”: “TRUNCNORM”,
```

```
    “mu”: Double,
    “std”: Double,
    “n_std_limit”: Double,
    “seed”: Integer
  },

  // EXAMPLE diameter parameter for UNIFORM (i.e., diameters from a uniform
  // distribution) which is only compatible for “MRG_INTERPOLATION” myelinated or
  // unmyelinated fiber types)
  “diameter”: {
    “mode”: “UNIFORM”,
    “upper”: Double,
    “lower”: Double,
    “seed”: Integer
  },

  "min": Double,
  "max": Double,
  "offset": Double, // or omitted for random jitter within +/- 1 internodal length
  "seed": Integer
},

// EXAMPLE XY Parameters for CENTROID (from best-fit ellipse of the Trace)
"xy_parameters": {
  "mode": "CENTROID"
},

// EXAMPLE XY Parameters for UNIFORM_COUNT
"xy_parameters": {
  "mode": "UNIFORM_COUNT",
  "count": Integer,
  "seed": Integer
},

// EXAMPLE XY Parameters for WHEEL
"xy_parameters": {
  "mode": "WHEEL",
  "spoke_count": Integer,
  "point_count_per_spoke": Integer,
  "find_centroid": Boolean, // centroid of inner polygon
  "angle_offset": Double,
  "angle_offset_is_in_degrees": Boolean
},
```

```
    // EXAMPLE XY Parameters for EXPLICIT
    "xy_parameters": {
      "mode": "EXPLICIT"
    },

    // EXAMPLE XY Parameters for UNIFORM_DENSITY
    "xy_parameters": {
      "mode": "UNIFORM_DENSITY",
      "top_down": Boolean,

      // top_down is true
      "target_density": Double,
      "minimum_number": Integer,

      // top_down is false
      "target_number": Integer,
      "maximum_number": Integer,

      // for both top_down is true and false
      "seed": Integer
    }
  },

  "waveform": {
    "plot": Boolean,
    "global": {
      "dt": Double,
      "on": Double,
      "off": Double,
      "stop": Double
    },

    // EXAMPLE WAVEFORM for MONOPHASIC_PULSE_TRAIN
    "MONOPHASIC_PULSE_TRAIN": {
      "pulse_width": Double,
      "pulse_repetition_freq": Double,
      "digits": Integer
    },

    // EXAMPLE WAVEFORM for SINUSOID
    "SINUSOID": {
      "pulse_repetition_freq": Double,
      "digits": Integer
    },
```

```
  // EXAMPLE WAVEFORM for BIPHASIC_FULL_DUTY
  "BIPHASIC_FULL_DUTY": {
    "pulse_repetition_freq": Double,
    "digits": Integer
  },

  // EXAMPLE WAVEFORM for BIPHASIC_PULSE_TRAIN
  "BIPHASIC_PULSE_TRAIN": {
    "pulse_width": Double,
    "inter_phase": Double,
    "pulse_repetition_freq": Double,
    "digits": Integer
  },

  // EXAMPLE WAVEFORM for BIPHASIC_PULSE_TRAIN_Q_BALANCED_UNEVEN_PW
  "BIPHASIC_PULSE_TRAIN_Q_BALANCED_UNEVEN_PW": {
    "pulse_width_1": Double,
    "pulse_width_2": Double,
    "inter_phase": Double,
    "pulse_repetition_freq": Double,
    "digits": Integer
  },

  // EXAMPLE WAVEFORM for EXPLICIT
  "EXPLICIT": {
    "index": Integer,
    "dt_atol": Double,
    "period_repeats ": Integer
  }
},

"intracellular_stim": {
  "times": {
    "pw": Double,
    "IntraStim_PulseTrain_delay": Double,
    "IntraStim_PulseTrain_dur": Double
  },
  "pulse_repetition_freq": Double,
  "amp": Double,
  "ind": Integer
},
```

```
"saving": {
  "space": {
    "vm": Boolean,
    "gating": Boolean,
    "times": [Double],
  },
  "time": {
    "vm": Boolean,
    "gating": Boolean,
    "istim": Boolean,
    "locs": [Double] OR String
  }
},

// EXAMPLE PROTOCOL for FINITE_AMPLITUDES
"protocol": {
  "mode": "FINITE_AMPLITUDES",
  "initSS": Double,
  "dt_initSS": Double,
  "amplitudes": [Double, Double, ...]
},

// EXAMPLE PROTOCOL for ACTIVATION_THRESHOLD
"protocol": {
  "mode": "ACTIVATION_THRESHOLD", // String
  "initSS": Double,
  "dt_initSS": Double,
  "threshold": {
    "value": Double,
    "n_min_aps": Integer,
    "ap_detect_location": Double
  },
  "bounds_search": {
    "mode": String,
    "top": Double,
    "bottom": Double,
    "step": Double
  },
  "termination_criteria": {
    "mode": "ABSOLUTE_DIFFERENCE",
    "percent": Double
  }
},
```

```
// EXAMPLE PROTOCOL for BLOCK_THRESHOLD
"protocol": {
  "mode": "BLOCK_THRESHOLD", // String
  "initSS": Double,
  "dt_initSS": Double,
  "threshold": {
    "value": Double,
    "n_min_aps": Integer,
    "ap_detect_location": Double
  },
  "bounds_search": {
    "mode": String,
    "top": Double,
    "bottom": Double,
    "step": Double
  },
  "termination_criteria": {
    "mode": String,
    "percent": Double
  }
},

"supersampled_bases": {
  "generate": Boolean,
  "use": Boolean,
  "dz": Double,
  "source_sim": Integer
}
}
```

4. Properties:

“n_dimensions”: The value (Integer) is the number of parameters in ***Sim*** for which a list is provided rather than a single value. The user sets the number of parameters they are looping over (e.g., if looping over waveform pulse width and fiber diameter, n_dimensions = 2). We included this control to prevent the user from accidentally creating unintended NEURON simulations. The pipeline will only loop over the first n-dimensions. Required.

“active_srcs”: The value is a JSON Object containing key-value pairs of contact weightings for preset cuffs. Each value (List[List[Double]]) is the contact weighting to use to make extracellular potentials inputs to NEURON simulations. The values should not exceed +/-1 in magnitude,

otherwise an error is thrown. For monopolar cuff electrodes, the value should be either +1 or -1. For cuff electrodes with more than one contact (2+), the sum of weightings should be +1, -1, or 0. If the preset cuff is not a key in active_srcs, the list of contact weightings for the “default” key is used. Required. The potentials/ for a single fiber are calculated in the following way for the default weighting:

"default": [[1, -1]] // [[weight$_1$ (for src 1 on), weight$_2$ (for src 2 on)]]

$$potentials = (weight_1) * bases_1(x, y, z) + (weight_2) * bases_2(x, y, z)$$
$$potentials = (1) * bases_1(x, y, z) + (-1) * bases_2(x, y, z)$$

The value of potentials/ is applied to a model fiber in NEURON multiplied by the stimulation amplitude, which is either from a list of finite amplitudes or a binary search for thresholds (S22 Text):

$$V_e = (amplitude) * potentials$$

“fibers”: The value is a JSON Object containing key-value pairs that define how potentials are sampled in the FEM for application as extracellular potentials in NEURON (i.e., the Cartesian coordinates of the midpoint for each compartment (i.e., section or segment) along the length of the fiber). Required.

- “plot”: The value (Boolean) tells the program to plot the fiber (x,y)-coordinates for the user to inspect. A figure window pops up on the user’s screen during the pipeline run. Required.
- “mode”: The value (String) is the “FiberGeometry” mode that tells the program which fiber geometries to simulate in NEURON (S21 and S32 Text). Required.
    - As listed in Enums (S6 Text), known modes include:
        - “MRG_DISCRETE” (published MRG fiber model)
        - “MRG_INTERPOLATION” (interpolates the discrete diameters from published MRG fiber models)
        - “TIGERHOLM” (published C-fiber model)
        - “SUNDT” (published C-fiber model)
        - “RATTAY” (published C-fiber model)
    - For a user to simulate a novel fiber type using the pipeline, they must define the spatial discretization of points within the config/system/fiber_z.json file to match the dimensions of the fiber compartments connected in NEURON. The “FiberGeometry” mode and parameters in the “fibers” JSON Object in ***Sim*** must match the keys in config/system/fiber_z.json to select and define a fiber type (e.g., MRG requires specification of fiber “diameters”).
- “xy_trace_buffer”: The value (Double, units: micrometer) indicates the minimum required distance between the (x,y)-coordinates of a given fiber and the inner’s boundary. Since the domain boundaries are modeled in COMSOL as an interpolation curve, the exact morphology boundary coordinates read into COMSOL will be very close to (but not exactly equal to) those used in Python to seed fiber locations. To protect against

instances of fibers falling within the nerve boundary traces in the exact Python traces, but not in COMSOL's interpolation of those traces, we provided this parameter. Required.

- "z_parameters": The value is a JSON Object containing key-value pairs to instruct the system in seeding fibers along the length of the nerve. Required.
    - "diameter": The value can take multiple forms to define the fiber diameter that the user is simulating in NEURON (S21 and S32 Text). The value can control simulation of either fixed diameter fibers or fibers chosen from a distribution of diameters (note: simulating a distribution of fiber diameters is only compatible with "MRG_INTERPOLATION" myelinated or unmyelinated fiber types, not "MRG_DISCRETE"). In Sim, only one mode of defining fiber diameters can be used. Required.
        - Fixed diameter: The value (Double or List[Double], units: micrometer) is the diameter of the fiber models. If using with "MRG_DISCRETE", the diameters must be members of the set of published diameters.
        - Distribution of diameters: The value is a dictionary of key-value pairs to define the distribution of diameters based on the "mode" parameter, which can be either "TRUNCNORM" or "UNIFORM".
            - "TRUNCNORM"
                - "mode": "TRUNCNORM" (String). Required.
                - "mu": The value (Double, units micrometer) is the mean diameter of the truncated normal distribution. Required.
                - "std": The value (Double, units micrometer) is the diameter standard deviation of the truncated normal distribution. Required.
                - "n_std_limit": The value (Double) is the number of standard deviations from the mean to bound the truncated normal distribution. Required.
                - "seed": The value (Integer, unitless) seeds the random number generator before sampling fiber diameters.
            - "UNIFORM"
                - "mode": "UNIFORM" (String). Required.
                - "upper": The value (Double, units micrometer) is the upper limit on the distribution of diameters. Required.
                - "lower": The value (Double, units micrometer) is the lower limit on the distribution of diameters. Required.
                - "seed": The value (Integer) seeds the random number generator before sampling fiber diameters.
    - "min": the value (Double or List[Double], units: micrometer) is the distal extent of the seeded fiber along the length of the nerve closer to z = 0. Optional: if min and max are not both provided then the fiber length is assumed to be the proximal medium length (see model.json).
    - "max": The value (Double or List[Double] , units: micrometer) is the distal extent of the seeded fiber along the length of the nerve closer to z = length of the

proximal medium (as defined in model.json, the length of the nerve). Optional: if min and max are not both provided then the fiber length is assumed to be the proximal medium length by default.

- "offset": The value (Double or List[Double]) is the fraction of the node-node length (myelinated fibers) or segment length (unmyelinated fibers) that the center coordinate of the fiber is shifted along the z-axis from the longitudinal center of the proximal medium. Optional, but if not defined, the offset will be randomly selected between +/- 0.5 section/segment length; to avoid the randomized longitudinal placement, set the offset value to '0' for no offset.
- "seed": The value (Integer) seeds the random number generator before any random offsets are created. Required only if "offset" is not defined, in which case the program will use a random offset.

- "xy_parameters": The value is a JSON Object containing key-value pairs to instruct the system in seeding fiber locations at which to sample potentials inside inners in the nerve cross section (Fig 3B). Include only *one* version of this block in your sim.json file. Required.
  - "mode": The value (String) is the "FiberXYMode" that tells the program how to seed fiber locations inside each inner in the nerve cross section. Required.
    - As listed in Enums (S6 Text), known modes include:
      - "CENTROID": Place one fiber at the centroid (i.e., from the best-fit ellipse of the inner) of each inner.
        - No parameters required.
      - "UNIFORM_COUNT": Randomly place the same number of fibers in each inner, regardless of inner's size.
        - "count": The value (Integer) is the number of fibers per inner. Required.
        - "seed": The value (Integer) is the seed for the random number generator that is instantiated before the point picking algorithm for fiber (x,y)-coordinates starts. Required.
      - "WHEEL": Place fibers in each inner following a pattern of radial spokes out from the geometric centroid.
        - "spoke_count": The value (Integer) is the number of radial spokes. Required.
        - "point_count_per_spoke": The value (Integer) is the number of fibers to place on each spoke out from the centroid (i.e., excluding the centroid). Required.
        - "find_centroid": The value (Boolean), if true, tells the program to include the geometric centroid as a fiber coordinate. Required.
        - "angle_offset": The value (Double, either degrees or radians depending on next parameter, "angle_offset_is_in_degrees") sets the direction of the first

spoke in the wheel. The rest of the spokes are equally spaced (radially) based on the “spoke_count”. Required.

        - “angle_offset_is_in_degrees”: The value (Boolean), if true, tells the program to interpret “angle_offset” as degrees. If false, the program interprets “angle_offset” in radians. Required.
    - “EXPLICIT”: The mode looks for the “explicit.txt” file that the user saved in the simulation directory (samples/<sample_index>/models/<model_index>/sims/<sim index>/) for user-specified fiber (x,y)-coordinates (see config/templates/explicit.txt). Note, this file is only required if the user is using the “EXPLICIT” “FiberXYMode”. An error is thrown if any explicitly defined coordinates are not inside any inners.
        - No parameters required.
    - “UNIFORM_DENSITY”: Place fibers randomly in each inner to achieve a consistent number of fibers per unit area.
        - “top_down”: The value (Boolean) dictates how the pipeline determines how many fibers to seed in each inner. Required.
            - If “top_down” is true, the program determines the number of fibers per inner with the “target_density”. If the number of fibers in an inner is less than the “minimum_number”, then the minimum number is used.
                - “target_density”: The value (Double) is the density (fibers/μm^2). Required only if “top_down” is true.
                - “minimum_number”: The value (Integer) is the minimum number of fibers that the program will place in an inner. Required only if “top_down” is true.
            - If “top_down” is false (i.e., bottom-up), the program determines the number of fibers per unit area (i.e., fiber density) with “target_number” and the area of the smallest inner. If the number of fibers in an inner is more than the “maximum_number”, then the maximum number is used.
                - “target_number”: The value (Integer) is the number of fibers placed in the smallest inner. Required only if “top_down” is false.
                - “maximum_number”: The value (Integer) is the maximum number of fibers placed in the largest inner. Required only if “top_down” is false.

        - “seed”: The value (Integer) is the seed for the random number generator that is instantiated before the point picking algorithm for fiber (x,y)-coordinates starts. Required.

“waveform”: The waveform JSON Object contains key-value pairs to instruct the system in setting global time discretization settings and stimulation waveform parameters (Fig 3C). Required.

- “plot”: The value (Boolean) turns plotting of the waveform to a figure on/off as each waveform is written to file. A figure is displayed to the user’s screen during the pipeline run.
- “global”: the value (JSON Object) contains key-value pairs that define NEURON time discretization parameters. Required.
    - “dt”: The value (Double, units: milliseconds) is the time step used in the NEURON simulation for fiber response to electrical stimulation. Required.
    - “on”: The value (Double, units: milliseconds) is the time when the extracellular stimulation is turned on. Required.
    - “off”: The value (Double, units: milliseconds) is the time when the extracellular stimulation is turned off. Required.
    - “stop”: The value (Double, units: milliseconds) is the time when the simulation stops. Required.

The user must also provide **one** of the following JSON Objects containing “WaveformMode”-specific parameters. The user can only loop parameters for one type of waveform in a ***Sim***.

Note: the “digits” parameter for the following “WareformModes” sets the precision of the unscaled current amplitude. For waveforms that are only ever +/-1 and 0 (e.g., MONOPHASIC_PULSE_TRAIN, BIPHASIC_FULL_DUTY, BIPHASIC_PULSE_TRAIN), the user can represent the waveform faithfully with 1 digit of precision. However, for waveforms that assume intermediate values (e.g., SINUSOID, BIPHASIC_PULSE_TRAIN_Q_BALANCED_UNEVEN_PW, EXPLICIT) more digits of precision may be required to achieve numerical accuracy. An excessive number of digits of precision will increase computational load and waste storage resources.

Note: if one of the parameter values in the “WaveformMode” JSON Object is a list, then there are n_sim/ folders created for as many waveforms as parameter values in the list. If more than one parameter value is a list, then there are n_sim/ folders created for each combination of waveform parameters among the lists (i.e., the Cartesian product).

- “MONOPHASIC_PULSE_TRAIN”
    - “pulse_width”: The value (Double, or List[Double], units: milliseconds) is the duration (“width”) of the monophasic rectangular pulse. Required.
    - “pulse_repetition_freq”: The value (Double, or List[Double], units: Hz) is the rate at which individual pulses are delivered. Required.

  - "digits": The value (Integer) is the number of digits of precision used in saving the waveform to file. Required.
- "SINUSOID"
  - "pulse_repetition_freq": The value (Double, or List[Double], units: Hz) is the frequency of the sinusoid. Required.
  - "digits": The value (Integer) is the number of digits of precision used in saving the waveform to file. Required.
- "BIPHASIC_FULL_DUTY": This waveform consists of biphasic symmetric rectangular pulses, where there is no "off" time between repetitions of the biphasic pulse, hence the "full duty cycle" designation. Thus, the phase width (i.e., the duration of one phase in the symmetric pulse) is equal to half of the period, as defined by the specified "pulse_repetition_freq". This waveform is a special case of "BIPHASIC_PULSE_TRAIN_Q_BALANCED_UNEVEN_PW" (below).
  - "pulse_repetition_freq": The value (Double, or List[Double], units: Hz) is the rate at which individual pulses are delivered. Required.
  - "digits": The value (Integer) is the number of digits of precision used in saving the waveform to file. Required.
- "BIPHASIC_PULSE_TRAIN": This waveform consists of biphasic symmetric rectangular pulses, where the phase width (i.e., the duration of one phase of the symmetric pulse) is defined by "pulse_width" and the two phases of the biphasic symmetric pulses may be spaced by a gap defined by "inter_phase". This waveform is a special case of "BIPHASIC_PULSE_TRAIN_Q_BALANCED_UNEVEN_PW" (below).
  - "pulse_width": The value (Double, or List[Double], units: milliseconds) is the duration ("width") of one phase in the biphasic rectangular pulse. Required.
  - "inter_phase": The value (Double, or List[Double], units: milliseconds) is the duration of time between the first and second phases in the biphasic rectangular pulse. Required.
  - "pulse_repetition_freq": The value (Double, or List[Double], units: Hz) is the rate at which individual pulses are delivered. Required.
  - "digits": the value (Integer) is the number of digits of precision used in saving the waveform to file. Required.
- "BIPHASIC_PULSE_TRAIN_Q_BALANCED_UNEVEN_PW": This waveform consists of biphasic rectangular pulses that are charged-balanced (i.e., the charge of the first phase is equal to the charge of the second phase), but can be defined to be asymmetrical, such that the two phases can have different durations, as defined by "pulse_width_1" and "pulse_width_2". Further, the two phases of the biphasic pulses may be spaced by a gap defined by "inter_phase".
  - "pulse_width_1": The value (Double, or List[Double], units: milliseconds) is the duration ("width") of the first phase (positive amplitude) in the biphasic rectangular pulse. Required.
  - "pulse_width_2": The value (Double, or List[Double], units: milliseconds) is the duration ("width") of the second phase (negative amplitude) in the biphasic rectangular pulse. Required.

- "inter_phase": The value (Double, or List[Double], units: milliseconds) is the duration of time between the primary and secondary phases in the biphasic rectangular pulse (amplitude is 0). Required.
- "pulse_repetition_freq": The value (Double, or List[Double], units: Hz) is the rate at which individual pulses are delivered. Required.
- "digits": The value (Integer) is the number of digits of precision used in saving the waveform to file. Required.

- "EXPLICIT"
  - "index": The value (Integer) is the index of the explicit user-provided waveform stored in config/user/waveforms/<waveform index>.dat with the time step on the first line followed by the current amplitude value (unscaled, maximum amplitude +/- 1) at each time step on subsequent lines. Required.
  - "dt_atol": The value (Double, units: milliseconds) is the tolerance allowed between the time step defined in the explicit waveform file and the timestep used in the NEURON simulations (see "global" above). If the difference in time step is larger than "dt_atol", the user's explicit waveform is interpolated and resampled at the "global" timestep used in NEURON using SciPy's Signal Processing package (scipy.signal) [2]. Required.
  - "period_repeats": The number of times (Integer) the input signal is repeated between when the stimulation turns "on" and "off". The signal is padded with zeros between simulation start (i.e., t=0) and "on", as well as between "off" and "end". Additionally, the signal is padded with zeros between "on" and "off" to accommodate for any extra time after the number of period repeats and before "off". Required.

"intracellular_stim": The value (JSON Object) contains key-value pairs to define the settings of the monophasic pulse train of the intracellular stimulus (S31 and S32 Text). Required.

- "times": The key-value pairs define the time durations characteristic of the intracellular stimulation. Required.
  - "pw": The value (Double, units: milliseconds) defines the pulse duration of the intracellular stimulation. Required.
  - "IntraStim_PulseTrain_delay": The value (Double, units: milliseconds) defines the delay from the start of the simulation (i.e., t=0) to the onset of the intracellular stimulation. Required.
  - "IntraStim_PulseTrain_dur": The value (Double, units: milliseconds) defines the duration from the start of the simulation (i.e., t=0) to the end of the intracellular stimulation. Required.
- "pulse_repetition_freq": The value (Double, units: Hz) defines the intracellular stimulation frequency. Required.
- "amp": The value (Double, units: nA) defines the intracellular stimulation amplitude. Required.
- "ind": The value (Integer) defines the section index (unmyelinated) or node of Ranvier number (myelinated) receiving the intracellular stimulation. The number of

sections/nodes of Ranvier is indexed from 0 and starts at the end of the fiber closest to z = 0. Required.

“saving”: The value (JSON Object) contains key-value pairs to define which state variables NEURON will save during its simulations and at which times/locations (S31 and S32 Text). Required.

- “space”:
  - “vm”: The value (Boolean), if true, tells the program to save the transmembrane potential at all segments (unmyelinated) and sections (myelinated) at the time stamps defined in “times” (see below). Required.
  - “gating”: The value (Boolean), if true, tells the program to save channel gating parameters at all segments (unmyelinated) and sections (myelinated) at the time values defined in “times” (see below). Note: Only implemented for MRG fibers. Required.
  - “times”: The value (List[Double], units: milliseconds) contains the times in the simulation at which to save the values of the state variables (i.e., “gating” or “vm”) that the user has selected to save for all segments (unmyelinated) and sections (myelinated). Required.
- “time”:
  - “vm”: The value (Boolean), if true, tells the program to save the transmembrane potential at each time step at the locations defined in “locs” (see below). Required.
  - “gating”: The value (Boolean), if true, tells the program to save the channel gating parameters at each time step at the locations defined in “locs” (see below). Note: Only implemented for MRG fibers. Required.
  - “istim”: The value (Boolean), if true, tells the program to save the applied intracellular stimulation at each time step. Required.
  - “locs”: The value (List[Double] or String, units: none) contains the locations (defined as a decimal percentage, e.g., 0.1 = 10% along fiber length) at which to save the values of the state variables that the user has selected to save for all timesteps. Alternatively, the user can use the value “all” (String) to prompt the program to save the state variables at all segments (unmyelinated) and sections (myelinated). Required.

“protocol”:

- “mode”: The value (String) is the “NeuronRunMode” that tells the program to run activation thresholds, block thresholds, or a list of extracellular stimulation amplitudes (S31 and S32 Text). Required.
  - As listed in Enums (S6 Text), known “NeuronRunModes” include:
    - “ACTIVATION_THRESHOLDS”
    - “BLOCK_THRESHOLDS”
    - “FINITE_AMPLITUDES”

- “initSS”: The value (Double, hint: should be negative or zero, units: milliseconds) is the time allowed for the system to reach steady state before starting the simulation proper. Required.
- “dt_initSS”: The value (Double, units: milliseconds) is the time step (usually larger than “dt” in “global” JSON Object (see above)) used to reach steady state in the NEURON simulations before starting the simulation proper. Required.
- “amplitudes”: The value (List[Double], units: mA) contains extracellular current amplitudes to simulate. Required if running “FINITE_AMPLITUDES” for “NeuronRunMode”.
- “threshold”: The JSON Object contains key-value pairs to define what constitutes threshold being achieved. Required for threshold finding protocols (i.e., “ACTIVATION_THRESHOLDS” and “BLOCK_THRESHOLDS”) only.
    - “value”: The value (Double, units: mV) is the transmembrane potential that must be crossed with a rising edge for the NEURON code to count an action potential. Required for threshold finding protocols (i.e., “ACTIVATION_THRESHOLDS” and “BLOCK_THRESHOLDS”) only.
    - “n_min_aps”: The value (Integer) is the number of action potentials that must be detected for the amplitude to be considered above threshold. Required for threshold finding protocols (i.e., “ACTIVATION_THRESHOLDS” and “BLOCK_THRESHOLDS”) only.
    - “ap_detect_location”: The value (Double) is the location (range 0 to 1, i.e., 0.9 is 90% of the fiber length in the +z-direction) where action potentials are detected for threshold finding protocols (i.e., “ACTIVATION_THRESHOLDS” or “BLOCK_THRESHOLDS”). Note: If using fiber models with passive end nodes, the user should not try to detect action potentials at either end of the fiber. Required for threshold finding protocols (i.e., “ACTIVATION_THRESHOLDS” and “BLOCK_THRESHOLDS”) only.
- “bounds_search”: the value (JSON Object) contains key-value pairs to define how to search for upper and lower bounds in binary search algorithms (S22 Text). Required for threshold finding protocols (i.e., “ACTIVATION_THRESHOLDS” and “BLOCK_THRESHOLDS”).
    - “mode”: the value (String) is the “SearchAmplitudeIncrementMode” that tells the program how to change the initial upper and lower bounds for the binary search; the bounds are adjusted iteratively until the initial upper bound (i.e., “top”) activates/blocks and until the initial lower bound does not activate/block, before starting the binary search (S22 Text). Required.
        - As listed in Enums (S6 Text), known “SearchAmplitudeIncrementModes” include:
            - “ABSOLUTE_INCREMENT”: If the current upper bound does not activate/block, increase the upper bound by a fixed “step” amount (e.g., 0.001 mA). If the lower bound activates/blocks, decrease the lower bound by a fixed “step” amount (e.g., 0.001 mA).
            - “PERCENT_INCREMENT”: If the upper bound does not activate/block, increase the upper bound by a “step” percentage

(e.g., 10 for 10%). If the lower bound activates/blocks, decrease the lower bound by a "step" percentage (e.g., 10 for 10%).

  - "top": The value (Double) is the upper-bound stimulation amplitude first tested in a binary search for thresholds. Required.
  - "bottom": The value (Double) is the lower-bound stimulation amplitude first tested in a binary search for thresholds. Required.
  - "step": the value (Double) is the incremental increase/decrease of the upper/lower bound in the binary search. Required.
    - If "ABSOLUTE_INCREMENT", the value (Double, unit: mA) is an increment in milliamps.
    - If "PERCENT_INCREMENT", the value (Double, units: %) is a percentage (e.g., 10 is 10%).

- "termination_criteria": Required for threshold finding protocols (i.e., "ACTIVATION_THRESHOLDS" and "BLOCK_THRESHOLDS") (S22 Text).
  - "mode": The value (String) is the "TerminationCriteriaMode" that tells the program when the upper and lower bound have converged on a solution of appropriate precision. Required.
    - As listed in Enums (S6 Text), known "TerminationCriteriaModes" include:
      - "ABSOLUTE_DIFFERENCE": If the upper bound and lower bound in the binary search are within a fixed "tolerance" amount (e.g., 0.001 mA), the upper bound value is threshold.
        - "tolerance": The value (Double) is the absolute difference between upper and lower bound in the binary search for finding threshold (unit: mA). Required.
      - "PERCENT_DIFFERENCE": If the upper bound and lower bound in the binary search are within a relative "percent" amount (e.g., 1%), the upper bound value is threshold. This mode is generally recommended as the ABSOLUTE_DIFFERENCE approach requires adjustment of the "tolerance" to be suitable for different threshold magnitudes.
        - "percent": The value (Double) is the percent difference between upper and lower bound in the binary search for finding threshold (e.g., 1 is 1%). Required.

"supersampled_bases": Optional. Required only for either generating or reusing super-sampled bases. This can be a memory efficient process by eliminating the need for long-term storage of the bases/ COMSOL *.mph files. Control of "supersampled_bases" belongs in ***Sim*** because the (x,y)-fiber locations in the nerve are determined by ***Sim***. The potentials are sampled densely along the length of the nerve at (x,y)-fiber locations once so that in a future pipeline run different fiber types can be simulated at the same location in the nerve cross section without loading COMSOL files into memory.

- “generate”: The value (Boolean) indicates if the program will create super-sampled fiber coordinates and super-sampled bases (i.e., sampled potentials from COMSOL). Required only if generating ss_bases/.
- “use”: The value (Boolean) if true directs the program to interpolate the super-sampled bases to create the extracellular potential inputs for NEURON. If false, the program will sample along the length of the COMSOL FEM at the coordinates explicitly required by “fibers”. Required only if generating ss_bases/.
- “dz”: The value (Double, units: micrometer) is the spatial sampling of the super-sampled bases. Required only if generating ss_bases/.
- “source_sim”: The value (Integer) is the ***Sim*** index that contains the super-sampled bases. If the user sets both “generate” and “use” to true, then the user should indicate the index of the current ***Sim*** here. Required only if generating ss_bases/.

5. Example:

```
{
 "n_dimensions": 3,
 "active_srcs": {
   "CorTec300.json": [[1, -1]]
 },
 "fibers": {
   "plot": false,
   "mode": "MRG_DISCRETE",
   "xy_trace_buffer": 5.0,
   "z_parameters": {
     "diameter": [1, 2, 5.7, 7.3, 8.7, 10],
     "min": 0,
     "max": 12500,
     "offset": 0,
     "seed": 123
   },
   "xy_parameters": {
     "mode": "UNIFORM_DENSITY",
     "top_down": true,
     "minimum_number": 1,      // top_down is true only
     "target_density": 0.0005, // top_down is true only
     "maximum_number": 100,    // top_down is false only
     "target_number": 50,      // top_down is false only
     "seed": 123
   }
 },
 "waveform": {
   "plot": false,
   "global": {
     "dt": 0.001,
```

```
      "on": 1,
      "off": 49,
      "stop": 50
    },
    "BIPHASIC_PULSE_TRAIN": {
      "pulse_width": 0.1,
      "inter_phase": 0,
      "pulse_repetition_freq": 1,
      "digits": 1
    }
  },
  "intracellular_stim": {
    "times": {
      "pw": 0,
      "IntraStim_PulseTrain_delay": 0,
      "IntraStim_PulseTrain_dur": 0
    },
    "pulse_repetition_freq": 0,
    "amp": 0,
    "ind": 2
  },
  "saving": {
    "space": {
      "vm": false,
      "gating": false,
      "times": [0],
    },
    "time": {
      "vm": false,
      "gating": false,
      "istim": false,
      "locs": [0]
    }
  },
  "protocol": {
    "mode": "ACTIVATION_THRESHOLD",
    "initSS": -200,
    "dt_initSS": 10,
    "threshold": {
      "value": -30,
      "n_min_aps": 1,
      "ap_detect_location": 0.9
    },
    "bounds_search": {
```

```
    "mode": "PERCENT_INCREMENT",
    "top": -0.1,
    "bottom": -0.01,
    "step": 10
  },
  "termination_criteria": {
    "mode": "PERCENT_DIFFERENCE",
    "percent": 1
  }
 }
 "supersampled_bases": {
  "generate": true,
  "use": true,
  "dz": 1,
  "source_sim": 1
  }
}
```

1.6 query_criteria.json

1. Named file: config/user/query_criteria/<query criteria index>.json
2. Purpose: Used to guide the Query class's searching algorithm in the run() and _match() methods. This is used for pulling ***Sample***, ***Model***, or ***Sim*** indices for data analysis. The query_criteria.json dictates if a given ***Sample***, ***Model***, or ***Sim*** fit the user's restricted parameter values (S33 Text).
3. Syntax

```
{
 "partial_matches": Boolean,
 "include_downstream": Boolean,
 "sample": { // can be empty, null, or omitted
 },
 "model": {  // can be empty, null, or omitted
 },
 "sim": {    // can be empty, null, or omitted
 },
 "indices": {
  "sample": null, Integer, or [Integer, ...],
  "model": null, Integer, or [Integer, ...],
  "sim": null, Integer, or [Integer, ...]
 }
}
```

4. Properties:

“partial_matches”: The value (Boolean) indicates whether Query should return configuration indices for ***Sample***, ***Model***, or ***Sim*** that are a partial match (i.e., a subset of the parameters were found, but not all).

“include_downstream’: The value (Boolean) indicates whether Query should return indices of downstream (***Sample*** > ***Model*** > ***Sim***) configurations that exist if match criteria are not provided for them.

“sample”: The value is a JSON Object that mirrors the path to the parameters of interest in ***Sample*** and their value(s).

“model”: The value is a JSON Object that mirrors the path to the parameters of interest in ***Model*** and their value(s).

“sim”: The value is a JSON Object that mirrors the path to the parameters of interest in ***Sim*** and their value(s).

“indices”:

- “sample”: The value (null, Integer, or [Integer, …]) for explicitly desired ***Sample*** indices
- “model”: The value (null, Integer, or [Integer, …]) for explicitly desired ***Model*** indices
- “sim”: The value (null, Integer, or [Integer, …]) for explicitly desired ***Sim*** indices

Note: you can have BOTH lists of desired ***Sample***, ***Model***, and ***Sim*** indices AND search criteria in one query_criteria.json.

5. Example:

```
{
 "partial_matches": true,
 "include_downstream": true,
 "sample": {
   "sample": "Rat16-3"
 },
 "model": {
   "medium": {
     "proximal": {
       "length": [1000, 2000]
     }
   }
 },
 "sim": null,
 "indices": {
```

```
  "sample": null,
  "model": null,
  "sim": null
 }
}
```

1.7 env.json

1. Named file: config/system/env.json
2. Purpose: The file contains key-value pairs for paths. The file can be automatically populated by running env_setup.py (S2 Text). Note that we have prepended all the keys in this file with “ASCENT” because these key-value pairs are directly stored as environment variables, so the “ASCENT” key distinguishes these pairs from other paths that may be present on your computer.
3. Syntax: To declare this entity in config/system/env.json, use the following syntax:

```
{
 "ASCENT_COMSOL_PATH": String,
 "ASCENT_JDK_PATH": String,
 "ASCENT_PROJECT_PATH": String,
 "ASCENT_NSIM_EXPORT_PATH": String
}
```

4. Properties:

“ASCENT_COMSOL_PATH”: The value (String) is the path for your local COMSOL installation.
“ASCENT_JDK_PATH”: The value (String) is the path for your local Java JDK installation.
“ASCENT_PROJECT_PATH”: The value (String) is the path for your local ASCENT repository.
“ASCENT_NSIM_EXPORT_PATH”: The value (String) is the path where the pipeline will save NEURON simulation directories to submit.

5. Example:

- Windows:

```
{
 "ASCENT_COMSOL_PATH": "C:\\Program Files\\COMSOL\\COMSOL55\\Multiphysics",
 "ASCENT_JDK_PATH": "C:\\Program Files\\Java\\jdk1.8.0_221\\bin",
 "ASCENT_PROJECT_PATH": "D:\\Documents\\ascent",
 "ASCENT_NSIM_EXPORT_PATH": "D:\\Documents\\ascent\\submit"
}
```

- MacOS

```
{
```

```
 "ASCENT_COMSOL_PATH": "/Applications/COMSOL55/Multiphysics ",
 "ASCENT_JDK_PATH":
"/Library/Java/JavaVirtualMachines/jdk1.8.0_221.jdk/Contents/Home/bin/",
 "ASCENT_PROJECT_PATH": "/Users/ericmusselman/Documents/ascent",
 "ASCENT_NSIM_EXPORT_PATH": "/Users/ericmusselman/Documents/ascent/submit"
}
```

1.8 exceptions.json

1. Named file: config/system/exceptions.json
2. Purpose: The file contains a list of JSON Objects, one for each documented pipeline exception. Note that this file is a single large Array, so it is wrapped in square brackets, as opposed to all other JSON files, which are wrapped in curly braces.
3. Syntax: To declare this entity in config/system/env.json, use the following syntax:

```
[
 {
   "code": Integer,
   "text": String,
   "source": String
 },
 ...
]
```

4. Properties:

“code”: The value (Integer) is an identifier that enables easy reference to a specific exception using Exceptionable’s self.throw(<code_index>). This value must be unique because the Exeptionable class uses this value to find any given exception. We suggest that you increment the code with each successive exception (akin to indexing), but any number will work as long as its value is unique.

“text”: The value (String) is a message to the user explaining why the pipeline failed.

“source”: The value (String) is a message to the user telling them where the error occurred.

5. Example:

```
[
 {
   "code": 0,
   "text": "Invalid code (out of bounds, starting at index 1).",
   "source": "src.utils.Exceptionable"
```

```
 },
 ...
]
```

1.9 materials.json

1. Named file: config/system/materials.json
2. Purpose: Stores default material and tissue properties in the pipeline for use in the 3D FEM.
3. Syntax: To declare this entity in config/system/materials.json, use the following syntax:

```
{
  "conductivities": {
    "endoneurium": { // example syntax for anisotropic medium
      "value": "anisotropic",
      "sigma_x": String,
      "sigma_y": String,
      "sigma_z": String,
      "unit": "String",
      “references”: {
        “1”: String,
        …,
        “n”: String
      }
   },
   "epineurium": { // example syntax for isotropic medium
     "value": "String",
     "unit": "String",
     “references”: {
       “1”: String,
       …,
       “n”: String
     }
   }
}
```

4. Properties:

“<material>”: The value is a JSON Object containing the conductivity value and units as Strings. Though using strings may seem odd for storing conductivity values, we do this because we read them directly into COMSOL to define materials, and COMSOL expects a string (which it evaluates as expression).

- “value”: The conductivity of the material (if not “anisotropic”, the value must be in units S/m). If the value is “anisotropic”, the system is expecting additional keys for the values in each Cartesian direction:
    - “sigma_x”: The value (String) is the conductivity in the x-direction (unit: S/m)
    - “sigma_y”: The value (String) is the conductivity in the y-direction (unit: S/m)
    - “sigma_z”: The value (String) is the conductivity in the z-direction (unit: S/m)
- “unit”: The unit of the associated conductivity in square brackets (must be “[S/m]”)
- “references”: The value (Dictionary) contains citations to the source of the material conductivity used. The contents are non-functional (i.e., they are not used in any of the code), but they serve as a point of information reference for good bookkeeping. Each reference used is assigned its own key-value pair (Optional).

5. Example:

- See: config/system/materials.json to see all built-in material definitions, which the user may add to.

```
{
  "conductivities": {
    "endoneurium": {
      "value": "anisotropic",
      "sigma_x": "1/6",
      "sigma_y": "1/6",
      "sigma_z": "1/1.75",
      "unit": "[S/m]"
    },
    "epineurium": {
      "value": "1/6.3",
      "unit": "[S/m]"
    }
  }
}
```

Note: Perineurium can be represented in the pipeline as either a meshed domain with a finite thickness or as a thin layer approximation, but the conductivity value used for either method is defined in materials.json unless the “PerineuriumResistivityMode” is “MANUAL” and the conductivity is defined explicitly in ***Model*** (S28 Text).

1.10 ci_peri_thickness.json

1. Named file: config/system/ci_peri_thickness.json
2. Purpose: The file stores “PerineuriumThicknessMode” definitions that are referenced in sample.json (“ci_perineurium_thickness”) for assigning perineurium thickness values to fascicles for a mask of inners if “ci_perineurium_thickness” is not specified as

"MEASURED". The calculated thickness may be explicitly built in the FEM geometry and meshed (i.e., if "use_ci" in ***Model*** is false) or may only be used for calculating the contact impedance if modeling the perineurium with a thin layer approximation (S14 and S28 Text).

3. Syntax:

```
{
 "ci_perineurium_thickness_parameters": {
   "GRINBERG_2008": {
     "a": Double,
     "b": Double
   },
   ...
}
```

4. Properties:

"<PerineuriumThicknessMode>": JSON Object that contains key-value pairs defining the relationship between fascicle diameter (micrometers) and perineurium thickness. Required.

- "a": Value (Double, units: µm/µm) as in thickness = a*x + b
- "b": Value (Double, units: µm) as in thickness = a*x + b

5. Example:
    - See: config/system/ci_peri_thickness.json to see all built-in PerineuriumThicknessMode relationships.

1.11 mesh_dependent_model.json

1. Named file: config/system/mesh_dependent_model.json
2. Purpose: This file is not to be changed unless a user adds new parameters to ***Model***. The use of this file happens behind the scenes. The file informs the ModelSearcher class (S26 Text) if two model configurations constitute a "mesh match" (i.e., that the mesh from a previously solved and identical model can be recycled). Note that if you modify the structure of model.json, the pipeline expects this file's structure to be changed as well. If the Boolean for a parameter is true, then the parameter values between two ***Models*** must be identical to constitute a "mesh match". If the Boolean for a parameter is false, then the parameter values between the two ***Models*** can be different and still constitute a "mesh match". The process of identifying "mesh matches" is automated and is only performed if the "recycle_meshes" parameter in ***Run*** is true.
3. Syntax: To declare this entity in config/system/mesh_dependent_model.json, use the following syntax:
    - The same key-value structure pair as in ***Model***, but the values are of type Boolean
        - true: The parameter values between the two ***Model*** configurations must be identical to constitute a "mesh match".

- false: The parameter values between the two ***Model*** configurations can be different and still constitute a “mesh match”.

4. Properties:

See: model.json

5. Example:

See: config/system/mesh_dependent_model.json

- Note: The user should not need to change this file unless adding new parameters to ***Model*** for expanded/modified pipeline functionality.

1.12 References

1. Weerasuriya A, Spangler RA, Rapoport SI, Taylor RE. AC impedance of the perineurium of the frog sciatic nerve. Biophys J. 1984 Aug;46(2):167–74. Available from: https://doi.org/10.1016/s0006-3495(84)84009-6 PMID: 6332648
2. Virtanen P, Gommers R, Oliphant TE, Haberland M, Reddy T, Cournapeau D, et al. SciPy 1.0: fundamental algorithms for scientific computing in Python. Nat Methods [Internet]. 2020;17(3):261–72. Available from: https://doi.org/10.1038/s41592-019-0686-2
