# Supplementary material for: ASCENT (Automated Simulations to Characterize Electrical Nerve Thresholds): A pipeline for sample-specific computational modeling of electrical stimulation of peripheral nerves
Source: PLoS Comput Biol. 2021 Sep 7;17(9):e1009285. doi: 10.1371/journal.pcbi.1009285 (PMC8423288; doi:10.1371/journal.pcbi.1009285)
Supplement: S9 Text — Python utility classes. (PDF) [file pcbi.1009285.s009.pdf]

1 S9 Text

Appendix. Python utility classes

1.1 Configurable

Configurable is inherited by other Python classes in the ASCENT pipeline to grant access to parameter and data configuration JSON files loaded to memory. Configurable has built-in exceptions that it throws which are indexed negatively (-1 and below by convention) because it is intrinsically unable to inherit from Exceptionable (errors indexed +1 and above by convention), which, in turn, (Exceptionable) is configured by inheriting the Configurable class.

Configurable is an important class for developers to understand because it is the mechanism by which instances of our Python classes inherit their properties from JSON configuration files (e.g., sample.json, model.json, sim.json, fiber_z.json). The Configurable class takes three input parameters:

1.1.1 SetupMode (from Enums, S6 Text):

Either NEW or OLD which determines if Configurable loads a new JSON (from file) or uses data that has already been created in Python memory as a dictionary or list, respectively.

1.1.2 ConfigKey (from Enums, S6 Text):

The ConfigKey indicates the choice of configuration data type and is also the name of the configuration JSON file (e.g., sample.json, model.json, sim.json, run.json, env.json).

1.1.3 Config:

The Config input to Configurable can take one of three data types. If “SetupMode” is “OLD”, the value can be a dictionary or list of already loaded configuration data. If “SetupMode” is “NEW”, the value must be a string of the file path to the configuration file to be loaded into memory.

1.1.4 Example use of Configurable:

When the Sample class is instantiated in Runner, it inherits functionality from Configurable (see Sample constructor __init__(self, exception_config: list) in src/core/sample.py).

After the Sample class is instantiated, the ***Sample*** configuration (index indicated in ***Run***) is added to the Sample class with:

sample.add(SetupMode.OLD, Config.SAMPLE, all_configs[Config.SAMPLE.value][0])

With the ***Sample*** configuration available to the Sample class, the class can access the contents of the JSON dictionary. For example, in populate(), the Sample class gets the length of the scale bar from ***Sample*** with the following line:

self.search(Config.SAMPLE, ‘scale’, ‘scale_bar_length’)

1.2 Exceptionable

Exceptionable is a centralized way to organize and throw exceptions (errors) to the user’s console. Exceptionable inherits functionality from Configurable. Exceptionable, like Configurable, is initialized with “SetupMode”, ConfigKey, and a Config. However, the data contents for Exceptionable are specifically a list of exceptions stored in config/system/exceptions.json. The contents of the exceptions configuration file is a list of numbered errors with an associated text description and error location that are printed to the console in the event of a raised exception.

1.3 Saveable

Saveable is a simple Python class that, when inherited by a Python class (e.g., Sample and Simulation, described in S13 and S30 Text, respectively), enables the class to save itself using Saveable’s save() method. Using pickle.dump(), the object is saved as a Python object to file at the location of the destination path, which is an input parameter to save().
