# Supplementary material for: ASCENT (Automated Simulations to Characterize Electrical Nerve Thresholds): A pipeline for sample-specific computational modeling of electrical stimulation of peripheral nerves
Source: PLoS Comput Biol. 2021 Sep 7;17(9):e1009285. doi: 10.1371/journal.pcbi.1009285 (PMC8423288; doi:10.1371/journal.pcbi.1009285)
Supplement: S10 Text — Submitting NEURON jobs. (PDF) [file pcbi.1009285.s010.pdf]

# 1 S10 Text

# Appendix. Submitting NEURON jobs

We provide scripts for the user to submit NEURON jobs in src/neuron/. It is suggested that the script ending in ".sh" is used for Linux-based cluster contexts; on the other hand, if running locally, the environment setup will likely be more specific to the machine, and the script ending in ".py" is the better choice. The submit.sh acts as a buffer for submit.py to set up the environment and load appropriate modules for job submission. The submit.py script takes the input of the ***Run*** configuration and submits a NEURON call for each independent fiber within an n_sim/. These scripts are called using similar syntax as pipeline.py: "./submit.<ext> <run indices>," where <run indices> is a space-separated list of integers. Note that these submission scripts expect to be called from a directory with the structure generated by Simulation.export_nsims() at the location defined by "ASCENT_NSIM_EXPORT_PATH" in env.json.
