# Supplementary material for: ASCENT (Automated Simulations to Characterize Electrical Nerve Thresholds): A pipeline for sample-specific computational modeling of electrical stimulation of peripheral nerves
Source: PLoS Comput Biol. 2021 Sep 7;17(9):e1009285. doi: 10.1371/journal.pcbi.1009285 (PMC8423288; doi:10.1371/journal.pcbi.1009285)
Supplement: S11 Text — Morphology files. (PDF) [file pcbi.1009285.s011.pdf]

1 S11 Text

Appendix. Morphology files

Each mask must be binary (i.e., white pixels (‘1’) for the segmented tissue and black pixels (‘0’) elsewhere) and must use Tagged Image File Format (i.e., .tif, or .tiff). All masks must be defined within the same field of view, be the same size, and be the same resolution. To convert between pixels of the input masks to dimensioned length (micrometers), a mask for the scale bar (s.tif) of known length (oriented horizontally) must be provided (see “Scale Bar” in Fig 2) and the length of the scale bar must be indicated in ***Sample*** (S7 and S8 Text).

The user is required to set the “MaskInputMode” in ***Sample*** (“mask_input”) to communicate the contents of the segmented histology files (S7 and S8 Text). Ideally, segmented images of boundaries for both the “outers” (o.tif) and “inners” (i.tif) of the perineurium will be provided, either as two separate files (o.tif and i.tif) or combined in the same image (c.tif) (see “Inners”, “Outers”, and “Combined” in Fig 2). However, if only inners are provided—which identify the outer edge of the endoneurium—a surrounding perineurium thickness is defined by the “PerineuriumThicknessMode” in ***Sample*** (“ci_perineurium_thickness”); the thickness is user-defined, relating perineurium thickness to features of the inners (e.g., their diameter). It should be noted that defining nerve morphology with only inners does not allow the model to represent accurately fascicles containing multiple endoneurium inners within a single outer perineurium boundary (“peanut” fascicles; see an example in Fig 2); in this case, each inner trace will be assumed to represent a single independent fascicle that does not share its perineurium with any other inners; more accurate representation requires segmentation of the “outers” as well.

The user is required to set the “NerveMode” in ***Sample*** (“nerve”) to communicate the contents of the segmented histology files (S7 and S8 Text). The outer nerve boundary, if present, is defined with a separate mask (n.tif). In the case of a compound nerve with epineurium, the pipeline expects the outer boundary of the epineurium to be provided as the “nerve”. In the case of a nerve with a single fascicle, no nerve mask is required—in which case either the outer perineurium boundary (if present) or the inner perineurium boundary (otherwise) is used as the nerve boundary—although one may be provided if epineurium or other tissue that would be within the cuff is present in the sample histology.

Lastly, an “orientation” mask (a.tif) can be optionally defined. This mask should be all black except for a small portion that is white, representing the position to which the cuff must be rotated. The angle is measured *relative to the centroid of the nerve/singular fascicle*, so this image should be constructed while referencing n.tif (or, if monofascicular, i.tif, o.tif, or c.tif). By default, the 0º position of our cuffs correspond with the coordinate halfway along the arc length of the cuff inner diameter while the circular portion of a cuff’s diameter is centered at the origin (“angle_to_contacts_deg” in a “preset” cuff’s JSON file, and S17 and S19 Text). If a.tif is provided, other cuff rotation methods (“cuff_shift” in ***Model***, which calculate “pos_ang”) are overridden.

The user must provide segmented image morphology files, either from histology or the mock_morphology_generator.py script, with a specific naming convention in the input/ directory.

- Raw RGB image, to be available for convenience and used for data visualization: r.tif (optional).
- Combined (i.e., inners and outers): c.tif.
- Inners: i.tif
  - An “inner” is the internal boundary of the perineurium that forms the boundary between the perineurium and the endoneurium.
- Outers: o.tif
  - An “outer” is the external boundary of the perineurium that forms the boundary between the perineurium and the epineurium or extraneural medium.
- Scale bar: s.tif (scale bar oriented horizontally, required).
- Nerve: n.tif (optional for monofascicular nerves).
- Orientation: a.tif (optional).

For an example of input files, see Fig 2. The user must properly set the “MaskInputMode” in ***Sample*** (“mask_input”) for their provided segmented image morphology files (S7 and S8 Text).
