# Supplementary material for: ASCENT (Automated Simulations to Characterize Electrical Nerve Thresholds): A pipeline for sample-specific computational modeling of electrical stimulation of peripheral nerves
Source: PLoS Comput Biol. 2021 Sep 7;17(9):e1009285. doi: 10.1371/journal.pcbi.1009285 (PMC8423288; doi:10.1371/journal.pcbi.1009285)
Supplement: S12 Text — Python MockSample class for creating binary masks of nerve morphology. (PDF) [file pcbi.1009285.s012.pdf]

1 S12 Text

Appendix. Python MockSample class for creating binary masks of nerve morphology

MockSample is a Python class that manages the *data* and contains all operations to create binary masks of mock nerve morphology (i.e., nerve: n.tif, inners: i.tif, and scale bar: s.tif) to use as inputs to the pipeline.

The user defines the parameter values in config/user/mock_samples/<mock_sample_index>.json (with a template provided in config/templates/mock_sample.json). The mock sample morphology is then created using the JSON file by executing “python run mock_morphology_generator <mock_sample_index>” at the project root. The mock morphology generator uses the MockSample Python class to create binary images of the nerve, inner perineurium traces (fascicles), and the scale bar in input/<NAME>/ (NAME is analogous to the “sample” parameter in ***Sample***, following the standard naming convention (S7 and S8 Text)), which allow the pipeline to function as if binary images of segmented histology were provided. The <mock_sample_index>.json file and the resulting segmented nerve morphology files are automatically saved in input/<NAME>/.

MockSample is Exceptionable, Configurable, and has instance attributes of “nerve” and a list “fascicles”. After the MockSample class is initialized, a mock_sample.json file is added to the class instance. The mock_morphology_generator.py script configures an instance of the MockSample class using the first input argument, which references the index of a JSON file stored in config/user/mock_samples/.

In mock_morphology_generator.py, MockSample’s methods make_nerve() and make_fascicles() are called to create ellipses for the nerve and fascicles in memory based on the parameters in the mock_sample.json file. MockSample’s methods ensure that the fascicles have a minimum distance between each fascicle boundary and the nerve and between fascicle boundaries. For details on the parameters that define sample morphology using our mock nerve morphology generator, see S7 Text for a description of mock_sample.json and S8 Text for details of the syntax/data type of the key-value parameter pairs required to define a mock sample. Lastly, MockSample’s make_masks() method is called on the class instance to create binary masks and save them as TIFs in the input/<NAME>/ directory.
