# Supplementary material for: ASCENT (Automated Simulations to Characterize Electrical Nerve Thresholds): A pipeline for sample-specific computational modeling of electrical stimulation of peripheral nerves
Source: PLoS Comput Biol. 2021 Sep 7;17(9):e1009285. doi: 10.1371/journal.pcbi.1009285 (PMC8423288; doi:10.1371/journal.pcbi.1009285)
Supplement: S13 Text — Python classes for representing nerve morphology (Sample). (PDF) [file pcbi.1009285.s013.pdf]

# 1 S13 Text

# Appendix. Python classes for representing nerve morphology (Sample)

The nerve cross section includes the outer nerve trace (if present; not required for monofascicular nerves) and, for each fascicle, either a single “inner” perineurium trace or both “inner” and “outer” perineurium traces. We provide automated control to correct for tissue shrinkage during histological processes [1] (S8 Text). Morphology metrics (e.g., nerve and fascicle(s) cross-sectional areas and centroids, major and minor axis lengths, and rotations of the best-fit ellipses) are automatically reported in ***Sample*** (S8 Text).

## 1.1 Trace

Trace is the core Python class for handling a list of points that define a closed loop for a tissue boundary in a nerve cross section (see “Tissue Boundaries” in Fig 2). Trace has built-in functionality for transforming, reporting, displaying, saving, and performing calculations on its data contents and properties. Python classes Nerve, Fascicle, and Slide are all special instances or hierarchical collections of Trace.

A Trace requires inputs of a set of (x,y)-points that define a closed loop and an exceptions JSON configuration file. The z-points are assumed to be ‘0’. The Trace class already provides many built-in functionalities, but any further user-desired methods needed either to mutate or access nerve morphology should be added to the Trace class.

Trace uses the *OpenCV* [2], *Pyclipper* [3], and *Shapely* [4] Python packages to support modifier methods (e.g., for performing transformations):

- scale(): Used to assign dimensional units to points and to correct for shrinkage of nerve tissues during processing of histology.
- rotate(): Performs a rigid rotational transformation of Trace about a point (positive angles are counter-clockwise and negative are clockwise).
- shift(): performs a 2D translational shift to Trace (in the (x,y)-plane, i.e., the sample cross section).
- offset(): Offsets Trace’s boundary by a discrete distance from the existing Trace boundary (non-affine transformation in the (x,y)-plane, i.e., the sample cross section).
- pymunk_poly(): Uses *Pymunk* to create a body with mass and inertia for a given Trace boundary (used in deform(), the fascicle repositioning method, from the Deformable class).
- pymunk_segments(): Uses *Pymunk* to create a static body for representing intermediate nerve boundaries (used in deform(), the fascicle repositioning method, from the Deformable class).

Trace also contains accessor methods:

- within(): Returns a Boolean indicating if a Trace is completely within another Trace.
- intersects(): Returns a Boolean indicating if a Trace is intersecting another Trace.
- centroid(): Returns the centroid of the best fit ellipse of Trace.
- area(): Returns the cross-sectional area of Trace.
- random_points(): Returns a random list of coordinates within the Trace (used to define axon locations within the Trace).

Lastly, Trace has a few utility methods:

- plot(): Plots the Trace using formatting options (i.e., using the plt.plot format, see Matplotlib documentation (https://matplotlib.org/contents.html) for details).
- deepcopy(): Fully copy an instance of Trace (i.e., copy data, not just a reference/pointer to original instance).
- write(): Writes the Trace data to the provided file format (currently, only COMSOL's sectionwise format (https://www.comsol.com/fileformats—ASCII with .txt extension containing column vectors for x- and y-coordinates—is supported).

## 1.2 Nerve

Nerve is the name of a special instance of Trace reserved for representing the outer nerve (epineurium) boundary. It functions as an alias for Trace. An instance of the Nerve class is created if the "NerveMode" in ***Sample*** ("nerve") is "PRESENT" (S8 Text).

## 1.3 Fascicle

Fascicle is a class that bundles together instance(s) of Trace to represent a single fascicle in a slide. Fascicle can be defined with either (1) an instance of Trace representing an outer perineurium trace and one or more instances of Trace representing inner perineurium traces, or (2) an inner perineurium trace that is subsequently scaled to make a virtual outer using Trace's methods deepcopy() and offset() and the perineurium thickness defined by the "PerineuriumThicknessMode" in ***Sample*** ("ci_perineurium_thickness") (S8 Text). Upon instantiation, Fascicle automatically validates that each inner instance of Trace is fully within its outer instance of Trace and that no inner instance of Trace intersects another inner instance of Trace.

Fascicle contains methods for converting a binary mask image of segmented fascicles into instances of the Fascicle class. The method used depends on the contents of the binary image inputs to the pipeline as indicated by the "MaskInputMode" in ***Sample*** ("mask_input") (i.e., INNER_AND_OUTER_SEPARATE, INNER_AND_OUTER_COMPILED, or INNERS). For each of the mask-to-Fascicle conversion methods, the OpenCV Python package finds material boundaries and reports their nested hierarchy (i.e., which inner Traces are within which outer Traces, thereby associating each outer with one or more inners). The methods are expecting a *maximum* hierarchical level of 2: one level for inners and one level for outers.

- If separate binary images were provided containing contours for inners (i.tif) and outers (o.tif), then the “MaskInputMode” in ***Sample*** (“mask_input”, S8 Text) is INNER_AND_OUTER_SEPARATE; in this case, the Fascicle class uses its separate_to_list() method.
- If a single binary image was provided containing combined contours of inners and outers (c.tif), then the “MaskInputMode” in ***Sample*** (“mask_input”, S8 Text) is INNER_AND_OUTER_COMPILED; in this case, the Fascicle class uses its compiled_to_list() method.
- If only a binary image was provided for contours of inners (i.tif), the “MaskInputMode” (“mask_input”, S8 Text) in ***Sample*** is INNERS; in this case, Fascicle class uses its inner_to_list() method.

Additionally, Fascicle has a write() method which saves a Fascicle’s inner (one or many) and outer Traces to files that later serve as inputs for COMSOL to define material boundaries in a nerve cross section (sectionwise file format (https://www.comsol.com/fileformats), i.e., ASCII with .txt extension containing column vectors for x- and y-coordinates). Lastly, Fascicle has a morphology_data() method which uses Trace’s area() and ellipse() methods to return the area and the best-fit ellipse centroid, axes, and rotation of each outer and inner as a JSON Object to ***Sample*** (S8 Text).

## 1.4 Slide

The Slide class represents the morphology of a single transverse cross section of a nerve sample (i.e., nerve and fascicle boundaries). An important convention of the pipeline is that the nerve is always translated such that its centroid (i.e., from best-fit ellipse) is at the origin (x,y,z) = (0,0,0) and then extruded in the positive (z)-direction in COMSOL. Slide allows operations such as translation and plotting to be performed on all Nerve and Fascicle Traces that define a sample collectively.

To create an instance of the Slide class, the following items must be defined:

- A list of instance(s) of the Fascicle class.
- “NerveMode” from ***Sample*** (“nerve”) (i.e., PRESENT as in the case of nerves with epineurium (n.tif) or NOT_PRESENT otherwise (S8 Text)).
- An instance of the Nerve class if “NerveMode” is PRESENT.
- A Boolean for whether to reposition fascicles within the nerve from “ReshapeNerveMode” in ***Sample*** (S8 Text).
- A list of exceptions.

The Slide class validates, manipulates, and writes its contents.

- In Slide’s validation() method, Slide returns a Boolean indicating if its Fascicles and Nerve Traces are overlapping or too close to one another (based on the minimum fascicle separation parameter in ***Sample***).

- In Slide’s move_center() method, Slide repositions its contents about a central coordinate using Trace’s shift() method available to both the Nerve and Fascicle classes (by convention, in ASCENT this is (x,y) = (0,0)).
- In Slide’s reshaped_nerve() method, Slide returns the deformed boundary of Nerve based on the “ReshapeNerveMode” in ***Sample*** (“reshape_nerve”, S8 Text) (e.g., CIRCLE).
- Using the methods of Nerve and Fascicle, which are both manifestations of Trace, Slide has its own methods plot(), scale(), and rotate().
- Slide has its own write() method which determines the file structure to which the Trace contours are saved to file in samples/<sample index>/slides/.

Note that the sample data hierarchy can contain more than a single Slide instance (the default being 0 as the cassette index and 0 as the section index, hence the 0/0 seen in S3 Text Figure A), even though the pipeline data processing assumes that only a single Slide exists. This will allow the current data hierarchy to be backwards compatible if multi-Slide samples are processed in the future.

## 1.5 Map

Map is a Python class used to keep track of the relationship of the longitudinal position of all Slide instances for a Sample class. At present, the pipeline only supports models of nerves with constant cross-sectional area, meaning only one Slide is used per FEM, but this class is implemented for future expansion of the pipeline to construct three-dimensional nerve models with varying cross section (e.g., using serial histological sections). If only one slide is provided, Map is generated automatically, and the user should have little need to interact with this class.

## 1.6 Sample

The Sample class is initialized within Runner’s run() method by loading ***Sample*** and ***Run*** configurations (S7 and S8 Text). First, Sample’s build_file_structure() method creates directories in samples/ and populates them with the user’s file inputs from input/<NAME>/; the images are copied over for subsequent processing, as well as for convenience in creating summary figures. Sample then uses its populate() method to construct instances of Nerve and Fascicle in memory from the input sample morphology binary images (see Fascicle class above for details). Sample’s populate() method packages instances of Nerve and Fascicle(s) into an instance of Slide.

Sample’s scale() method is used to convert Trace points from pixel coordinates to coordinates with units of distance based on the length of the horizontal scale bar as defined in ***Sample*** (micrometers) and the width of the scale bar (pixels) in the input binary image (s.tif) (S8 Text). The scale bar needs to be a perfectly horizontal line. Sample’s scale() method is also used within populate() to correct for shrinkage that may have occurred during the histological tissue processing. The percentage increase for shrinkage correction in the slide's 2D geometry is stored as a parameter “shrinkage” in ***Sample*** (S8 Text). Additionally, Slide has a move_center() method which is used to center Slide about a point within populate(). Note that Sample is centered with the centroid of the best-fit ellipse of the outermost Trace (Nerve if “NerveMode” in ***Sample***

(“nerve”) is “PRESENT”, outer Trace if “NerveMode” is “NOT_PRESENT” (S8 Text)) at the origin (0,0,0). Change in rotational or translational placement of the cuff around the nerve is accomplished by moving the cuff and keeping the nerve position fixed (S19 Text).

Sample’s populate() method also manages operations for saving tissue boundaries of the Sample (Nerve and Fascicles) to CAD files (slides/#/#/sectionwise2d/) for input to COMSOL with Sample’s write() method.

Sample’s output_morphology_data() method collects sample morphology information (area, and the best-fit ellipse information: centroid, major axis, minor axis, and rotation) for each original Trace (i.e., Fascicle inners and outers, and Nerve) and saves the data under “Morphology” in ***Sample*** (S8 Text).

Lastly, since Sample inherits Saveable, Sample has access to the save() method which saves the Python object to file.

## 1.7 Deformable

If “DeformationMode” in ***Sample*** (“deform”) is set to NONE, then the Deformable class takes no action (S8 Text). However, if “DeformationMode” in ***Sample*** is set to PHYSICS, then Deformable’s deform() method simulates the change in nerve cross section that occurs when a nerve is placed in a cuff electrode. Specifically, the outer boundary of a Slide’s Nerve mask is transformed into a user-defined final geometry based on the “ReshapeNerveMode” in ***Sample*** (i.e., CIRCLE) while maintaining the cross-sectional area. Meanwhile, the fascicles (i.e., outers) are repositioned within the new nerve cross section in a physics-based way using Pymunk [5], a 2D physics library, in which each fascicle is treated as rigid body with no elasticity as it is slowly “pushed” into place by both surrounding fascicles and the nerve boundary (Figure A).

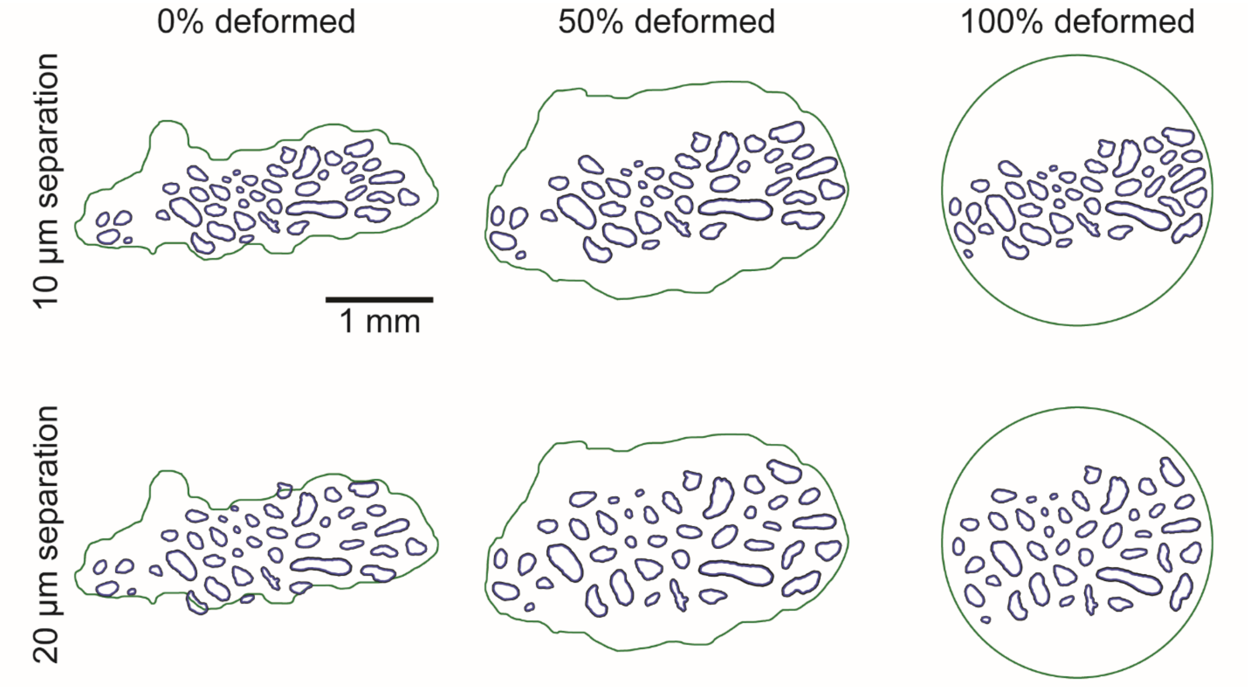


Figure A. Snapshots at 0%, 50%, and 100% (left-to-right) of the deformation process powered by the pygame package [6]. The deformation process is shown for two minimum fascicle separation constraints: 10 µm (top row) and 20 µm (bottom row). The geometry at 0% deformation is shown after the fascicles have been spread out to the minimum separation constraint.

The deform() method updates the nerve boundary to intermediately-deformed nerve traces between the nerve's "boundary_start" (i.e., the Trace's profile in segmented image) and "boundary_end" (i.e., the Trace's profile after accommodation to the cuff's inner diameter, which is determined by the "ReshapeNerveMode" ("reshape_nerve", S8 Text) while the fascicle contents are allowed to rearrange in a physics-space. By default, all fascicles have the same "mass", but their moment of inertia is calculated for each fascicle based on its geometry (see Trace's pymunk_poly() method). Each fascicle is also assigned a "friction coefficient" of 0.5 as well as a "density" of 0.01. These measurements are mostly important as they relate to one another, not as absolute values. Importantly, we set the elasticity of all the fascicles to 0, so all kinetic energy is absorbed, and fascicles only move if they are directly pushed by another fascicle or by the nerve barrier. In Sample's populate() method, the final fascicle locations and rotations returned by the deform() method are then applied to each fascicle using the Fascicle class's shift() and rotate() methods.

Deformable's convenience constructor, from_slide(), is automatically called in Sample's populate() method, where a Slide is deformed to user specification. The from_slide() method takes three input arguments: The Slide object from which to construct the current Deformable object, the "ReshapeNerveMode" (e.g., CIRCLE, S8 Text), and the minimum distance between fascicles. If only inners are provided, virtual outers interact during nerve deformation to account for the thickness of the perineurium. Each inner's perineurium thickness is defined by the "PerineuriumThicknessMode" in ***Sample*** ("ci_perineurium_thickness", S8 Text), which specifies the linear relationship between inner diameter and perineurium thickness defined in

config/system/ci_peri_thickness.json (S8 Text). Deformable's from_slide() method uses Deformable's deform_steps() method to calculate the intermediately-deformed nerve traces between the boundary_start and the boundary_end, which contain the same number of points and maintain nerve cross-sectional area. The deform_steps() method maps points between boundary_start and boundary_end in the following manner. Starting from the two points where the major axis of the Nerve's best-fit ellipse intersects boundary_start and boundary_end, the algorithm matches consecutive boundary_start and boundary_end points and calculates the vectors between all point pairs. The deform_steps() method then returns a list of intermediately-deformed nerve traces between the boundary_start and boundary_end by adding linearly-spaced portions of each point pair's vector to boundary_start. Also note that by defining "deform_ratio" (value between 0 and 1) in ***Sample***, the user can optionally indicate a partial deformation of the Nerve (S8 Text).

Enforcing a minimum fascicle separation that is extremely large (e.g., 20 µm) can cause inaccurate deformation, as fascicles may be unable to satisfy both minimum separation constraints and nerve boundary constraints.

To maintain a minimum distance between adjacent fascicles, the Deformable's deform() method uses Trace's offset() method to perform a non-affine scaling out of the fascicle boundaries by a fixed distance before defining the fascicles as rigid bodies in the pygame physics space. At regular intervals in physics simulation time, the nerve boundary is updated to the next Trace in the list of intermediately-deformed nerve traces created by deform_steps(). This number of Trace steps defaults to 36 but can be optionally set in ***Sample*** with the "morph_count" parameter by the user (S8 Text). It is important to note that too few Trace steps can result in fascicles lying outside of the nerve during deformation, while too many Trace steps can be unnecessarily time intensive. We've set the default to 36 because it tends to minimize both aforementioned issues for all sample sizes and types that we have tested.

The user may also visualize nerve deformation by setting the "deform_animate" argument to true in sample.populate() (called in Runner's run() method) (S8 Text). Visualizing sample deformation can be helpful for debugging but increases computational load and slows down the deformation process significantly. When performing deformation on many slides, we advise setting this flag to false.

## 1.8 References


1. Boyd IA, Kalu KU. Scaling factor relating conduction velocity and diameter for myelinated afferent nerve fibres in the cat hind limb. J Physiol. 1979 Apr;289:277–97. Available from: https://doi.org/10.1113/jphysiol.1979.sp012737 PMID: 458657
2. Bradski G, Daebler A. Learning OpenCV. Computer vision with OpenCV library. 2008 Jan 1;222–64.
3. Johnson A, Chalton M, Treyer L, Ratajc G. pyclipper · PyPI [Internet]. 2019 [cited 2020 Apr 20]. Available from: https://pypi.org/project/pyclipper/
4. Gillies S. Shapely · PyPI [Internet]. 2019 [cited 2020 Apr 20]. Available from: https://pypi.org/project/Shapely/

5. Blomqvist V. pymunk · PyPI [Internet]. 2019 [cited 2020 Apr 20]. Available from: https://pypi.org/project/pymunk/
6. Shinners P. Pygame Intro — pygame v2.0.0.dev5 documentation [Internet]. [cited 2020 Apr 20]. Available from: https://www.pygame.org/docs/tut/PygameIntro.html
