# Supplementary material for: ASCENT (Automated Simulations to Characterize Electrical Nerve Thresholds): A pipeline for sample-specific computational modeling of electrical stimulation of peripheral nerves
Source: PLoS Comput Biol. 2021 Sep 7;17(9):e1009285. doi: 10.1371/journal.pcbi.1009285 (PMC8423288; doi:10.1371/journal.pcbi.1009285)
Supplement: S14 Text — Creating sample-specific nerve morphologies in COMSOL. (PDF) [file pcbi.1009285.s014.pdf]

1 S14 Text

Appendix. Creating sample-specific nerve morphologies in COMSOL

1.1 ModelWrapper.addNerve()

The addNerve() method adds the nerve components to the COMSOL “model” object using Java. If the “NerveMode” in ***Sample*** (“nerve”) is “PRESENT” (S8 Text) the program creates a part instance of epineurium using the createNervePartInstance() method in Part (src/model/Part.java). The addNerve() method then searches through all directories in fascicles/ for the sample being modeled, and, for each fascicle, assigns a path for the inner(s) and outer in a HashMap. The HashMap of fascicle directories is then passed to the createNervePartInstance() method in Part which adds fascicles to the COMSOL “model” object.

1.1.1 Part.createNervePartInstance()

The createNervePartInstance() method in Part (src/model/Part.java) creates three-dimensional representations of the nerve sample including its endoneurium, perineurium, and epineurium. The createNervePartInstance() method defines domain and surface geometries and contributes them to COMSOL selections (lists of indices for domains, surfaces, boundaries, or points), which are necessary to later assign physics properties.

1.1.1.1 Fascicles

ASCENT uses CAD sectionwise (https://www.comsol.com/fileformats) files (i.e., ASCII with .txt extension containing column vectors for x- and y-coordinates) created by the Python Sample class to define fascicle tissue boundaries in COMSOL.

We provide the “use_ci” mode in ***Model*** (S8 Text) to model the perineurium using COMSOL’s contact impedance boundary condition for fascicles with only one inner (i.e., endoneurium) domain for each outer (i.e., perineurium) domain (S28 Text). If “use_ci” mode is true, the perineurium for all fascicles with exactly one inner and one outer is represented with a contact impedance. The pipeline does not support control of using the contact impedance boundary condition on a fascicle-by-fascicle basis.

The createNervePartInstance() method in Part (src/model/Part.java) performs a directory dive on the output CAD files samples/<sample_index>/slides/<#>/<#>/sectionwise2d/fascicles/<outer, inners>/ from the Sample class in Python to create a fascicle for each outer. Depending on the number of corresponding inners for each outer saved in the file structure and the “use_ci” mode in ***Model***, the program either represents the perineurium in COMSOL as a surface with contact

impedance (FascicleCI: Fascicles with one inner per outer and if “use_ci” mode is true) or with a three-dimensional meshed domain (FascicleMesh: Fascicles with multiple inners per outer or if “use_ci” parameter is false).

1.1.1.2 Epineurium

The createNervePartInstance() method in Part (src/model/Part.java) contains the operations required to represent epineurium in COMSOL. The epineurium cross section is represented as a circle/ellipse from the input epineurium boundary and is then extruded into the third dimension. This is only performed if the “NerveMode” (i.e., “nerve”) in ***Sample*** is “PRESENT” and n.tif is provided (S8 Text).
