# Supplementary material for: ASCENT (Automated Simulations to Characterize Electrical Nerve Thresholds): A pipeline for sample-specific computational modeling of electrical stimulation of peripheral nerves
Source: PLoS Comput Biol. 2021 Sep 7;17(9):e1009285. doi: 10.1371/journal.pcbi.1009285 (PMC8423288; doi:10.1371/journal.pcbi.1009285)
Supplement: S15 Text — Micro-Leads cuff measurements. (PDF) [file pcbi.1009285.s015.pdf]

1 S15 Text

Appendix. Micro-Leads cuff measurements

We collected and measured images of 200, 300, and 400 µm Micro-Leads Neuro cuffs (Somerville, MA) (Figure A and Table A).

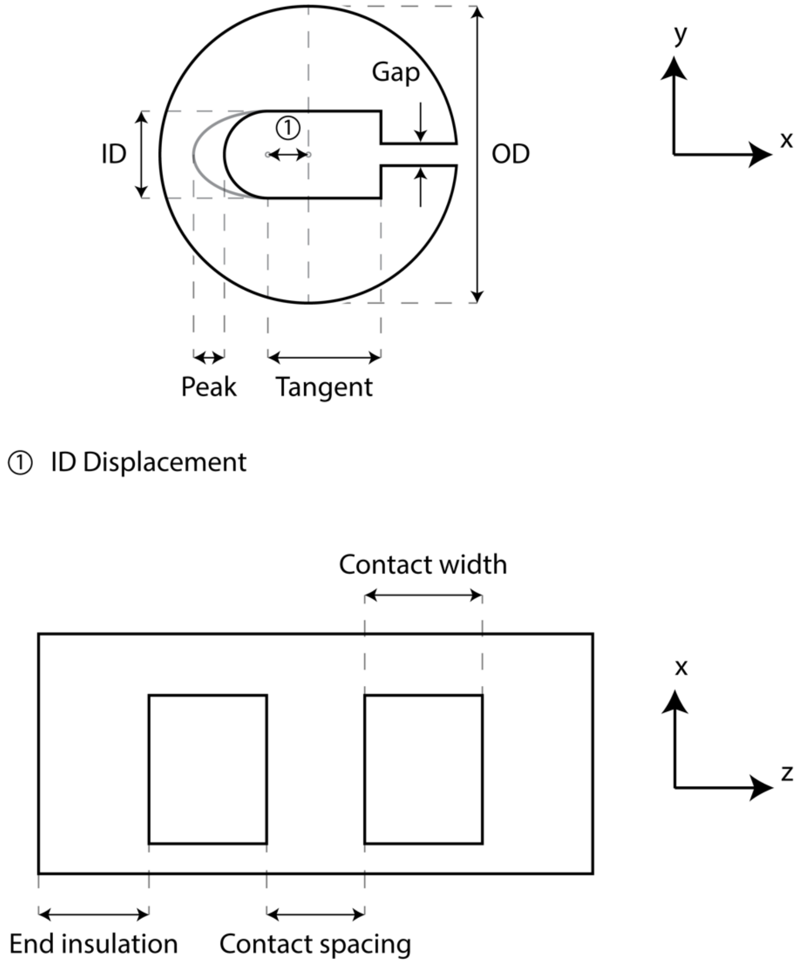

Figure A. Micro-Leads cuff measurements taken for 200, 300, and 400 µm inner diameter cuffs. Inner diameter (ID) displacement is defined from the center of the circle of the outer diameter (OD) to the center of the semi-circle for the inner diameter.

Table A. Cuff measurements (units: micrometer) for 200, 300, and 400 µm inner diameter Micro-Leads cuffs.

Cross-Sectional Measurements								Longitudinal Measurements		
Cuff	# cuffs measured	OD	ID	ID displacement (x)	Peak (x)	Tangent (x)	Gap (y)	End insulation	Contact width	Contact spacing
200 µm	6 bipolar	1080	210	135	15	175	30	295	590	305
300 µm	12 bipolar, 2 tripolar	1190	265	130	30	255	40	415	680	390
400 µm	4 bipolar	1605	465	325	0	200	35	535	745	475

We also collected and measured images of 100 µm Micro-Leads cuffs, which had a different cross section from the larger diameter cuffs and are therefore reported separately (Figure B and Table B).

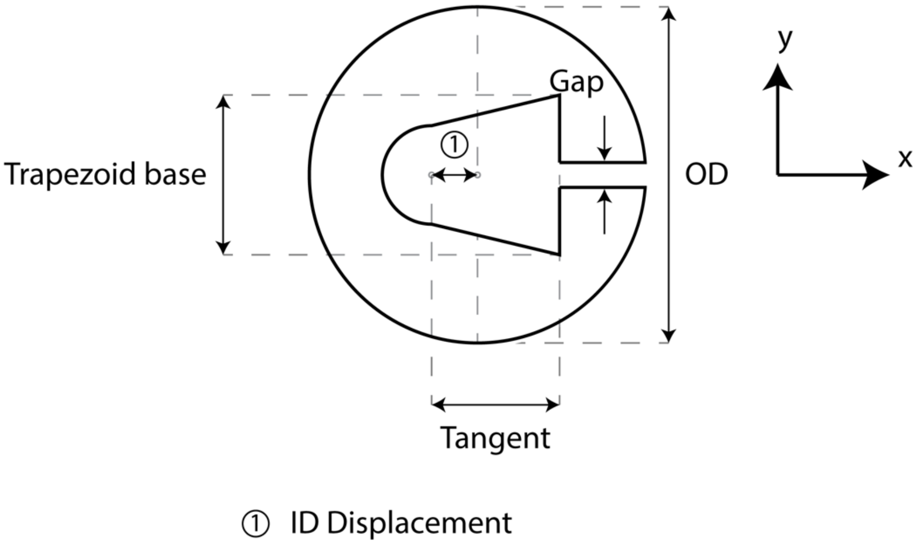

Figure B. Micro-Leads cuff measurements taken for 100 µm inner diameter cuffs. Inner diameter (ID) displacement is defined from the center of the circle of the outer diameter (OD) to the center of the semi-circle for the inner diameter.

Table B. Cuff measurements (units: micrometer) for 100 µm inner diameter Micro-Leads cuffs.

Cross Sectional Measurements								Longitudinal Measurements		
Cuff	# cuffs measured	OD	ID	Trapezoid base	Tangent (x)	Gap (y)	ID displacement (x)	End insulation	Contact width	Contact spacing
200 µm	6	1060	125	200	85	30	210	160	320	150

For all “preset” cuff JSON files based on our Micro-Leads cuff measurements, we recessed the contacts by 50 µm. Though we were not able to directly measure the recess depth of the contacts, Micro-Leads informed us that the surface of the electrode metal could never be recessed by more than ~60-70 µm.
