# Supplementary material for: ASCENT (Automated Simulations to Characterize Electrical Nerve Thresholds): A pipeline for sample-specific computational modeling of electrical stimulation of peripheral nerves
Source: PLoS Comput Biol. 2021 Sep 7;17(9):e1009285. doi: 10.1371/journal.pcbi.1009285 (PMC8423288; doi:10.1371/journal.pcbi.1009285)
Supplement: S16 Text — Library of part primitives for electrode contacts and cuffs. (PDF) [file pcbi.1009285.s016.pdf]

1 S16 Text

Appendix. Library of part primitives for electrode contacts and cuffs

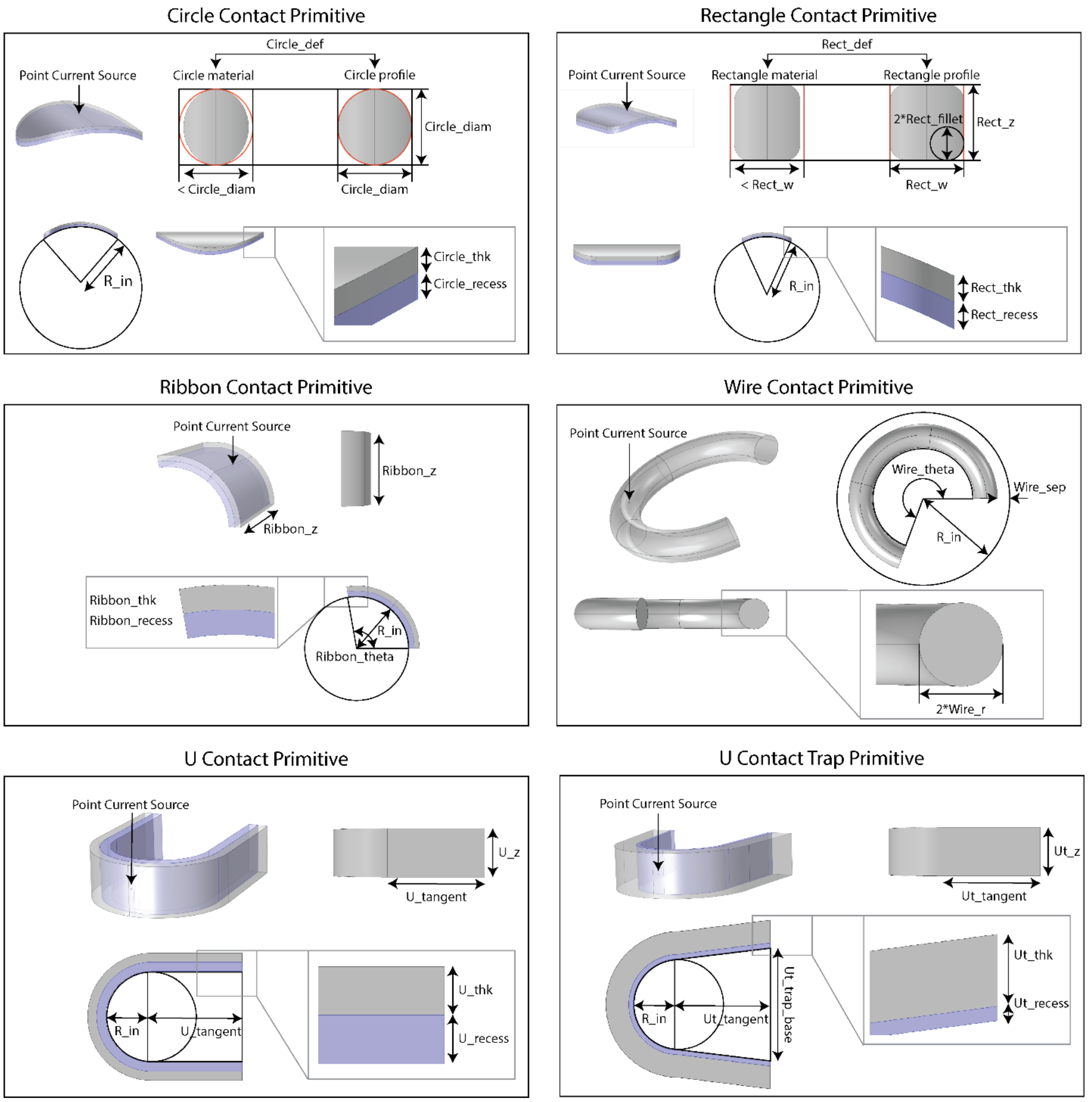

Figure A. Library of parts for electrode contacts. In the top two panels, “circle material” and “rectangle material” indicate that the contact’s dimensions refer to the length of the materials, rather than the dimension of the contact when it is bent to wrap around the circular inner diameter of the cuff. The dimensions “_thk” refer to the contact thickness. The dimensions “_recess” refer to the thickness of the domain created if the contact is recessed into the cuff.

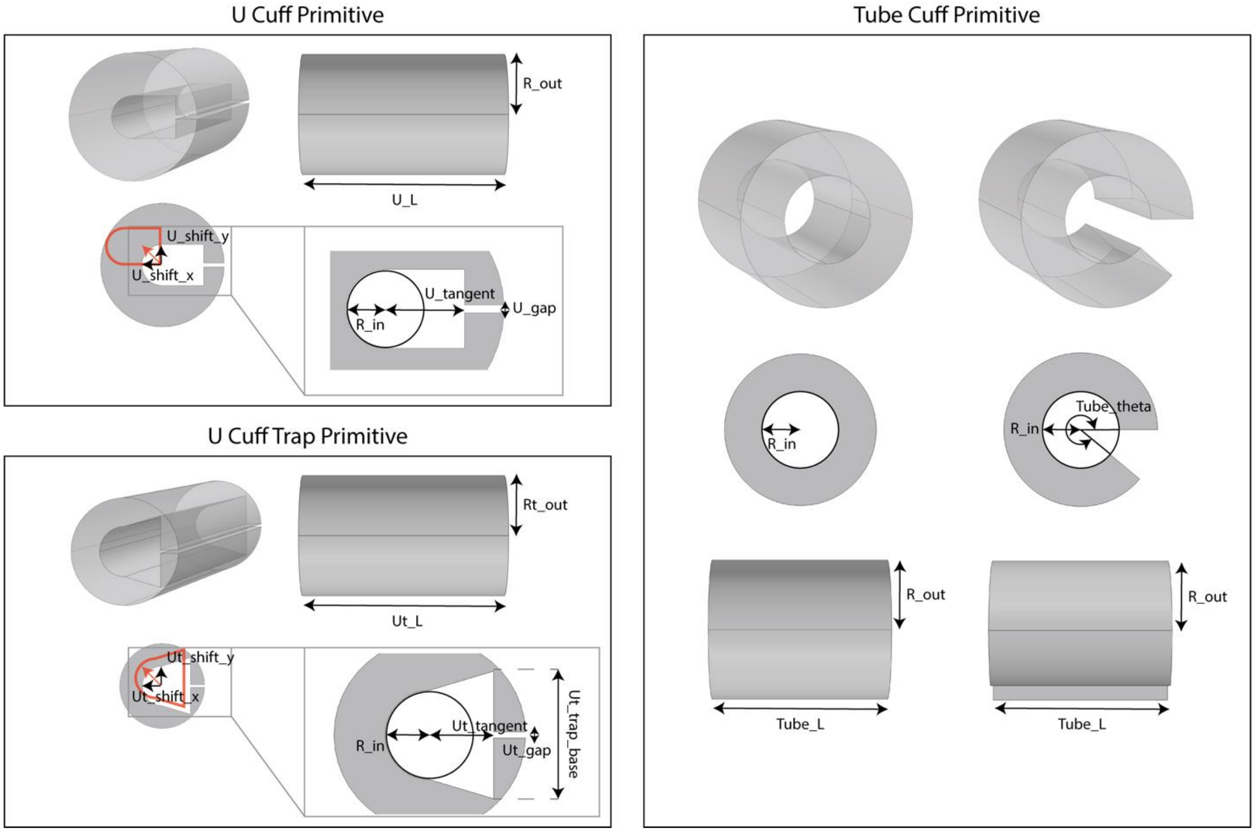

Figure B. Library of parts for cuffs. Parameters “U(t)_shift_x” and “U(t)_shift_y” change how the insulating material is centered around the inner diameter of the cuff as shown by the orange annotation.
