# Supplementary material for: ASCENT (Automated Simulations to Characterize Electrical Nerve Thresholds): A pipeline for sample-specific computational modeling of electrical stimulation of peripheral nerves
Source: PLoS Comput Biol. 2021 Sep 7;17(9):e1009285. doi: 10.1371/journal.pcbi.1009285 (PMC8423288; doi:10.1371/journal.pcbi.1009285)
Supplement: S17 Text — Creating custom preset cuffs from instances of part primitives. (PDF) [file pcbi.1009285.s017.pdf]

1 S17 Text

Appendix. Creating custom preset cuffs from instances of part primitives

The operations by which cuffs are added to the COMSOL "model" object are contained in the Java Part class (src/model/Part.java). A complete cuff design is defined by a JSON file (e.g., Purdue.json) stored in config/system/cuffs/, which we call a "preset" cuff, containing parameterizations and material assignments for part primitives that together represent an entire cuff electrode, including contact "recess" and "fill" (i.e., saline, mineral oil, or encapsulation tissue). The contents of the "preset" JSON file direct the Part class on which part primitives to add as well as their size, shape, placement, and material function (i.e., cuff "insulator", contact "conductor", contact "recess", and cuff "fill"). Fig 3A shows some examples of "preset" cuffs constructed from our library of COMSOL part primitives which are included in the pipeline repository in config/system/cuffs/. Users should not modify existing "preset" cuff files. Rather, a user should use our "preset" cuffs as a guide in constructing their own custom cuffs, which require a unique file name. Once a cuff is defined as a "preset" in config/system/cuffs/ as its own JSON file, the user may choose to place the cuff on a nerve in COMSOL using the "preset" parameter in ***Model*** (S8 Text).

We provide a COMSOL file in examples/parts/sandbox.mph that contains our library of "Geometry Parts" (i.e., part primitives) for users to assemble into their own cuffs in the COMSOL GUI. See S18 Text for instructions on how to add new part primitives. After a part primitive is defined in the "Geometry Parts" node in the COMSOL GUI, under "Component 1", the user may secondary-click on the "Geometry 1" node → "Parts" and select a part primitive. Importantly, the order of instantiated parts in the "Geometry 1" node matters for proper material assignment; the user must consider that when a new part occupies volume within a previously instantiated part, COMSOL will override the previous material assignment for the shared volume with the latter created part's material assignment. For example, if a user is modeling a cuff containing an embedded contact electrode (i.e., the contact surface is flush with the insulator's inner surface) and the entire cuff is bathed in saline (i.e., surgical pocket), the user would (1) add the part primitive for the saline cuff "fill" since it is the outer-most domain, (2) add the cuff "insulator" which would override its volume within the saline, and (3) add the contact "conductor" which would override the cuff insulator domain within the contact conductor. The order of instantiation of part primitives in COMSOL mirrors the order of the parts listed in the "preset" JSON file.

The part's required instantiation "Input Parameters" (in the "Settings" tab) have default values (found in the "Expression" dialogue boxes) that should be overridden and populated using parameter values to define the geometry of the part in your preset's implementation. The "Expression" dialogue boxes must contain parameter values already defined in a "Parameter Group" under the "Global Definitions" node (i.e., parameter names for either constants with units

(e.g., “5 [um]”) or mathematical relationships between other parameters (e.g., “parameter1 + parameter2”)). The parameter values in the “Parameter Group” under the “Global Definitions” node are populated with the list of “params” in the “preset” cuff JSON file (explained in more detail below).

Once the user has succeeded in assembling their cuff in the COMSOL GUI from parameterized instantiations of parts in examples/parts/sandbox.mph, they are ready to create a new “preset” cuff JSON file in config/system/cuffs/. See the existing files in that directory for examples. The required elements of a “preset” cuff JSON file are shown in the skeleton structure below:

```
{
  "code": String
  "instances": [
    {
      "type": String,
      "label": Double,
      "def": {
        "parameter1": String // key is name of expected parameter in COMSOL
        … // for all parameters (specific for part primitive “type”)
      },
      "materials": [
        {
          "info": String,
          "label_index": Integer
        }
        … // for all materials in a part
      ]
    }
    … // for all instances
   ]
   "params": [
     {
       "name": String, // e.g., “parameter1_<code_value>” … such as “pitch_Pitt”
       "expression": String,
       "description": String
     }
     … // for all parameters
   ]
  },
  "expandable": Boolean,
  "angle_to_contacts_deg": Double,
  "offset": {
    "parameter1": Double,
    // parameter must be defined in “params” list, value is weight of parameter value
```

```
    // (e.g., if radius of wire contact, need 2 to get contributing radial distance
    // between nerve and cuff)
    … // for all parameters informing how much "extra" space needed in cuff
  }
}
```

"code": The value (String) is a unique identifier for the parameters that are needed to define this cuff. All parameters in the "params" [Object, …] will need to end with the characters of this code preceded by "_" (e.g., "code" = "Pitt", the "pitch" parameter for the separation between contacts would be "pitch_Pitt").

"instances": The value is a list of JSON Objects, one for each part instance needed to represent the cuff. Within each part instance JSON Object, the user must define:

- "type": The value (String) defines which known primitive to instantiate, which matches the switch-case in BOTH Part.createCuffPartPrimitive() AND Part.createCuffPartInstance() in Java (src/model/Part.java) behind the scenes.
- "label": The value (String) defines the label that will show up in the COMSOL file to annotate the instance of the part primitive in the construction of your COMSOL FEM.
- "def": The value (Object) contains all parameters required to instantiate the chosen part primitive. The key-value pairs will match the values entered in the COMSOL GUI for a part (i.e., "Settings" -> "Input Parameters" panel) in examples/parts/sandbox.mph.
    - Key-value pairs in this JSON Object will vary depending on the part primitive as defined in "type". For each parameter key, the value is a String containing a mathematical expression (of parameters) for COMSOL to evaluate.
- "materials": List of JSON Objects for each material assignment in a part instance (*usually* this is just one material; contacts with recessed domains will have one material for the conductor and one material for the recessed domain as the part instance will create two separate domains with independent selections)
    - "info": The value (String) is the function of the domain in the FEM (i.e., "medium", cuff "fill", cuff "insulator", contact "conductor", and contact "recess") that is used to assign material properties to a selected domain. The value will match a key in the "conductivities" JSON Object in ***Model***.
    - "label_index": The value (Integer) corresponds to the index of the selection for the domain (in im.labels, defined independently for each primitive case in Part.createCuffPartPrimitive() – see code in src/model/Part.java) to be assigned to the material function. Note that im.labels are indexed starting at 0.

"params": The value is a list of JSON Objects, one for each parameter used to define parameterizations of part primitives in COMSOL's Global Definitions. The structure of each JSON Object is consistent with the format of the dialogue boxes in each "Parameters" group (i.e., "Name", "Expression", "Description") where the parameters are populated in the COMSOL GUI:

- "name": The value (String) is the name of the parameter.
- "expression": The value (String) is the expression/constant with units that COMSOL will evaluate. Therefore, if the value is a constant, units wrapped in "[]" are required (e.g., "5

[um]”). If the value is an expression relating other parameters (that already are dimensioned with units) with known mathematical expressions (e.g., multiply “*”, divide “/”, add “+”, subtract “-”, exponent “^”, trigonometric formulas: “sin()”, “cos()”, “tan()”, “asin()”, “acos()”, “atan()”), do not add units after the expression.

- “description”: The value (String) can be empty (i.e., “”) or may contain a description of the parameter such as a reference to the source (e.g., published patent/schematics) or a note to your future self.

“expandable”: The value (Boolean) tells the system whether to expect the implementation of the cuff in COMSOL to be able to expand beyond the manufactured resting cuff diameter to fit around a nerve. For a cuff to be expandable, it must be constructed from part primitives that have been parameterized to expand as a function of “R_in”. See config/system/cuffs/Purdue.json for an example of an expandable cuff.

“angle_to_contacts_deg”: The value (Double, units: degrees) defines the angle to the contact point of the nerve on the inside of the cuff of the cuff (measured counterclockwise from the +x-axis before any rotation/deformation of the cuff).

“offset”: The JSON Object contains keys that are names of parameters defined in the list of “params” in this “preset” cuff file. If the inner diameter of the cuff must expand to allow for additional distance between the nerve and “R_in” (the inner surface of the cuff insulator), the user adds key-value pairs for the offset buffer here. For each known parameter key, the user sets the value (Double) to be the multiplicative factor for the parameter key. The list of key-value pairs can be empty, as is the case for most cuffs. Once offset is added to a cuff, the values are automatically used in Runner’s compute_cuff_shift() method.

For example usage of this functionality, see config/system/cuffs/Purdue.json as replicated below:

```
"offset": {
  "sep_wire_P”: 1, // sep_wire_P is the separation between the wire contact and the
                // inner diameter of the cuff
  “r_wire_P”: 2    // r_wire_P is the radius of the circular cross section of the
                // wire contact (i.e., half of the wire’s gauge)
}

// the above JSON Object adds an offset buffer between the nerve and the inner
// diameter of the cuff of : (1*sep_wire_P) + (2*r_wire_P)
```
