# Supplementary material for: ASCENT (Automated Simulations to Characterize Electrical Nerve Thresholds): A pipeline for sample-specific computational modeling of electrical stimulation of peripheral nerves
Source: PLoS Comput Biol. 2021 Sep 7;17(9):e1009285. doi: 10.1371/journal.pcbi.1009285 (PMC8423288; doi:10.1371/journal.pcbi.1009285)
Supplement: S18 Text — Creating new part primitives. (PDF) [file pcbi.1009285.s018.pdf]

1 S18 Text

Appendix. Creating new part primitives

Though we provide a library of part primitives to assemble representations of many cuff electrodes as shown in examples/parts/sandbox.mph, users may find it necessary to add their own part primitives for representing their custom cuff electrode designs. Use the following instructions as a guide and link to resources for creating a new part primitive.

The COMSOL GUI has a “Geometry Parts” node under the “Global Definitions”. The pipeline adds part primitives—i.e., the geometry of different pieces of cuff electrodes (e.g., contacts (S16 Text Figure A), insulators (S16 Text Figure B)), cuff fill (e.g., encapsulation tissue, mineral oil, saline), or medium (e.g., surrounding muscle, fat)—as “parts” under “Geometry Parts”. Their resulting volumes (domains), surfaces, and points (used for point current sources) are added to the list of “cumulative selections” which are later used to assign appropriate mesh settings, material properties, and boundary conditions.

1. Create and label your part. Open up examples/parts/sandbox.mph, secondary-click on “Geometry Parts”, choose “3D Part”. Give your part an appropriate label as it will later be used in creating “preset” cuff JSON files and as a flag for the primitive in our Java Part class (src/model/Part.java).

2. Define your part’s geometry. Secondary-click on the new part under “Geometry Parts” to add the geometry operations required to construct your cuff (e.g., “Block”, “Cone”, “Cylinder”, “Sphere”, “More Primitives”, “Work Plane”). See the operations under the other part primitives in sandbox.mph as a guide in creating your geometry, as well as COMSOL's documentation (https://cdn.comsol.com/doc/5.5/IntroductionToCOMSOLMultiphysics.pdf, in particular their “Appendix A – Building a Geometry”) and “Introductory Video Series on How to Build Geometries in COMSOL” (https://www.comsol.com/video/introductory-video-series-on-how-to-build-geometries-in-comsol). In creating your geometry, carefully label each operation to improve readability of your geometry operations. Taking care to label the operations will not only help to communicate to other users and your future self what operations must occur, but also will help you in cleaning up your Java code in the Part class (part of step 6). The dimensions and locations of part geometries should refer to “Input Parameters” or parameters stored in a parameters group under “Global Definitions”.

3. Assign each feature of interest (i.e., volumes/domains, surfaces, points) to a “Cumulative selection”. The final form of domains that you will want to assign to a material property need to be assigned to a “Cumulative selection”, which can be found for the final geometry operation under the “Settings” tab. In “Settings”, under the “Contribute to” drop down menu within the “Selections of Resulting Entities” section, the first time you refer to a cumulative selection you will need to click the “New” button and type in the name of the selection. Again, take care to give an

informative label. Our convention, though not required, is to make the cumulative selection names in all capitals to improve code readability.

4. Compact sandbox.mph file history. File -> Compact History removes any operations you may have tested but did not ultimately use to create your part.

5. Export Java code. Go to File -> Save As, give a meaningful path/file name, and change the file type to “Model File for Java (*.java)”.

6. Add the operations for your part primitive to the Part.createCuffPartPrimitive() method in Java (src/model/Part.java). With the text editor of your choice, open the newly created *.java file. Toward the bottom of the file, the operations you just performed in COMSOL are contained in a block of code. Performing a “Command+F” (on Mac) or “Control+F” (on Windows) for your new part primitive’s name that you gave in the GUI (from step 1) should take you to the first line of the code block of interest, which looks like:

“model.geom(“part<#>”).label(<your part’s name>);

All subsequent lines starting with “model.geom(“part<#>”)” are of interest. Copy them to your clipboard. With Part.createEnvironmentPartPrimitive() as a guide (since it is by far the simplest and most contained “primitive” – in reality it is just a cylinder contributed to the “MEDIUM” cumulative selection), add your lines to Part.createCuffPartPrimitive().

- Add a “case” in the switch-case (e.g., case “TubeCuff_Primitive”). Within this case-block, all operations for the new primitive will be added.
- For each line in your code (copied from the exported *.java file) that begin with “model.geom.(“part<#>”).inputParam().set(“<my_parameter>”, “<default_value>”), at the top of your new case-block, add the following line to set the “Input Parameters” you established in the COMSOL GUI:
    - mp.set(“<my_parameter>”, “<default_value>”)
- Still looking at Part.createEnvironmentPartPrimitive() as an example, now create your list of “selections” in “im.labels” which are the lines that follow after the “Input Parameters” are defined. Then add the for loop that loops over the im.labels [String, …] adding them to COMSOL’s selections. These lines will look like:

```
im.labels = new String[] {
  “<MY_CUMULATIVE SELECTION_1>”,  // note: This is index 0
  “<MY_CUMULATIVE SELECTION_2>”,  // note: This is index 1
  …
}

for (String cselLabel : im.labels) {
  model.geom(id).selection().create(im.next(“csel”, cselLabel),
“CumulativeSelection”).label(cselLabel)
}
```

- The lines that follow the Cumulative Selection labeling add the geometry features of the COMSOL part which COMSOL has also conveniently exported for you in the *.java file. See S26 Text for an explanation of our Java IdentifierManager utility class that we created to abstract away from COMSOL's indexing system to improve code readability. Our "IdentifierManager" class enables the user to access previously defined selection tags by an informative label programmatically. Part.createEnvironmentPartPrimitive() and Part.createCuffPartPrimitive() are also great working examples of how we use the COMSOL plugin in an IDE to clean up the code (e.g., creating a COMSOL "GeomFeature" to shorten the length of each line).
- End the case for your new part primitive with a "break;" (this is very important!)

7. Add the operations for your part primitive to the Part.createCuffPartInstance() method (src/model/Part.java).

- Create a List[String] containing the "Input Parameters" you established in the COMSOL GUI. These lines will look like:

```
String[] myPrimitiveParameters = {
  "<my_parameter1>",
  "<my_parameter2>",
  … // for all Input Parameters
}
```

- Add a for loop that adds all the "Input Parameters" to the part instance. These lines will look like:

```
for (String param : myPrimitiveParameters) {
  partInstance.setEntry("inputexpr", param, (String) itemObject.get(param));
}
```

- Our primitives have an additional (optional) section for selection imports. Each defined selection used in your geometry operations will be visible in the "Contribute to" drop-down menu unless you toggle each selection "off". An example of how to "clean up" your selections imported is shown below:

```
// imports

// so that the program only imports selections that are used
partInstance.set("selkeepnoncontr", false);

// to selectively import the DOMAIN for whatever selection index 0 is in myLabels (defined in im.labels in
// createCuffPartPrimitive()). To exclude, "off" instead of "on".
partInstance.setEntry("selkeepdom", instanceID + "_" + myIM.get(myLabels[0] + ".dom", "on"));
```

// to selectively import the BOUNDARY for whatever selection index 0 is in myLabels (defined in im.labels
// in createCuffPartPrimitive()). To exclude, “off” instead of “on”.
partInstance.setEntry(“selkeepbnd”, instanceID + “_” + myIM.get(myLabels[0] + “.bnd”, “on”));

// to selectively import the POINT for whatever selection index 0 is in myLabels (defined in im.labels
// in createCuffPartPrimitive()). To exclude, “off” instead of “on”.
partInstance.setEntry(“selkeeppnt”, instanceID + “_” + myIM.get(myLabels[0] + “.pnt”, “on”));

- End the case for your new part instance with a “break;” (this is very important!)

1. (Step is optional but recommended). Add your new part to examples/parts/sandbox.mph. Simply save your sandbox.mph file as a *.mph, if you have not already, for future ability to assemble cuffs using your new part in the COMSOL GUI.
