# Supplementary material for: ASCENT (Automated Simulations to Characterize Electrical Nerve Thresholds): A pipeline for sample-specific computational modeling of electrical stimulation of peripheral nerves
Source: PLoS Comput Biol. 2021 Sep 7;17(9):e1009285. doi: 10.1371/journal.pcbi.1009285 (PMC8423288; doi:10.1371/journal.pcbi.1009285)
Supplement: S19 Text — Cuff placement on nerve. (PDF) [file pcbi.1009285.s019.pdf]

1 S19 Text

Appendix. Cuff placement on nerve

This section provides an overview of how the cuff is placed on the nerve. The compute_cuff_shift() method within Runner (src/runner.py) determines the cuff's rotation around the nerve and translation in the (x,y)-plane. The pipeline imports the coordinates of the traces for the nerve tissue boundaries saved in samples/<sample_index>/slides/0/0/sectionwise2d/ which are, by convention, shifted such that the centroid of the nerve is at the origin (0,0) (i.e., nerve centroid from best-fit ellipse if nerve trace (n.tif) is provided, inner or outer best-fit ellipse centroid for monofascicular nerves without nerve trace). Importantly, the nerve sample cross section is never moved from or rotated around the origin in COMSOL. By maintaining consistent nerve location across all ***Model's*** for a ***Sample***, the coordinates in fibersets/ are correct for any orientation of a cuff on the nerve.

ASCENT has different CuffShiftModes (i.e., "cuff_shift" parameter in ***Model***) that control the translation of the cuff (i.e., "shift" JSON Object in ***Model***) and default rotation around the nerve (i.e., "pos_ang" in ***Model***). Runner's compute_cuff_shift() method is easily expandable for users to add their own CuffShiftModes to control cuff placement on the nerve.

The rotation and translation of the cuff are populated automatically by the compute_cuff_shift() method based on sample morphology, parameterization of the "preset" cuff, and the CuffShiftMode, and are defined in the "cuff" JSON Object ("shift" and "rotate") in ***Model***.

For "naïve" CuffShiftModes (i.e., "NAIVE_ROTATION_MIN_CIRCLE_BOUNDARY", "NAIVE_ROTATION_TRACE_BOUNDARY") the cuff is placed on the nerve with rotation according to the parameters used to instantiate the cuff from part primitives (S16 - S18 Text). If the user would like to rotate the cuff from beyond this position, they may set the "add_ang" parameter in ***Model*** (S8 Text). For naïve CuffShiftModes, the cuff is shifted along the vector from (0,0) in the direction of the "angle_to_contacts_deg" parameter in the "preset" JSON file. "NAIVE_ROTATION_MIN_CIRCLE_BOUNDARY" CuffShiftMode moves the cuff toward the nerve until the nerve's minimum radius enclosing circle is within the distance of the "thk_medium_gap_internal" parameter for the cuff. "NAIVE_ROTATION_TRACE_BOUNDARY" CuffShiftMode moves the cuff toward the nerve until the nerve's outermost Trace (i.e., for monofascicular nerve an inner or outer, and same result as "NAIVE_ROTATION_MIN_CIRCLE_BOUNDARY" for nerve's with epineurium) is within the distance of the "thk_medium_gap_internal" parameter for the cuff.

For "automatic" CuffShiftModes (i.e., "AUTO_ROTATION_MIN_CIRCLE_BOUNDARY", "AUTO_ROTATION_TRACE_BOUNDARY") the cuff is rotated around the nerve based on the size and position of the nerve's fascicle(s) before the cuff is moved toward the nerve sample (Figure A). The point at the intersection of the vector from (0,0) in the direction of the

“angle_to_contacts_deg” parameter in the “preset” JSON file with the cuff (i.e., cuff’s “center” in following text) is rotated to meet a specific location of the nerve/monofascicle’s surface. Specifically, the center of the cuff is rotated around the nerve to make (0,0), the center of the cuff, and ***Sample’s*** “fascicle_centroid” (computed with Slide’s fascicle_centroid() method, which calculates the area and centroid of each inner and then averages the inners’ centroids weighted by each inner’s area) colinear. If the user would like to rotate the cuff from beyond this position, they may set the “add_ang” parameter in ***Model*** (S8 Text). The user may override the default “AUTO” rotation of the cuff on the nerve by adding an orientation mask (a.tif) to align a certain surface of the nerve sample with the cuff’s center (S11 Text). “AUTO_ROTATION_MIN_CIRCLE_BOUNDARY” CuffShiftMode moves the cuff toward the nerve until the nerve’s minimum radius enclosing circle is within the distance of the “thk_medium_gap_internal” parameter for the cuff. “AUTO_ROTATION_TRACE_BOUNDARY” CuffShiftMode moves the cuff toward the nerve until the nerve’s outermost Trace (i.e., for monofascicular nerve an inner or outer, and same result as “AUTO_ROTATION_MIN_CIRCLE_BOUNDARY” for nerve’s with epineurium) is within the distance of the “thk_medium_gap_internal” parameter for the cuff.

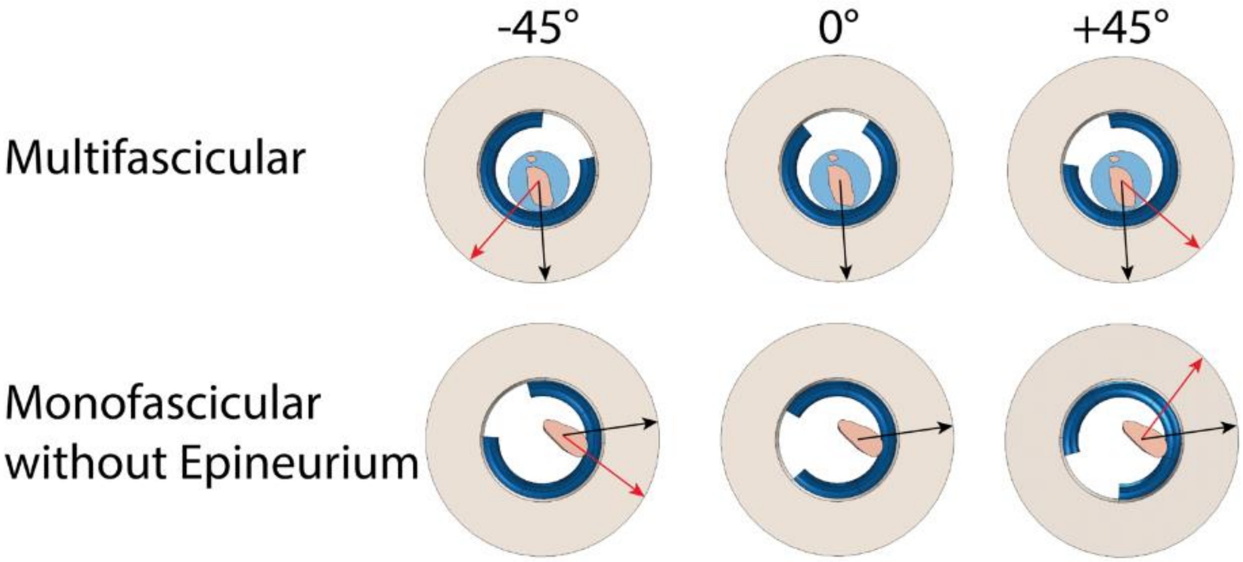

Figure A. Demonstration of cuff placement on a multifascicular nerve (top) and a monofascicular nerve without epineurium (bottom) with the same “preset” cuff (Purdue.json) for three different cuff rotations using the “AUTO_ROTATION_TRACE_BOUNDARY” CuffShiftMode. The cuff rotations are different in the top and bottom rows since the point on the surface of the nerve sample closest to the most endoneurium is unique to each sample (black arrows). Additional angles of rotation were applied to the cuff directly using the “add_ang” parameter in the **Model’s** “cuff” JSON Object (red arrows).

The default z-position of each part along the nerve is defined in the “preset” cuff JSON file by the expression assigned to the part instance’s “Center” parameter (referenced to z = 0 at one end of the model’s proximal cylindrical domain). However, if the user would like to move the entire “preset” cuff along the length of the nerve, in the “cuff” JSON Object within ***Model***, the user may change the “z” parameter.

Since some cuffs can open in response to a nerve diameter larger than the manufactured cuff's inner diameter, they maybe be parameterized as a function of "R_in". In this case, in the "preset" cuff JSON, the "expandable" Boolean parameter is true. If the cuff is "expandable" and the minimum enclosing diameter of the sample is larger than the cuff, the program will modify the angle for the center of the cuff to preserve the length of materials. If the cuff is not expandable and the sum of the minimum enclosing circle radius of the sample and "thk_medium_gap_internal" are larger than the inner radius of the cuff, an error is thrown as the cuff cannot accommodate the sample morphology.

The inner radius of the cuff is defined within the list of "params" in each "preset" cuff configuration file as "R_in", and a JSON Object called "offset" contains a parameterized definition of any additional buffer required within the inner radius of the cuff (e.g., exposed wire contacts as in the Purdue.json cuff "preset"). Each key in the "offset" JSON Object must match a parameter value defined in the "params" list of the cuff configuration file. For each key in "offset", the value is the multiplicative coefficient for the parameter key to include in a sum of all key-value products. For example, in Purdue.json:

```
"offset": {
  "sep_wire_P": 1, // separation between outer boundary of wire contact and internal
                // surface of insulator
  "r_wire_P": 2 // radius of the wire contact's gauge
}
```

This JSON Object in Purdue.json will instruct the system to maintain added separation between the internal surface of the cuff and the nerve of:

$$(1) * sep\_wire\_P \quad + (2) * r\_wire\_P$$
