# Supplementary material for: ASCENT (Automated Simulations to Characterize Electrical Nerve Thresholds): A pipeline for sample-specific computational modeling of electrical stimulation of peripheral nerves
Source: PLoS Comput Biol. 2021 Sep 7;17(9):e1009285. doi: 10.1371/journal.pcbi.1009285 (PMC8423288; doi:10.1371/journal.pcbi.1009285)
Supplement: S20 Text — Fiberset. (PDF) [file pcbi.1009285.s020.pdf]

1 S20 Text

Appendix. Fiberset

Runner's run() method first loads JSON configuration files for ***Sample***, ***Model***, and ***Sim*** into memory and instantiates a Python Sample class. The Sample instance produces two-dimensional CAD files that define nerve and fascicle tissue boundaries in COMSOL from the input binary masks. The run() method also instantiates Python Simulation classes using the ***Model*** and ***Sim*** configurations to define the coordinates of "fibersets" where "potentials" are sampled in COMSOL to be applied extracellularly in NEURON and to define the current amplitude versus time stimulation waveform used in NEURON ("waveforms"). The Simulation class is unique in that it performs operations both before and after the program performs a handoff to Java for COMSOL operations. Before the handoff to Java, each Simulation writes fibersets/ and waveforms/ to file, and after the Java operations are complete, each Simulation builds folders (i.e., n_sims/), each containing NEURON code and input data for simulating fiber responses for a single ***Sample***, ***Model***, fiberset, waveform, and contact weighting. Each instance of the Simulation class is saved as a Python object using Saveable (S9 Text), which is used for resuming operations after the handoff() method to Java is completed.

Within the write_fibers() method of the Python Simulation class, the Python Fiberset class is instantiated with an instance of the Python Sample class, ***Model***, and ***Sim***. Fiberset's generate() method creates a set of (x,y,z)-coordinates for each Fiberset defined in ***Sim***. The (x,y)-coordinates in the nerve cross section and z-coordinates along the length of the nerve are saved in fibersets/.

Fiberset's method _generate_xy() (first character being an underscore indicates intended for use only by the Fiberset class) defines the coordinates of simulated fibers in the cross section of the nerve according to the "xy_parameters" JSON Object in ***Sim*** (S8 Text). The pipeline defines (x,y)-coordinates of the fibers in the nerve cross section according to the user's selection of sampling rules (CENTROID, UNIFORM_DENSITY, UNIFORM_COUNT, and WHEEL); the pre-defined modes for defining fiber locations are easily expandable. To add a new mode for defining (x,y)-coordinates, the user must add a "FiberXYMode" in src/utils/enums.py (S6 Text) and add an IF statement code block in _generate_xy() containing the operations for constructing "points" (List[Tuple[float]]). The user must add the parameters to define how fibers are placed in the nerve within the "xy_parameters" JSON Object in ***Sim***. In ***Sim***, the user may control the "plot" parameter (Boolean) in the "fibers" JSON Object to create a figure of fiber (x,y)-coordinates on the slide. Alternatively, the user may plot a Fibserset using the plot_fiberset.py script (S33 Text).

Fiberset's private method _generate_z() defines the coordinates of the compartments of simulated fibers along the length of the nerve based on global parameters in config/system/fiber_z.json and simulation-specific parameters in the "fibers" JSON Object in ***Sim*** (i.e., "mode", "diameter", "min", "max", and "offset").
