# Supplementary material for: ASCENT (Automated Simulations to Characterize Electrical Nerve Thresholds): A pipeline for sample-specific computational modeling of electrical stimulation of peripheral nerves
Source: PLoS Comput Biol. 2021 Sep 7;17(9):e1009285. doi: 10.1371/journal.pcbi.1009285 (PMC8423288; doi:10.1371/journal.pcbi.1009285)
Supplement: S21 Text — Implementation of NEURON fiber models. (PDF) [file pcbi.1009285.s021.pdf]

1 S21 Text

Appendix. Implementation of NEURON fiber models

1.1 Myelinated fiber models

The CreateAxon_Myel.hoc file is loaded in Wrapper.hoc if the user chooses either “MRG_DISCRETE” or “MRG_INTERPOLATION”. The length of each section in NEURON varies depending on both the diameter and the “FiberGeometry” mode chosen in ***Sim***.

1.1.1 MRG discrete diameter (as previously published)

The “FiberGeometry” mode “MRG_DISCRETE” in ***Sim*** instructs the program to simulate a double cable structure for mammalian myelinated fibers [1,2]. In the pipeline, we refer to this model as “MRG_DISCRETE” since the model’s geometric parameters were originally published for a *discrete* list of fiber diameters: 1, 2, 5.7, 7.3, 8.7, 10, 11.5, 12.8, 14.0, 15.0, and 16.0 μm. Since the MRG fiber model has distinct geometric dimensions for each fiber diameter, the parameters are stored in config/system/fiber_z.json as lists in the “MRG_DISCRETE” JSON Object, where a value’s index corresponds to the index of the discrete diameter in “diameters”. The parameters are used by the Fiberset class to create fibersets/ (i.e., coordinates to probe potentials/ from COMSOL) for MRG fibers.

1.1.2 MRG interpolated diameters

The “FiberGeometry” mode “MRG_INTERPOLATION” in ***Sim*** instructs the program to simulate a double cable structure for mammalian myelinated fibers for any diameter fiber between 2 and 16 μm (throws an error if not in this range) by using an *interpolation* over the originally published fiber geometries [1,2]. In the pipeline, we refer to this model as “MRG_INTERPOLATION” since it enables the user to simulate any fiber diameter between the originally published diameters.

The parameters in the “MRG_INTERPOLATION” JSON Object in config/system/fiber_z.json are used by the Fiberset class to create fibersets/ (i.e., coordinates at which to sample potentials/ from COMSOL) for interpolated MRG fibers. Since the parameter values relate to fiber “diameter” as a continuous variable, the expressions for all the dimensions that change with fiber diameter, as shown in Figure A, are stored as a String that is computed using Python’s built-in “eval()” function.

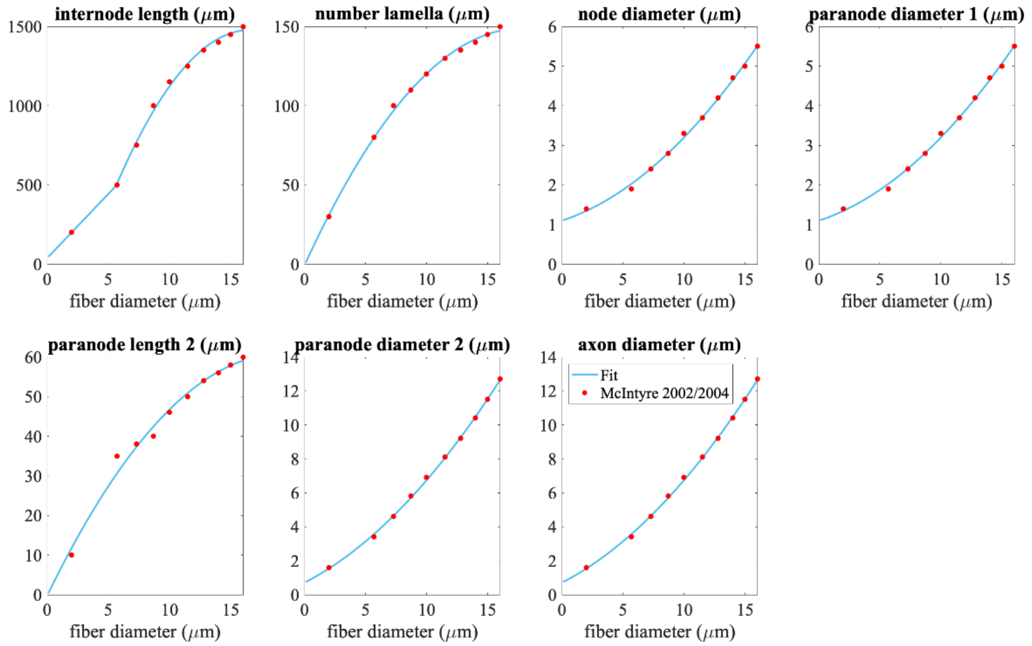

Figure A. Piecewise polynomial fits to published MRG fiber parameters. Single quadratic fits were used for all parameters except for internode length, which has a linear fit below 5.643 µm (using MRG data at 2 and 5.7 µm) and a single quadratic fit at diameters greater than or equal to 5.643 µm (using MRG data >= 5.7 µm); 5.643 µm is the fiber diameter at which the linear and quadratic fits intersected. The fiber diameter is the diameter of the myelin. “Paranode 1” is the MYSA section, “paranode 2” is the FLUT section, and “internode” is the STIN section. The axon diameter is the same for the node of Ranvier and MYSA (“node diameter”), as well as for the FLUT and STIN (“axon diameter”). The node and MYSA lengths are fixed at 1 and 3 µm, respectively, for all fiber diameters.

We compared fiber activation thresholds between the originally published MRG fiber models and the interpolated MRG ultrastructure (evaluated at the original diameters) at a single location in a rat cervical vagus nerve stimulated with the bipolar Purdue cuff. Each fiber was placed at the centroid of the best-fit ellipse of the monofascicular nerve sample. The waveform was a single biphasic pulse using “BIPHASIC_PULSE_TRAIN_Q_BALANCED_UNEVEN_PW” with 100 µs for the first phase, 100 µs interphase (0 mA), and 400 µs for the second phase (cathodic/anodic at one contact and anodic/cathodic at the other contact). The thresholds between the originally published models and the interpolation of the MRG fiber diameters are compared in Figure B

below. The threshold values were determined using a binary search until the upper and lower bound stimulation amplitudes were within 1%.

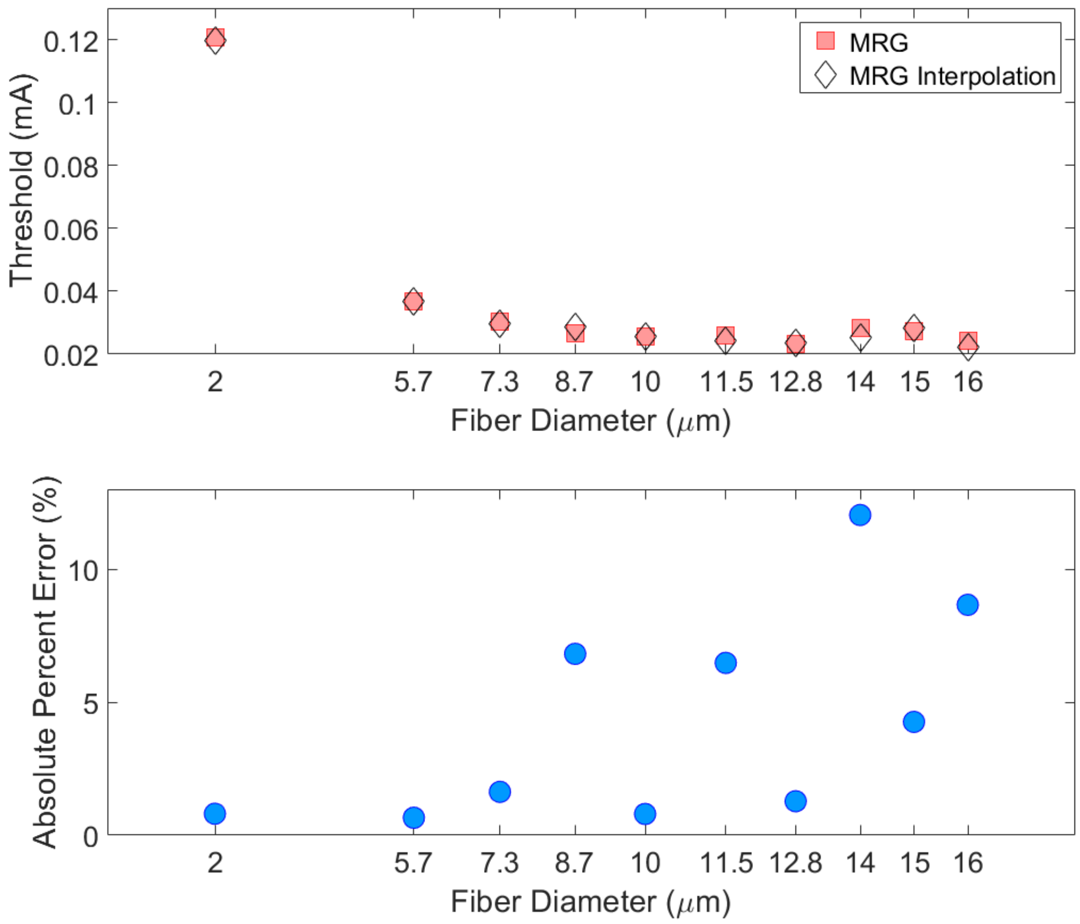

Figure B. Comparison of thresholds between the originally published models and the interpolation of the MRG fiber diameters (evaluated at the original diameters). Thresholds are expected to vary between the originally published models and the interpolated fiber geometries given their slightly different ultrastructure parameters (Figure A). Used original MRG thresholds as reference.

1.2 Unmyelinated fiber models

The pipeline includes several unmyelinated (i.e., C-fiber) models [3–5]. Users should be aware of the “delta_zs” parameter that they are using in config/system/fiber_z.json, which controls the spatial discretization of the fiber (i.e., the length of each section).

1.3 References

1. McIntyre CC, Grill WM, Sherman DL, Thakor N V. Cellular effects of deep brain stimulation: model-based analysis of activation and inhibition. J Neurophysiol. 2004 Apr;91(4):1457–69. Available from: https://doi.org/10.1152/jn.00989.2003 PMID: 14668299
2. McIntyre CC, Richardson AG, Grill WM. Modeling the excitability of mammalian nerve

fibers: influence of afterpotentials on the recovery cycle. J Neurophysiol. 2002 Feb;87(2):995–1006. Available from: https://doi.org/10.1152/jn.00353.2001 PMID: 11826063

3. Sundt D, Gamper N, Jaffe DB. Spike propagation through the dorsal root ganglia in an unmyelinated sensory neuron: a modeling study. J Neurophysiol. 2015 Dec;114(6):3140–53. Available from: https://doi.org/10.1152/jn.00226.2015 PMID: 26334005
4. Tigerholm J, Petersson ME, Obreja O, Lampert A, Carr R, Schmelz M, et al. Modeling activity-dependent changes of axonal spike conduction in primary afferent C-nociceptors. J Neurophysiol. 2014 May;111(9):1721–35. Available from: https://doi.org/10.1152/jn.00777.2012 PMID: 24371290
5. Rattay F, Aberham M. Modeling axon membranes for functional electrical stimulation. IEEE Trans Biomed Eng. 1993 Dec;40(12):1201–9. Available from: https://doi.org/10.1109/10.250575 PMID: 8125496
