# Supplementary material for: ASCENT (Automated Simulations to Characterize Electrical Nerve Thresholds): A pipeline for sample-specific computational modeling of electrical stimulation of peripheral nerves
Source: PLoS Comput Biol. 2021 Sep 7;17(9):e1009285. doi: 10.1371/journal.pcbi.1009285 (PMC8423288; doi:10.1371/journal.pcbi.1009285)
Supplement: S22 Text — Simulation protocols. (PDF) [file pcbi.1009285.s022.pdf]

1 S22 Text

Appendix. Simulation protocols

Fiber response to electrical stimulation is computed by applying electric potentials along the length of a fiber from COMSOL as a time-varying signal in NEURON. The stimulation waveform, saved in a n_sim's data/inputs/ directory as waveform.dat, is unscaled (i.e., the maximum current magnitude at any timestep is +/-1), and is then scaled by the current amplitude in RunSim.hoc to either simulate fiber thresholds of activation or block with a binary search algorithm, or response to set amplitudes.

1.1 Binary search

In searching for activation thresholds (i.e., the minimum stimulation amplitude required to generate a propagating action potential) or block thresholds (i.e., the minimum stimulation amplitude required to block the propagation of an action potential) in the pipeline, the NEURON code uses a binary search algorithm.

The basics of a binary search algorithm are as follows. By starting with one value that is above threshold (i.e., upper bound) and one value that is below threshold (i.e., lower bound), the program tests the midpoint amplitude to determine if it is above or below threshold. If the midpoint amplitude is found to be below threshold, the midpoint amplitude becomes the new lower bound. However, if the midpoint amplitude is found to be above threshold, the midpoint amplitude becomes the new upper bound. At each iteration of this process, half of the remaining amplitude range is removed. The process is continued until the termination criteria is satisfied (e.g., some threshold resolution tolerance is achieved). The average performance of a binary search algorithm is $O(\log(n))$ where n is the number of elements in the search array (i.e., linearly spaced range of amplitudes).

In the pipeline, the binary search protocol parameters (i.e., activation or block criteria, threshold criteria, method for searching for starting upper- and lower bounds, or termination criteria) are contained in the "protocol" JSON Object within ***Sim*** (S8 Text).

1.2 Activation threshold protocol

The pipeline has a NEURON simulation protocol for determining thresholds of activation of nerve fibers in response to extracellular stimulation. Threshold amplitude for fiber activation is defined as the minimum stimulation amplitude required to initiate a propagating action potential. The pipeline uses a binary search algorithm to converge on the threshold amplitude. Current amplitudes are determined to be above threshold if the stimulation results in at least n_AP propagating action potentials detected at 75% of the fiber's length (note: location can be specified

by user with “ap_detect_location” parameter in ***Sim***) (S8 Text). The parameters for control over the activation threshold protocol are found in ***Sim*** within the “protocol” JSON Object (S8 Text).

1.3 Block threshold protocol

The pipeline has a NEURON simulation protocol for determining block thresholds for nerve fibers in response to extracellular stimulation. Threshold amplitude for fiber block is defined as the minimum stimulation amplitude required to block a propagating action potential. The simulation protocol for determining block thresholds starts by delivering the blocking waveform through the cuff. After a user-defined delay during the stimulation onset period, the protocol delivers a test pulse (or a train of pulses if the user chooses) where the user placed it (see “ind” parameter in ***Sim*** within the “intracellular_stim” JSON Object (S8 Text)), near the proximal end. The code checks for action potentials near the distal end of the fiber (see “ap_detect_location” parameter in ***Sim*** within the “threshold” JSON Object (S8 Text)). If at least one action potential is detected, then transmission of the test pulse occurred (i.e., the stimulation amplitude is below block threshold). However, the absence of an action potential indicates block (i.e., the stimulation amplitude is above block threshold). The pipeline uses a binary search algorithm to converge on the threshold amplitude. The parameters for control over the block threshold protocol are found in ***Sim*** within the “protocol” JSON Object (S8 Text).

The user must be careful in setting the initial upper and lower bounds of the binary search for block thresholds. Especially for small diameter myelinated fibers, users must be aware of and check for re-excitation using a stimulation amplitude sweep [1].

1.4 Response to set amplitudes

Alternatively, users may simulate the response of nerve fibers in response to extracellular stimulation for a user-specified set of amplitudes. The “protocol” JSON Object within ***Sim*** contains the set of amplitudes that the user would like to simulate (S8 Text).

1.5 References

1. Pelot NA, Behrend CE, Grill WM. Modeling the response of small myelinated axons in a compound nerve to kilohertz frequency signals. J Neural Eng. 2017 Aug;14(4):46022. Available from: https://doi.org/10.1088/1741-2552/aa6a5f PMID: 28361793
