# Supplementary material for: ASCENT (Automated Simulations to Characterize Electrical Nerve Thresholds): A pipeline for sample-specific computational modeling of electrical stimulation of peripheral nerves
Source: PLoS Comput Biol. 2021 Sep 7;17(9):e1009285. doi: 10.1371/journal.pcbi.1009285 (PMC8423288; doi:10.1371/journal.pcbi.1009285)
Supplement: S23 Text — ModelWrapper class. (PDF) [file pcbi.1009285.s023.pdf]

1 S23 Text

Appendix. ModelWrapper class

1.1 ModelWrapper

The ModelWrapper class in Java takes inputs of the ASCENT_PROJECT_PATH (env.json, S7 and S8 Text) and a list of ***Run*** paths. ModelWrapper contains a COMSOL "model" object, model source directory (String), model destination directory (String), an "IdentifierManager" (S26 Text), and HashMaps with key-value pairs linking COMSOL domains to unions of domains. ModelWrapper has accessor methods getModel() for retrieving the model object, getRoot() for retrieving the project path's root String, and getDest() for retrieving the default saving destination String. ModelWrapper also has mutator methods for changing an instance's root String (setRoot()) and default saving destination String (setDest()).

ModelWrapper's main() method starts an instance of COMSOL and loads ***Run***, ***Sample***, ***Model***, and ***Sim*** configurations as JSON Objects into memory. We developed Java class JSONio (S26 Text) for reading and writing JSON Objects to file.

Since each ***Run*** contains a list of ***Model*** and ***Sim*** configurations for a single ***Sample*** (note: n_sims/ are created for all combinations of ***Model*** and ***Sim*** for the ***Sample*** in a ***Run***), ModelWrapper iterates over ***Model*** configurations (e.g., different cuff electrodes or material assignments) to define the FEM geometry, mesh, assign boundary conditions and physics, and solve. The resulting FEM potentials are obtained for 1 mA applied to one of the electrode contacts while the electric potential on the other contacts is floating (i.e., condition of continuity); this is repeated for each contact to define the "bases" of the solution space [1]. For each ***Sim***, the program then creates a superposition of the "bases" for extracellular potentials at the coordinates defined in fibersets/ and ss_coords/ (i.e., the coordinates along the length of the nerve used to "super-sample" potentials for later creating potentials/ without the need for COMSOL). We wrote the code such that the program will continue with creating potentials/, ss_bases/ (i.e., the potentials along the length of the nerve corresponding 1:1 to the coordinates saved in ss_coords/, which are added together according to the contact weighting defined by "active_srcs" in ***Sim*** to create potentials/ for specific fiber models), and NEURON simulations for any remaining ***Model*** indices even if the processes for a single ***Model*** fails. For each ***Model***, the program appends a Boolean to "models_exit_status" in ***Run*** (true if successful, false if not successful).

1.2 References

1. Pelot NA, Thio BJ, Grill WM. Modeling Current Sources for Neural Stimulation in COMSOL. Front Comput Neurosci [Internet]. 2018;12:40. Available from: https://doi.org/10.3389/fncom.2018.00040 PMID: 29937722
