# Supplementary material for: ASCENT (Automated Simulations to Characterize Electrical Nerve Thresholds): A pipeline for sample-specific computational modeling of electrical stimulation of peripheral nerves
Source: PLoS Comput Biol. 2021 Sep 7;17(9):e1009285. doi: 10.1371/journal.pcbi.1009285 (PMC8423288; doi:10.1371/journal.pcbi.1009285)
Supplement: S24 Text — Making geometries in COMSOL (Part class). (PDF) [file pcbi.1009285.s024.pdf]

1 S24 Text

Appendix. Making geometries in COMSOL (Part class)

1.1 Part.createEnvironmentPartPrimitive()

The createEnvironmentPartPrimitive() method in Java (src/model/Part.java) creates a “part” within the “Geometry Parts” node of the COMSOL “model” object to generalize the cylindrical medium surrounding the nerve and electrode. Programmatically selecting domains and surfaces in COMSOL requires that geometry operations be contributed to “selections” (csel<#>). In this simple example of a part primitive, the im.labels String[] contains the string “MEDIUM” which is used to label the COMSOL selection (csel<#>) for the medium domain by association with an IdentifierManager (S26 Text). When the geometry of the primitive is built, the resulting medium domain’s csel<#> can be accessed instead with the key “MEDIUM” in the IdentifierManager, thereby improving readability and accessibility when materials and boundary conditions are assigned in the createEnvironmentPartInstance() method. Furthermore, if the operations of a part primitive are modified, the indexing of the csel<#> labels are automatically handled.

1.2 Part.createCuffPartPrimitive()

The createCuffPartPrimitive() method in Java (src/model/Part.java) is analogous to createEnvironmentPartPrimitive(), except that it contains the operations required to define cuff part geometries, which are generally more complex. Examples of cuff part primitives include standard geometries for contact conductors (e.g., Ribbon Contact Primitive, Wire Contact Primitive, Circle Contact Primitive, and Rectangular Contact Primitive), cuff insulation (e.g., Tube Cuff), cuff fill (e.g., saline, mineral oil), and specific interactions of an cuff insulator and electrode contact (e.g., LivaNova-inspired helical coil) (S16 Text).

1.3 Part Instances

Part instances are a COMSOL Geometry Feature (“pi<#>”) in the “Geometry” node based on user-defined input parameters stored in ***Model*** and default parameters for “preset” cuffs. A part instance is an instantiation of a part primitive previously defined in the COMSOL “model” object and will take specific form based on its input parameters.

1.3.1 Part.createEnvironmentPartInstance()

The createEnvironmentPartInstance() method in Java creates a “part instance” in COMSOL’s “Geometry” node based on a primitive previously defined with createEnvironmentPartPrimitive(). This method just applies to building the surrounding medium. The method takes inputs, with data types and examples in parentheses: instanceID (String: “pi<#>”), instanceLabel (String:

“medium”), mediumPrimitiveString (String: Key for the medium part stored in the identifierManager), an instance of ModelWrapper, and ***Model*** as a JSON Object. Within the “medium” JSON Object in ***Model***, the parameters required to instantiate the environment part primitive are defined.

1.3.2 Part.createCuffPartInstance()

The createCuffPartInstance() method in Java is analogous to createEnvironmentPartInstance(), but it is used to instantiate cuff part geometries. We decided to separate these methods since all products of createCuffPartInstance() will be displaced and rotated by the same cuff shift (x,y,z) and rotation values.
