# Supplementary material for: ASCENT (Automated Simulations to Characterize Electrical Nerve Thresholds): A pipeline for sample-specific computational modeling of electrical stimulation of peripheral nerves
Source: PLoS Comput Biol. 2021 Sep 7;17(9):e1009285. doi: 10.1371/journal.pcbi.1009285 (PMC8423288; doi:10.1371/journal.pcbi.1009285)
Supplement: S25 Text — Control of medium surrounding nerve and cuff electrode. (PDF) [file pcbi.1009285.s025.pdf]

# 1 S25 Text

## Appendix. Control of medium surrounding nerve and cuff electrode

The medium surrounding the nerve and cuff electrode (e.g., fat, skeletal muscle) must contain a "proximal" domain, which runs the full length of the nerve, and may optionally include a "distal" domain. The parameterization for the geometry of the "proximal" and "distal" domains is shown below in Figure A. For details on how to define the "proximal" and "distal" domain geometries and meshing parameters, see model.json in S8 Text.

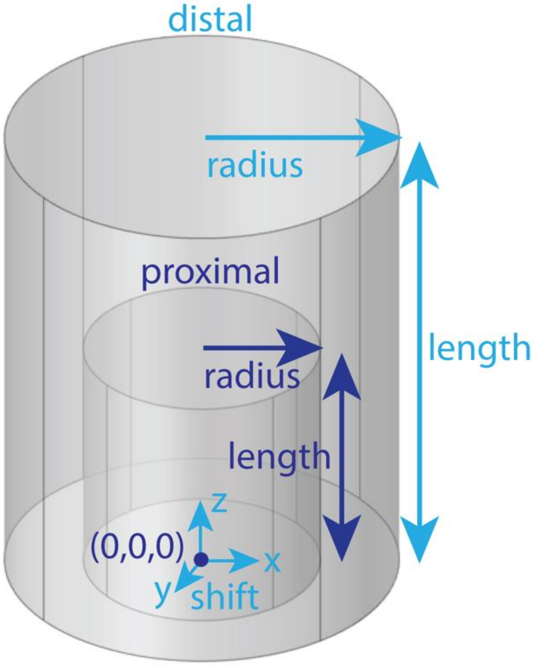


Figure A. The user must define a "proximal" domain, and may optionally define a "distal" domain for independent assignment of meshing parameters for the site of stimulation from the rest of the FEM. The "proximal" domain runs the full length of the nerve and is anchored at (0,0,0). The distal domain's radius and length may be independently assigned, and the entire distal domain may be shifted ("shift": (x,y,z)). Having a proximal domain that is overly voluminous can significantly decrease COMSOL meshing efficiency and even, rarely, cause errors. At all costs, avoid having a proximal or distal domain whose boundary intersects with a geometry (other than the nerve ends, which are at the longitudinal boundaries of the proximal domain) or the boundary of other geometries (e.g., the cuff-nerve boundary); this will likely create a meshing error.
