# Supplementary material for: ASCENT (Automated Simulations to Characterize Electrical Nerve Thresholds): A pipeline for sample-specific computational modeling of electrical stimulation of peripheral nerves
Source: PLoS Comput Biol. 2021 Sep 7;17(9):e1009285. doi: 10.1371/journal.pcbi.1009285 (PMC8423288; doi:10.1371/journal.pcbi.1009285)
Supplement: S26 Text — Java utility classes. (PDF) [file pcbi.1009285.s026.pdf]

1 S26 Text

Appendix. Java utility classes

1.1 IdentifierManager

In working with highly customized COMSOL FEMs, we found it convenient to abstract away from the underlying COMSOL indexing to improve code readability and enable scalability as models increase in geometric complexity. We developed a class named IdentifierManager that allows the user to assign their own String to identify a COMSOL geometry feature tag (e.g., wp<#>, cyl<#>, rev<#>, dif<#>) or selection (i.e., csel<#>).

We use IdentifierManagers to name and keep track of identifier labels within a PartPrimitive. IdentifierManagers assigning tags for products of individual operations to a unique “pseudonym” in HashMaps (https://docs.oracle.com/javase/8/docs/api/java/util/HashMap.html) as a key-value pair. Additionally, we assign resulting selections (“csel<#>”) to meaningful unique pseudonyms to later assign to meshes, materials, or boundary conditions.

We keep a running total for tags of IdentifierStates (i.e., the total number of uses of an individual COMSOL tag) and HashMap of IdentifierPseudonyms (i.e., a HashMap containing key (pseudonym) and value (COMSOL tag, e.g., “wp1”)).

IdentifierManager has a method next() which takes inputs of a COMSOL tag (e.g., wp, cyl, rev, dif) or selection (i.e., csel) without its index and a user’s unique pseudonym String. The method appends the next index (starting at 1) to the COMSOL tag or selection and puts the COMSOL tag or selection and associated pseudonym in the IdentifierPseudonyms HashMap. The method also updates the IdentifierStates HashMap total for the additional instance of a COMSOL tag or selection.

To later reference a COMSOL tag or selection, IdentifierManager has a get() method which takes the input of the previously assigned pseudonym key and returns the COMSOL tag or selection value from the IdentifierPseudonyms HashMap.

To accommodate mesh recycling (see ModelSearcher below), we save a COMSOL model’s IdentifierManagers to later access selections for updating model materials and physics. Therefore, we developed IdentifierManager methods toJSONObject() and fromJSONObject() which saves an IdentifierManager to a JSON file and loads an IdentifierManager into Java from a JSON Object, respectively.

1.2 JSONio

JSONio is a convenient Java class used for reading and writing JSON Objects to file. The read() method takes an input String containing the file path to read and returns a JSON Object to memory. The write() method takes an input String containing the file path for the saving destination and a JSON Object containing the data to write to file.

1.3 ModelSearcher

The ModelSearcher class in Java is used to look for previously created FEM meshed geometries that can be repurposed. For example, if ***Model*** configurations differ only in their material properties or boundary conditions and the previous ***Model's*** *.mph file with the mesh (i.e., mesh.mph) was saved, then it is redundant to build and mesh the same model geometry for a new ***Model*** configuration. The methods of the ModelSearcher class can save enormous amounts of computation time in parameter sweeps of ***Model*** if the mesh can be recycled. The user is unlikely to interface directly with this method as it operates behind the scenes, but if the user adds new parameter values to ***Model***, then the user must also add those values to config/templates/mesh_dependent_model.json to indicate whether the added parameter value needs to match between FEMs to recycle the mesh (explained further below). Generally, changes in geometry or meshing parameters need to match, but changes in material properties or boundary conditions do not, since they do not change the FEM geometry.

Specifically, this class compares ***Model*** configurations to determine if their parameters are compatible to repurpose the geometry and mesh from a previously generated COMSOL model using the meshMatch() method. The meshMatch() method takes the inputs of a reference JSON (i.e., config/templates/mesh_dependent_model.json, see S7 and S8 Text) containing conditions for compatibility and a JSON Object for each of two ***Model*** configurations to compare. The parameter keys correspond one-to-one in ***Model*** and mesh_dependent_model.json. However, in mesh_dependent_model.json, rather than numerical or categorical values for each parameter key, the keys' values are a Boolean indicating if the values between two ***Model*** configurations must be identical to define a "mesh match". For two ***Model*** configurations to be a match, all parameters assigned with the Boolean true in mesh_dependent_model.json must be identical. ***Model*** configurations that differ only in values for parameters that are assigned the Boolean false are considered a mesh match and do not require that the geometry be re-meshed.

In the class's searchMeshMatch() method, the program looks through all ***Model*** configurations under a given ***Sample*** and applies the meshMatch() method. If a ***Model*** match is found, searchMeshMatch returns a Match class, which is analogous to the ModelWrapper class, using the path of the matching ***Model*** with the fromMeshPath() method.
