# Supplementary material for: ASCENT (Automated Simulations to Characterize Electrical Nerve Thresholds): A pipeline for sample-specific computational modeling of electrical stimulation of peripheral nerves
Source: PLoS Comput Biol. 2021 Sep 7;17(9):e1009285. doi: 10.1371/journal.pcbi.1009285 (PMC8423288; doi:10.1371/journal.pcbi.1009285)
Supplement: S27 Text — Defining and assigning materials in COMSOL. (PDF) [file pcbi.1009285.s027.pdf]

1 S27 Text

Appendix. Defining and assigning materials in COMSOL

Materials are defined in the COMSOL “Materials” node for each material “function” indicated in the “preset” cuff configuration file (i.e., cuff “insulator”, contact “conductor”, contact “recess”, and cuff “fill”) and nerve domain (i.e., endoneurium, perineurium, epineurium). Material properties for each function are assigned in ***Model*’s** “conductivities” JSON Object by either referencing materials in the default materials library (config/system/materials.json) by name, or with explicit definitions of a materials name and conductivity as a JSON Object (S8 Text).

1.1 ModelWrapper.addMaterialDefinitions()

The user is unlikely to interface directly with the addMaterialDefinitions() method in Java as it operates behind the scenes. The method takes an input of a list of strings containing the material functions (i.e., endoneurium, perineurium, epineurium, cuff “fill”, cuff “insulator”, contact “conductor”, and contact “recess”), ***Model***, and a COMSOL ModelParamGroup for containing the material properties as a COMSOL “Parameters Group” under COMSOL’s “Global Definitions”. The method then loops over all material functions, creates a new material if it does not yet exist as “mat<#>” using Part’s defineMaterial() method, and adds the identifier (e.g., “mat1”) to the IdentifierManager.

1.2 Part.defineMaterial()

The user is unlikely to interface directly with the defineMaterial() method in Java as it operates behind the scenes to add a new material to the COMSOL “Materials” node under “Global Definitions”. The method takes inputs of the material’s identifier in COMSOL (e.g., “mat1”), function (e.g., cuff “fill”), ***Model***, a library of predefined materials (e.g., materials.json), a ModelWrapper instance, and the COMSOL ModelParamGroup for material conductivities. The defineMaterial() method uses materials present in ***Model’s*** “conductivities” JSON Object to assign to each material function in the COMSOL model (e.g., insulator, conductor, fill, endoneurium, perineurium, epineurium, or medium). The material value for a function key in ***Model*** is either a string for a pre-defined material in materials.json, or a JSON Object (containing unit, label, and value) for a custom material. Assigning material conductivities to material functions in this manner enables control for a user to reference a pre-defined material or explicitly link a custom material to a COMSOL model. In either the case of a predefined material in materials.json or custom material in ***Model***, if the material is anisotropic, the material value is assigned a string “anisotropic” which tells the program to look for independent “sigma_x”, “sigma_y”, and “sigma_z” values in the material JSON Object.

1.3 ModelWrapper.addCuffPartMaterialAssignment()

The user is unlikely to interface directly with the addCuffPartMaterialAssignment() method in Java as it operates behind the scenes. The method loads in a cuff part primitive's labels from its IdentifierManager. The method loops over the list of material JSON Objects in the "preset" cuff configuration file. For each material function in the "preset" cuff configuration file, the method creates a COMSOL Material Link to assign a previously defined selection in a cuff part instance to a defined material.

1.4 ModelWrapper.addCuffPartMaterialAssignments()

The user is unlikely to interface directly with the addCuffMaterialAssignments() method in Java as it operates behind the scenes. The method loops through all part instances in the cuff configuration file, which is linked to ***Model*** by a string of its file name under the "preset" key, and calls the addCuffMaterialAssignment() method for each part instance. As described in addCuffPartMaterialAssignment() above, a material is connected to the selection within the part primitive by its label. In COMSOL, material links appear in the "Materials" node. COMSOL assigns the material links in the order of the part instances defined in the "preset" cuff configuration file, which is important since material links overwrite previous domain assignments. For this reason, it is important to list part instances in "preset" cuff files in a nested order (i.e., the outermost domains first, knowing that domains nested in space within them will overwrite earlier domain assignments).

1.5 Adding and assigning default material properties

Default material properties defined in config/system/materials.json are listed in Table A. To accommodate automation of frequency-dependent material properties (for a single frequency, i.e., sinusoid), parameters for material conductivity that are dependent on the stimulation frequency are calculated in Runner's compute_electrical_parameters() method and saved to ***Model*** before the handoff() method is called. Our pipeline supports calculation of the frequency-dependent conductivity of the perineurium based on measurements from the frog sciatic nerve [1] using the rho_weerasuriya() method in the Python Waveform class. See Fig 2 for identification of tissue types in a compound nerve cross section (i.e., epineurium, perineurium, endoneurium).

Table A. Default material conductivities.

Material	Conductivity	References
silicone	10^-12 [S/m]	[2]
platinum	9.43 × 10^6 [S/m]	[3]
endoneurium	{1/6, 1/6, 1/1.75} [S/m]	[4,5]

epineurium	1/6.3 [S/m]	[6–8]
muscle	{0.086, 0.086, 0.35} [S/m]	[9]
fat	1/30 [S/m]	[10]
encapsulation	1/6.3 [S/m]	[7]
saline	1.76 [S/m]	[11]
perineurium	1/1149 [S/m]	[1,5]

1.6 References

1. Weerasuriya A, Spangler RA, Rapoport SI, Taylor RE. AC impedance of the perineurium of the frog sciatic nerve. Biophys J. 1984 Aug;46(2):167–74. Available from: https://doi.org/10.1016/s0006-3495(84)84009-6 PMID: 6332648
2. Callister WD, Rethwisch DG. Fundamentals of Material Science and Engineering An Integrated Approach. In: Fundamentals Of Material Science and Engineering An Intergrated Approach. 2012.
3. de Podesta M, Laboratory NP, UK. Understanding the Properties of Matter. Understanding the Properties of Matter. 1996.
4. Ranck JB, BeMent SL. The specific impedance of the dorsal columns of cat: An anisotropic medium. Exp Neurol [Internet]. 1965 Apr 1 [cited 2020 Apr 20];11(4):451–63. Available from: https://doi.org/10.1016/0014-4886(65)90059-2 PMID: 14278100
5. Pelot NA, Behrend CE, Grill WM. On the parameters used in finite element modeling of compound peripheral nerves. J Neural Eng [Internet]. 2019;16(1):16007. Available from: http://dx.doi.org/10.1088/1741-2552/aaeb0c PMID: 30507555
6. Stolinski C. Structure and composition of the outer connective tissue sheaths of peripheral nerve. J Anat [Internet]. 1995 Feb;186(1):123–30. Available from: https://pubmed.ncbi.nlm.nih.gov/7649808 PMID: 7649808
7. Grill WM, Mortimer TJ. Electrical properties of implant encapsulation tissue. Ann Biomed Eng [Internet]. 1994;22(1):23–33. Available from: https://doi.org/10.1007/BF02368219 PMID: 8060024
8. Pelot NA, Behrend CE, Grill WM. Modeling the response of small myelinated axons in a compound nerve to kilohertz frequency signals. J Neural Eng. 2017 Aug;14(4):46022. Available from: https://doi.org/10.1088/1741-2552/aa6a5f PMID: 28361793
9. Gielen FLH, Wallinga-de Jonge W, Boon KL. Electrical conductivity of skeletal muscle tissue: Experimental results from different musclesin vivo. Med Biol Eng Comput [Internet]. 1984;22(6):569–77. Available from: https://doi.org/10.1007/BF02443872 PMID: 6503387
10. Geddes LA, Baker LE. The specific resistance of biological material—A compendium of data for the biomedical engineer and physiologist. Med Biol Eng [Internet]. 1967;5(3):271–93. Available from: https://doi.org/10.1007/BF02474537 PMID: 6068939
11. Horch K. Neuroprosthetics: Theory and practice: Second edition. Neuroprosthetics: Theory and Practice: Second Edition. 2017. 1–925 p.
