# Supplementary material for: ASCENT (Automated Simulations to Characterize Electrical Nerve Thresholds): A pipeline for sample-specific computational modeling of electrical stimulation of peripheral nerves
Source: PLoS Comput Biol. 2021 Sep 7;17(9):e1009285. doi: 10.1371/journal.pcbi.1009285 (PMC8423288; doi:10.1371/journal.pcbi.1009285)
Supplement: S28 Text — Definition of perineurium. (PDF) [file pcbi.1009285.s028.pdf]

1 S28 Text

Appendix. Definition of perineurium

1.1 Definition of perineurium

The perineurium is a thin highly resistive layer of connective tissue and has a profound impact on thresholds of activation and block. Our previous modeling work demonstrates that representing the perineurium with a thin layer approximation (Rm = rho*peri_thk), rather than as a thinly meshed domain, reduces mesh complexity and is a reasonable approximation [1]. Therefore, perineurium can be modeled with a thin layer approximation (except with “peanut” fascicles; see an example in Fig 2), termed “contact impedance” in COMSOL (if ***Model’s*** “use_ci” parameter is true (S8 Text)), which relates the normal component of the current density through the surface ($\vec{n} \cdot \vec{J_1}$) to the drop in electric potentials ($V_1 - V_2$) and the sheet resistance (ρ_s):

$$\vec{n} \cdot \vec{J_1} = \frac{1}{\rho_s}(V_1 - V_2)$$

The sheet resistance (ρ_s) is defined as the sheet thickness (d_s) divided by the material bulk conductivity (σ_s):

$$\rho_s = \frac{d_s}{\sigma_s}$$

Our previously published work quantified the relationship between fascicle diameter and perineurium thickness [2] (Table A).

Table A. Previously published relationships between fascicle diameter and perineurium thickness.

Species	peri_thk: f(species, d_{fasc})	References
Rat	peri_thk = 0.01292*d_{fasc} + 1.367 [um]	[2]
Pig	peri_thk = 0.02547*d_{fasc} + 3.440 [um]	[2]
Human	peri_thk = 0.03702*d_{fasc} + 10.50 [um]	[2]

The “rho_perineurium” parameter in ***Model*** can take either of two modes:

- “RHO_WEERASURIYA”: The perineurium conductivity value changes with the frequency of electrical stimulation (for a single value, not a spectrum, defined in ***Model*** as “frequency”) and temperature (using a Q10 adjustment, defined in ***Model*** as “temperature”) based on measurements of frog sciatic perineurium [1,3]. The equation is

defined in src/core/Waveform.py in the rho_weerasuriya() method.

- “MANUAL”: Conductivity value assigned to the perineurium is as explicitly defined in either materials.json or ***Model*** without any corrections for temperature or frequency.

1.2 References

1. Pelot NA, Behrend CE, Grill WM. On the parameters used in finite element modeling of compound peripheral nerves. J Neural Eng [Internet]. 2019;16(1):16007. Available from: http://dx.doi.org/10.1088/1741-2552/aaeb0c PMID: 30507555
2. Pelot NA, Goldhagen GB, Cariello JE, Musselman ED, Clissold KA, Ezzell JA, et al. Quantified Morphology of the Cervical and Subdiaphragmatic Vagus Nerves of Human, Pig, and Rat. Front Neurosci [Internet]. 2020;14:1148. Available from: https://doi.org/10.3389/fnins.2020.601479 PMID: 33250710
3. Weerasuriya A, Spangler RA, Rapoport SI, Taylor RE. AC impedance of the perineurium of the frog sciatic nerve. Biophys J. 1984 Aug;46(2):167–74. Available from: https://doi.org/10.1016/s0006-3495(84)84009-6 PMID: 6332648
