# Supplementary material for: ASCENT (Automated Simulations to Characterize Electrical Nerve Thresholds): A pipeline for sample-specific computational modeling of electrical stimulation of peripheral nerves
Source: PLoS Comput Biol. 2021 Sep 7;17(9):e1009285. doi: 10.1371/journal.pcbi.1009285 (PMC8423288; doi:10.1371/journal.pcbi.1009285)
Supplement: S29 Text — Data interchange between COMSOL and NEURON. (PDF) [file pcbi.1009285.s029.pdf]

1 S29

Appendix. Data interchange between COMSOL and NEURON

The user is unlikely to interface directly with ModelWrapper's extractAllPotentials() method in Java as it operates behind the scenes. The method takes input arguments for the project path and a run path. Using the run path, the method loads ***Run***, and constructs lists of ***Model*** and ***Sim*** for which it will call extractPotentials() for each fiberset. COMSOL is expecting a $(3 \times n)$ matrix of coordinates (Double[3][n]), defining the (x,y,z)-coordinates for each of n points.

The Java COMSOL API methods setInterpolationCoordinates() and getData() for a model object are fast compared to the time for a machine to load a COMSOL "model" object to memory from file. Therefore, the extractAllPotentials() method is intentionally configured to minimize the number of times a "basis" COMSOL "model" object is loaded into memory. We accomplish this by looping in the following order: ***Model***, bases, ***Sims***, fibersets (i.e., groups of fibers with identical geometry/channels, but different (x,y)-locations and/or longitudinal offsets), then fibers. With this approach, we load each COMSOL "model" object only once (i.e., *.mph members of bases/). Within the loop, the extractPotentials() method constructs the bases (double[basis index][sim index][fiberset index][fiber index]) for each model (units: Volts). With the bases in memory, the program constructs the potentials for inputs to NEURON by combining bases by their contact weights and writes them to file within potentials/ (or ss_bases/), which mirrors fibersets/ (or ss_coords/) in contents.
