# Supplementary material for: ASCENT (Automated Simulations to Characterize Electrical Nerve Thresholds): A pipeline for sample-specific computational modeling of electrical stimulation of peripheral nerves
Source: PLoS Comput Biol. 2021 Sep 7;17(9):e1009285. doi: 10.1371/journal.pcbi.1009285 (PMC8423288; doi:10.1371/journal.pcbi.1009285)
Supplement: S30 Text — Python Simulation class. (PDF) [file pcbi.1009285.s030.pdf]

1 S30 Text

Appendix. Python Simulation class

1.1 (Pre-Java)

The user is unlikely to interface directly with Simulation's resolve_factors() method as it operates behind the scenes. The method searches through ***Sim*** for lists of parameters within the "fibers" and "waveform" JSON Objects until the indicated number of dimensions ("n_dimensions" parameter in ***Sim***, which is a handshake to prevent erroneous generation of NEURON simulations) has been reached. The parameters over which the user has indicated to sweep in ***Sim*** are saved to the Simulation class as a dictionary named "factors" with the path to each parameter in ***Sim***.

The required parameters to define each type of waveform are in S8 Text. The Python Waveform class is configured with ***Sim,*** which contains all parameters that define the Waveform. Since FEMs may have frequency-dependent conductivities, the parameter for frequency of stimulation is optionally defined in ***Model*** (for frequency-dependent material conductivities), but the pulse repetition frequency is defined in ***Sim*** as "pulse_repetition_freq". The write_waveforms() method instantiates a Python Waveform class for each "wave_set" (i.e., one combination of stimulation parameters).

1.2 (Post-Java)

The unique combinations of ***Sim*** parameters are found with a Cartesian product from the listed values for individual parameters in ***Sim***: Waveforms × Src_weights × Fibersets. The pipeline manages the indexing of simulations. For ease of debugging and inspection, into each n_sim/ directory we copy in a modified "reduced" version of ***Sim*** with any lists of parameters replaced by the single list element value investigated in the particular n_sim/ directory.

The Simulation class loops over ***Model*** and ***Sim*** as listed in ***Run*** and loads the Python "sim.obj" object saved in each simulation directory (sims/<sim index>/) prior to Python's handoff() to Java. Using the Python object for the simulation loaded into memory, the Simulation class's method build_n_sims() loops over the master_product_index (i.e., waveforms × (src_weights × fibersets)). For each master_product_index, the program creates the n_sim file structure (sims/<sim index>/n_sims/<n_sim index>/data/inputs/ and sims/<sim index>/n_sims/<n_sim index>/data/outputs/). Corresponding to the n_sim's master_product_index, files are copied into the n_sim directory for a "reduced" ***Sim***, stimulation waveform, and fiber potentials. Additionally, the program writes a HOC file (i.e., "launch.hoc") containing parameters for and a call to our Wrapper.hoc file using the Python HocWriter class.

To conveniently submit the n_sim directories to a computer cluster, we created methods within Simulation named export_n_sims(), export_run(), and export_neuron_files(). The method export_n_sims() copies n_sims from our native hierarchical file structure to a target directory as defined in the system env.json config file by the value for the "ASCENT_NSIM_EXPORT_PATH" key. Within the target directory, a directory named n_sims/ contains all n_sims. Each n_sim is renamed corresponding to its sample, model, sim, and master_product_index (<sample_index>_<model_index>_<sim_index>_<master_product_index>) and is therefore unique. Analogously, export_run() creates a copy of **Run** within the target directory in a directory named runs/. Lastly, export_neuron_files() is used to create a copy of the NEURON *.hoc and *.mod files in the target directory in directories named "HOC_Files" and "MOD_Files", respectively.
