# Supplementary material for: ASCENT (Automated Simulations to Characterize Electrical Nerve Thresholds): A pipeline for sample-specific computational modeling of electrical stimulation of peripheral nerves
Source: PLoS Comput Biol. 2021 Sep 7;17(9):e1009285. doi: 10.1371/journal.pcbi.1009285 (PMC8423288; doi:10.1371/journal.pcbi.1009285)
Supplement: S31 Text — NEURON launch.hoc. (PDF) [file pcbi.1009285.s031.pdf]

1 S31 Text

Appendix. NEURON launch.hoc

The launch.hoc file defines the parameters and simulation protocol for modeling fiber response to electrical stimulation in NEURON and is automatically populated based on parameters in ***Model*** and ***Sim***. The launch.hoc file is created by the HocWriter class. Parameters defined in launch.hoc span the categories of: environment (i.e., temperature from ***Model***), simulation time (i.e., time step, duration of simulation from ***Sim***), fiber parameters (i.e., flags for fiber geometry and channels, number of fiber nodes from ***Model***, ***Sim***, and config/system/fiber_z.json), intracellular stimulation (i.e., delay from start of simulation, amplitude, pulse duration, pulse repetition frequency from ***Sim***), extracellular stimulation (i.e., path to waveform file in n_sim/ folder which is always data/inputs/waveform.dat), flags to define the model parameters that should be recorded (i.e., Vm(t), Gating(t), Vm(x), Gating(x) from ***Sim***), the locations at which to record the parameters (nodes of Ranvier for myelinated axons from ***Sim***), and parameters for the binary search for thresholds (i.e., activation or block protocol, initial upper and lower bounds on the stimulation amplitude for the binary search, and threshold resolution for the binary search from ***Sim***). The launch.hoc file loads Wrapper.hoc which calls all NEURON procedures. The launch.hoc file is created by the Python HocWriter class, which takes inputs of the ***Sim*** directory, n_sim directory, and an exception configuration. When the HocWriter class is instantiated, it automatically loads the fiber_z.json configuration file which contains all associated flags, parameters, and rules for defining a fiber's geometry and channel mechanisms in NEURON.
