# Supplementary material for: ASCENT (Automated Simulations to Characterize Electrical Nerve Thresholds): A pipeline for sample-specific computational modeling of electrical stimulation of peripheral nerves
Source: PLoS Comput Biol. 2021 Sep 7;17(9):e1009285. doi: 10.1371/journal.pcbi.1009285 (PMC8423288; doi:10.1371/journal.pcbi.1009285)
Supplement: S32 Text — NEURON Wrapper.hoc. (PDF) [file pcbi.1009285.s032.pdf]

1 S32 Text

Appendix. NEURON Wrapper.hoc

The Wrapper.hoc file coordinates all program operations to create a biophysically realistic discrete cable fiber model, simulate the fiber's response to extracellular and intracellular stimulation, and record the response of the fiber. For each fiber simulated in NEURON, outputs are saved to <n_sim_index>/data/outputs/. For simulations running an activation or block threshold protocol, data outputs include threshold current amplitudes. For simulation of fiber response to set amplitudes, the user may save state variables at each compartment in NEURON to file at discrete times and/or locations.

1.1 Create fiber model

Based on the flag for "fiber_type" set in launch.hoc (associated by a fiber type parameter in fiber_z.json and FiberGeometryMode (S8 Text)), Wrapper.hoc loads the corresponding template for defining fiber geometry discretization, i.e., "GeometryBuilder.hoc" for myelinated fibers and "cFiberBuilder.hoc" for unmyelinated fibers. For all fiber types, the segments created and connected in NEURON have lengths that correspond to the coordinates of the input potentials.

1.2 Intracellular stimulus

For simulations of block threshold, an intracellular test pulse is delivered at one end of the fiber to test if the cuff electrode (i.e., placed between the intracellular stimulus and the site of detecting action potentials) is blocking action potentials (S22 Text). The intracellular stimulation parameters are defined in ***Sim*** and are defined as parameters in NEURON within the launch.hoc file. The parameters in ***Sim*** control the pulse delay, pulse width, pulse repetition frequency, pulse amplitude, and node/section index of the intracellular stimulus (S8 Text). For simulating activation thresholds, the intracellular stimulation amplitude should be set to zero.

1.3 Extracellular stimulus

To simulate response of individual fibers to electrical stimulation, we use NEURON's extracellular mechanisms to apply the electric potential from COMSOL at each segment of the cable model as a time-varying signal. We load in the stimulation waveform from a n_sim's data/inputs/ directory using the VeTime_read() procedure within ExtracellularStim_Time.hoc. The saved stimulation waveform is unscaled, meaning the maximum current magnitude at any timestep is +/-1. Analogously, we read in the potentials for the fiber being simulated from data/inputs/ using the VeSpace_read() procedure within ExtracellularStim_Space.hoc.

1.4 Recording

The NEURON simulation code contains functionality ready to record and save to file the values of state variables at discrete spatial locations for all times and/or at discrete times for all spatial locations (i.e., nodes of Ranvier for myelinated fibers or sections for unmyelinated fibers) for applied extracellular potential, intracellular stimulation amplitude, transmembrane potential, and gating parameters using Recording.hoc. The recording tools are particularly useful for generating data to troubleshoot and visualize simulations.

1.5 RunSim

Our procedure RunSim is responsible for simulating the response of the model fiber to intracellular and extracellular stimulation. Before the simulation starts, the procedure adds action potential counters to look for a rise above a threshold transmembrane potential.

So that each fiber reaches a steady-state before the simulation starts, the RunSim procedure initializes the fiber by stepping through large time steps with no extracellular potential applied to each compartment. RunSim then loops over each time step, and, while updating the value of extracellular potential at each fiber segment, records the values of flagged state variables as necessary.

At the end of RunSim's loop over all time steps, if the user is searching for threshold current amplitudes, the method evaluates if the extracellular stimulation amplitude was above or below threshold, as indicated by the presence or absence of an action potential for activation and block thresholds, respectively.

1.6 FindThresh

The procedure FindThresh performs a binary search for activation and block thresholds (S22 Text).

1.7 Save outputs to file

At the end of the NEURON simulation, the program saves state variables as indicated with saveflags, CPU time, and threshold values. Output files are saved to the data/outputs/ directory within its n_sim folder.
