# Supplementary material for: ASCENT (Automated Simulations to Characterize Electrical Nerve Thresholds): A pipeline for sample-specific computational modeling of electrical stimulation of peripheral nerves
Source: PLoS Comput Biol. 2021 Sep 7;17(9):e1009285. doi: 10.1371/journal.pcbi.1009285 (PMC8423288; doi:10.1371/journal.pcbi.1009285)
Supplement: S33 Text — Data analysis tools. (PDF) [file pcbi.1009285.s033.pdf]

1 S33 Text

Appendix. Data analysis tools

1.1 Python Query class

The general usage of Query is as follows:

1. In the context of a Python script, the user specifies the search criteria (think of these as "keywords" that filter your data) in the form of a JSON configuration file (see query_criteria.json in S8 Text).
2. These search criteria are used to construct a Query object, and the search for matching ***Sample***, ***Model***, and ***Sim*** configurations is performed using the method run().
3. The search results are in the form of a hierarchy of ***Sample***, ***Model***, and ***Sim*** indices, which can be accessed using the summary() method.

Using this "summary" of results, the user is then able to use various convenience methods provided by the Query class to build paths to arbitrary points in the data file structure as well as load saved Python objects (e.g., Sample and Simulation class instances).

The Query class's initializer takes one argument: a dictionary in the appropriate structure for query criteria (S8 Text) *or* a string value containing the path (relative to the pipeline repository root) to the desired JSON configuration with the criteria. Put concisely, a user may filter results either manually by using known indices or automatically by using parameters as they would be found in the main configuration files. It is ***extremely important*** to note that the Query class must be initialized with the working directory set to the root of the pipeline repository (i.e., sys.path.append(ASCENT_PROJECT_PATH) in your script). Failure to set the working directory correctly will break the initialization step of the Query class.

After initialization, the search can be performed by calling Query's run() method. This method recursively dives into the data file structure of the pipeline searching for configurations (i.e., ***Sample***, ***Model***, and/or ***Sim***) that satisfy query_criteria.json. Once run() has been called, the results can be fetched using the summary() accessor method. In addition, the user may pass in a file path to excel_output() to generate an Excel sheet summarizing the Query results.

Query also has methods for accessing configurations and Python objects within the samples/ directory based on a list of ***Sample***, ***Model***, or ***Sim*** indices. The build_path() method returns the path of the configuration or object for the provided indices. Similarly, the get_config() and get_object() methods return the configuration dictionary or saved Python object (using the Pickle package), respectively, for a list of configuration indices. These tools allow for convenient looping through the data associated with search criteria.

In addition, we have included a few data analysis methods in the Query class: heatmaps(), barcharts_compare_models(), and barcharts_compare_samples(). Since individual use cases for data analysis can differ greatly, these methods are not considered “core” functionality and can instead be treated as examples for how one might use the Query class. Example uses of these Query convenience methods are included in examples/analysis/.

- plot_sample.py
- plot_fiberset.py
- plot_waveform.py

1.2 Video generation for NEURON state variables

In examples/analysis/ we provide a script, plot_video.py, that creates an animation of saved state variables as a function of space and time (e.g., transmembrane potentials, MRG gating parameters). The user can plot n_sim data saved in a data/output/ folder by referencing indices for ***Sample***, ***Model***, ***Sim***, inner, fiber, and n_sim. The user may save the animation as either a *.mp4 or *.gif file to the data/output/ folder.

The plot_video.py script is useful for determining necessary simulation durations (e.g., the time required for an action potential to propagate the length of the fiber) to avoid running unnecessarily long simulations. Furthermore, the script is useful for observing onset response to kilohertz frequency block, which is important for determining the appropriate duration of time to allow for the fiber onset response to complete.

Users need to determine an appropriate number of points along the fiber to record state variables. Users have the option to either record state variables at all Nodes of Ranvier (myelinated fibers) or sections (unmyelinated fibers), or at discrete locations along the length of the fiber (S8 Text).
