# Supplementary material for: ASCENT (Automated Simulations to Characterize Electrical Nerve Thresholds): A pipeline for sample-specific computational modeling of electrical stimulation of peripheral nerves
Source: PLoS Comput Biol. 2021 Sep 7;17(9):e1009285. doi: 10.1371/journal.pcbi.1009285 (PMC8423288; doi:10.1371/journal.pcbi.1009285)
Supplement: S34 Text — Convergence analysis example*. (PDF) [file pcbi.1009285.s034.pdf]

1 S34 Text

Appendix. Convergence analysis example*

1.1 Convergence analysis example*

*Note: The following convergence analysis was performed for a rat cervical vagus nerve and is provided as an illustrative example.

The outer boundaries of the model (assigned to electrical ground) were initially set to dimensions that are known to be excessively large in both length (100 mm) and radius (20 mm) (Figure A). For testing of convergence of the model length and diameter, we carefully controlled the mesh parameters such that any changes in the threshold to excite model nerve fibers positioned in the finite element model are attributed to changes in model dimensions and not to a change in mesh density. First, we used an excessive number of domain mesh elements. Second, to maintain consistent meshing in the area of interest despite a change in geometry of the outer boundary of the model, the proximal region of the model surrounding the nerve and electrode (i.e., “proximal”, which is the full length of the nerve) was assigned its own mesh entirely independent from the outer boundary region (i.e., “distal”).

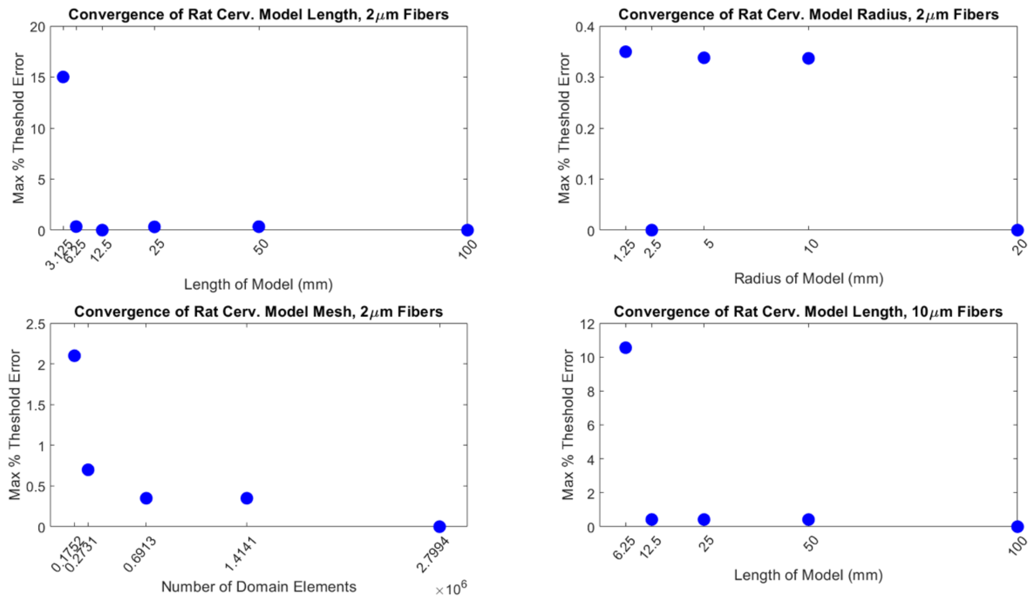

Figure A. Convergence of FEM model dimensions and mesh density for 2 and 10 μm myelinated MRG fibers modeled in a rat cervical vagus nerve.

For different model sizes and mesh densities, we determined excitation thresholds for 100 randomly placed 2 μm diameter myelinated model axons in the nerve. First, with the radius of the model held constant at 20 mm, we halved the length of the model until there was a change in

threshold of greater than 2% compared to the 100 mm-long model. The resulting length of the model was chosen to be 6.25 mm, as the next shortest length (3.125 mm) had at least one fiber with a threshold change of 15%, which was above our tolerance of 2%. Second, with the length of the model held constant at 6.25 mm, the radius of the model was halved until there was a change in threshold of greater than 2% compared to the 20 mm-radius model. The resulting radius of the model was chosen to be 1.25 mm. The radius of the model could not be reduced further without interfering with the electrode geometry. We then reduced the number of mesh elements until there was a 2% change in thresholds for activation of model nerve fibers. Meshing parameters that resulted in ~273,000 domain elements for the 1.25 mm radius and 6.25 mm length model were chosen for further studies.

Lastly, the length was again converged with 10 µm diameter myelinated model axons using the previously found model radius and mesh density to ensure that the model length incorporated enough nodes of Ranvier for convergence of threshold for all native fiber diameters. A conservative estimate for the largest myelinated fiber diameter found in the cervical level of a rat vagus nerve is 10 µm [1]. The largest diameter nerve fiber native to this region is of interest in our convergence studies because it will have the fewest number of nodes per unit length (based on INL=100*D). We found that the FEM length needed to be longer for 10 µm diameter myelinated fibers (12.5 mm) than 2 µm diameter myelinated fibers (6.25 mm).

1.2 References

1. Licursi de Alcântara AC, Salgado HC, Sassoli Fazan VP. Morphology and morphometry of the vagus nerve in male and female spontaneously hypertensive rats. Brain Res. 2008 Mar;1197:170–80. Available from: https://doi.org/10.1016/j.brainres.2007.12.045 PMID: 18234157
