# Supplementary material for: ASCENT (Automated Simulations to Characterize Electrical Nerve Thresholds): A pipeline for sample-specific computational modeling of electrical stimulation of peripheral nerves
Source: PLoS Comput Biol. 2021 Sep 7;17(9):e1009285. doi: 10.1371/journal.pcbi.1009285 (PMC8423288; doi:10.1371/journal.pcbi.1009285)
Supplement: S35 Text — Sim4Life validation. (PDF) [file pcbi.1009285.s035.pdf]

1 S35 Text

Appendix. Sim4Life validation

We designed test simulations to verify ASCENT's activation thresholds. The verifications were performed by The Foundation for Research Technologies in Society (IT'IS) with the Sim4Life (https://zmt.swiss/sim4life/) (Zurich, Switzerland) simulation platform. Running the following simulations required modification of the Sim4Life solver to implement the required electrical anisotropy of tissue conductivities and the boundary condition to represent the thin layer approximation used to model the perineurium (S28 Text).

1.1 Monofascicular rat nerve model

We validated activation thresholds for fibers seeded in a model of a rat cervical vagus nerve instrumented with a bipolar cuff electrode (Figure A and B).

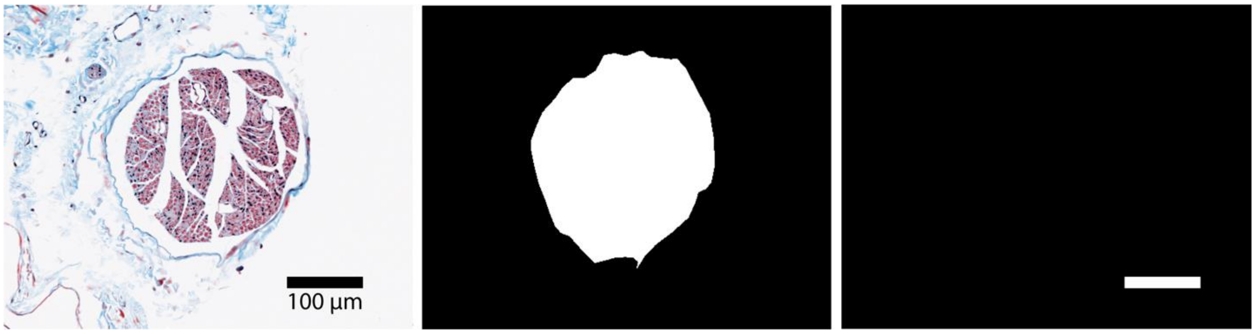

Figure A. Raw histology image (r.tif), segmented histology (i.tif), and scalebar (s.tif) of a rat cervical vagus nerve sample that served as inputs to define the cross section of the nerve in the FEM.

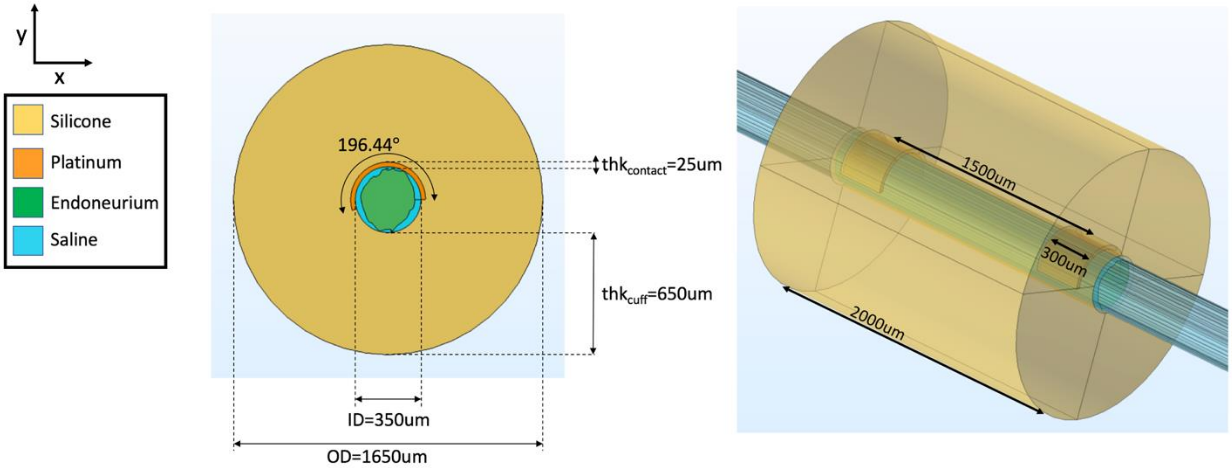

Figure B. FEM of a rat cervical vagus nerve sample instrumented with a bipolar cuff electrode.

The conductivity values applied to the rat cervical FEM are provided in Table A, and the boundary conditions applied are provided in Table B.

Table A. Conductivity values for FEM of rat cervical vagus nerve. These values were also used in multifascicular nerve model and human model verifications.

**Parameter**	**Application**	**Resistivity** Ω-m
Endoneurium	Within each fascicle	1.75 longitudinal<br>6 radial (rat and human)<br>12 radial (multifascicular dummy model)
Saline	Cylindrical shell between the nerve and cuff	1/1.76
Platinum	For both contacts	$1/(9.43*10^6)$
Silicone	For electrode body	10^{12}
Muscle	Used for the surrounding "medium": Everything outside of the nerve and cuff, other than the saline layer between the nerve and cuff	1/0.35 longitudinal<br>1/0.086 radial
Epineurium	Within the nerve around each fascicle	6.3
Encapsulation	Between cuff and nerve, and immediately surrounding cuff	6.3

Table B. Boundary conditions used in FEM of rat cervical vagus nerve, multifascicular dummy nerve, and human cervical vagus nerve.

Parameter	Setting
Current conservation	All domains
Initial condition	V=0 (all domains)
Perineurium (sides of each fascicle)	Modeled as contact impedance (S28 Text)<br>1149 Ω-m * 0.03 * d_{fasc}[m]
Ground (all outer boundaries of the model)	V = 0
Point current source (one in each contact)	-1 mA<br>1 mA

We compared thresholds for 100 5.7 µm myelinated axons (MRG model) seeded in the cross section of the nerve in response to a single 100 µs duration monophasic rectangular pulse. The differences in thresholds between ASCENT and IT'IS model implementations was <4.2% for all fibers, demonstrating strong agreement (Figure C).

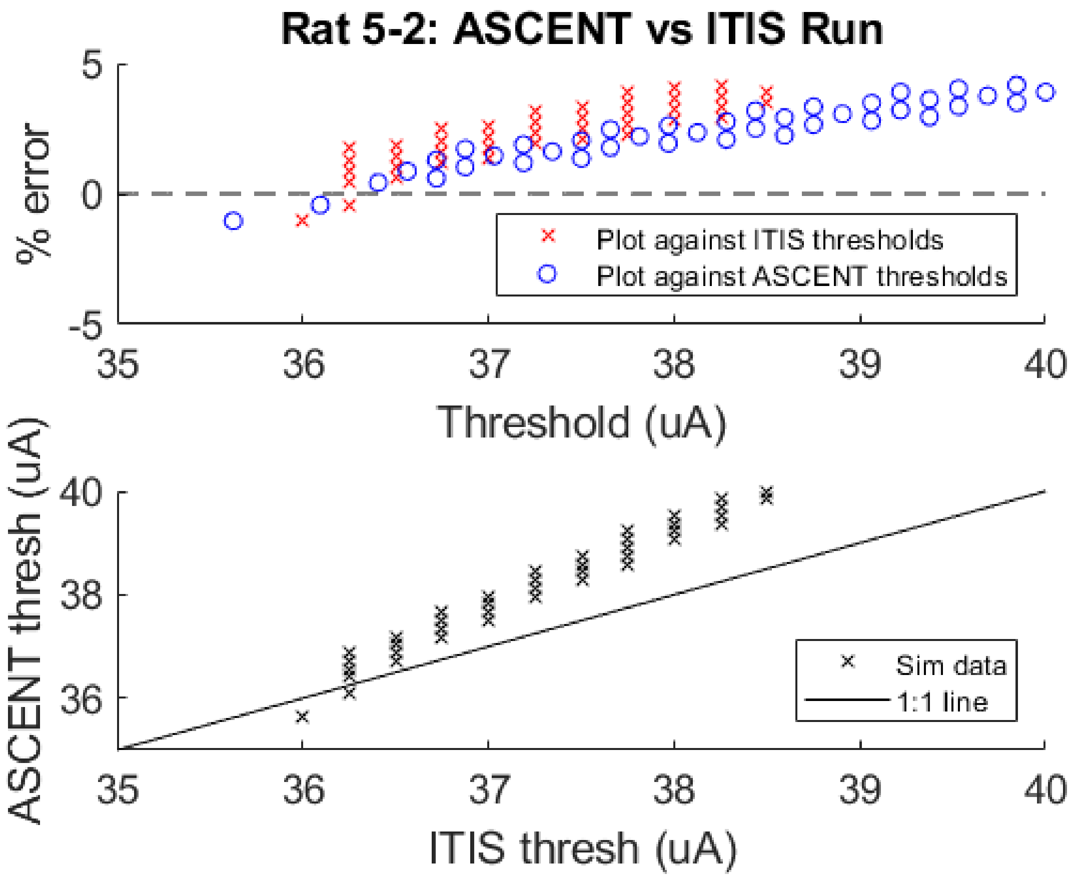

Figure C. Comparison of activation thresholds for the rat cervical vagus nerve implementation in ASCENT and Sim4Life.

1.2 Multifascicular Dummy Nerve Model

We validated activation thresholds for fibers seeded in a multifascicular dummy nerve instrumented with a bipolar cuff electrode (Figure D and E). The segmented histology was created using our mock_morphology_generator.py script (S12 Text).

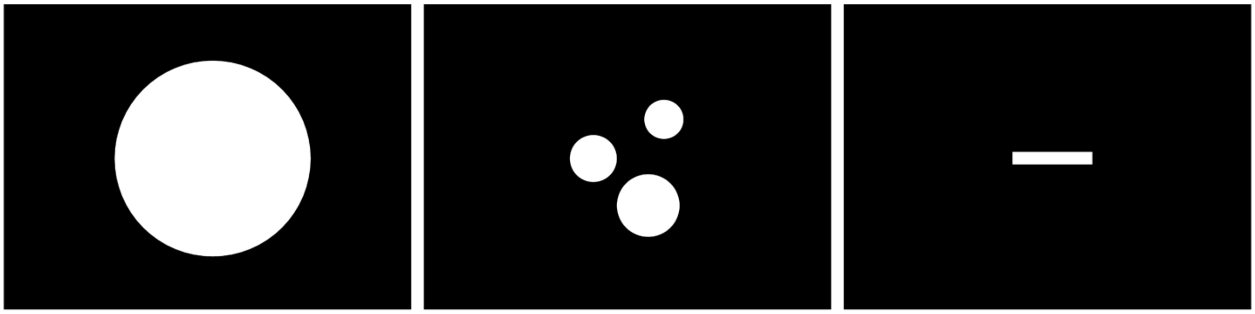

Figure D. Mock morphology inputs to the define tissue boundaries for a multifascicular dummy nerve. Scale bar is 100 µm long. The nerve is a perfect circle (diameter = 250 µm, centered at (x,y)=(0,0) µm). The inners are also perfect circles: (1) diameter = 50 µm, centered at (x,y)=(40,50) µm, (2) diameter = 60 µm, centered at (x,y)=(-50,0) µm, and (3) diameter = 80 µm, centered at (x,y)=(20,-60) µm.

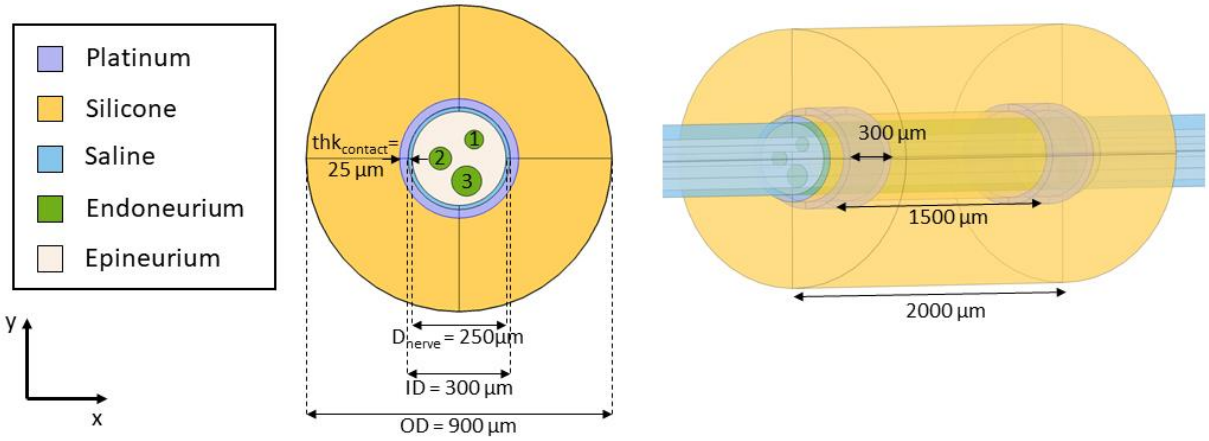

Figure E. FEM of a multifascicular nerve sample instrumented with a bipolar cuff electrode.

The conductivity values applied to multifascicular nerve sample finite element model are provided in Table A, and the boundary conditions applied are provided in Table B.

We seeded a single 5.7 µm diameter fiber in the center of each fascicle. Between the ASCENT and IT'IS implementations, there was less than a 3% difference in threshold to a single 100 µs duration monophasic rectangular pulse.

1.3 Multifascicular Human Nerve Model

We validated activation thresholds for fibers seeded in a multifascicular human cervical vagus nerve instrumented with a LivaNova bipolar cuff electrode (Figure F and G). The segmented histology was created using Nikon NIS-Elements.

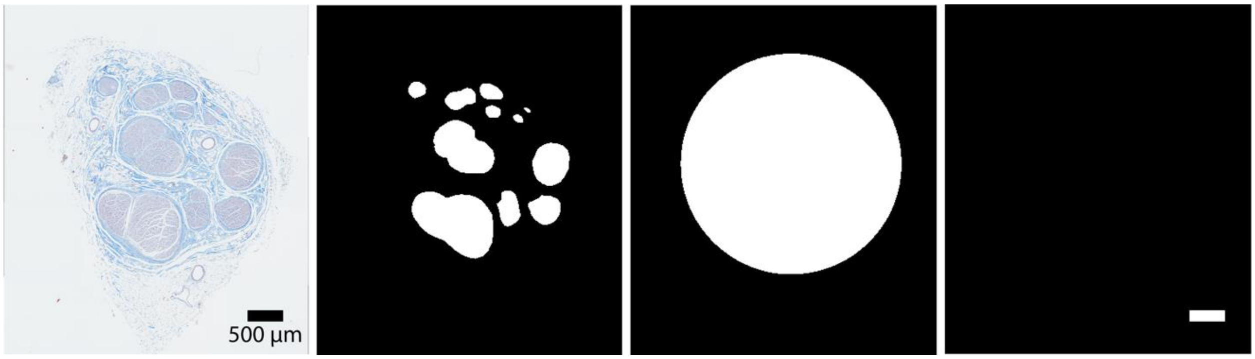

Figure F. Raw histology image (r.tif), segmented inners (i.tif), segmented nerve (n.tif), and scale bar (s.tif) of a human cervical vagus nerve sample that served as inputs to define the cross section of the nerve in the FEM.

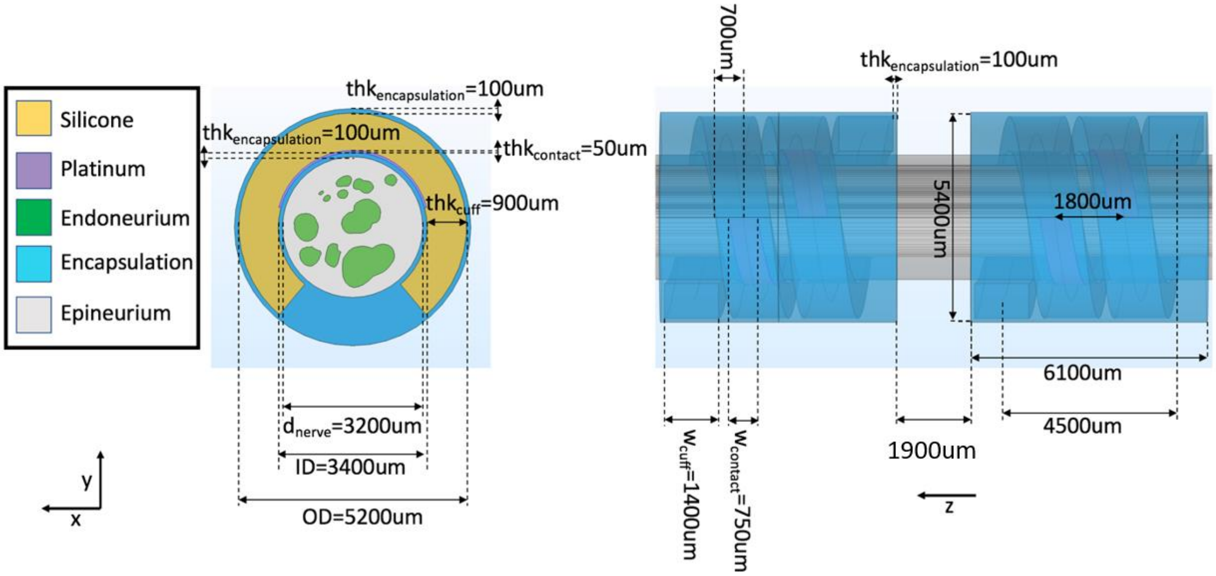

Figure G. FEM of a human cervical vagus nerve sample instrumented with a LivaNova cuff electrode.

The conductivity values applied to the human cervical vagus nerve sample finite element model are provided in Table A, and the boundary conditions applied are provided in Table B.

We seeded 5.7 µm diameter fibers in each fascicle. Between the ASCENT and IT'IS implementations, there was less than 2.5% difference to a single 100 µs duration monophasic rectangular pulse.
