# Supplementary material for: ASCENT (Automated Simulations to Characterize Electrical Nerve Thresholds): A pipeline for sample-specific computational modeling of electrical stimulation of peripheral nerves
Source: PLoS Comput Biol. 2021 Sep 7;17(9):e1009285. doi: 10.1371/journal.pcbi.1009285 (PMC8423288; doi:10.1371/journal.pcbi.1009285)
Supplement: S36 Text — Comparison of MRG fit to Bucksot et al. 2019. (PDF) [file pcbi.1009285.s036.pdf]

1 S36 Text

Appendix. Comparison of MRG fit to Bucksot et al. 2019

1.1 Comparison of MRG fit to Bucksot et al. 2019

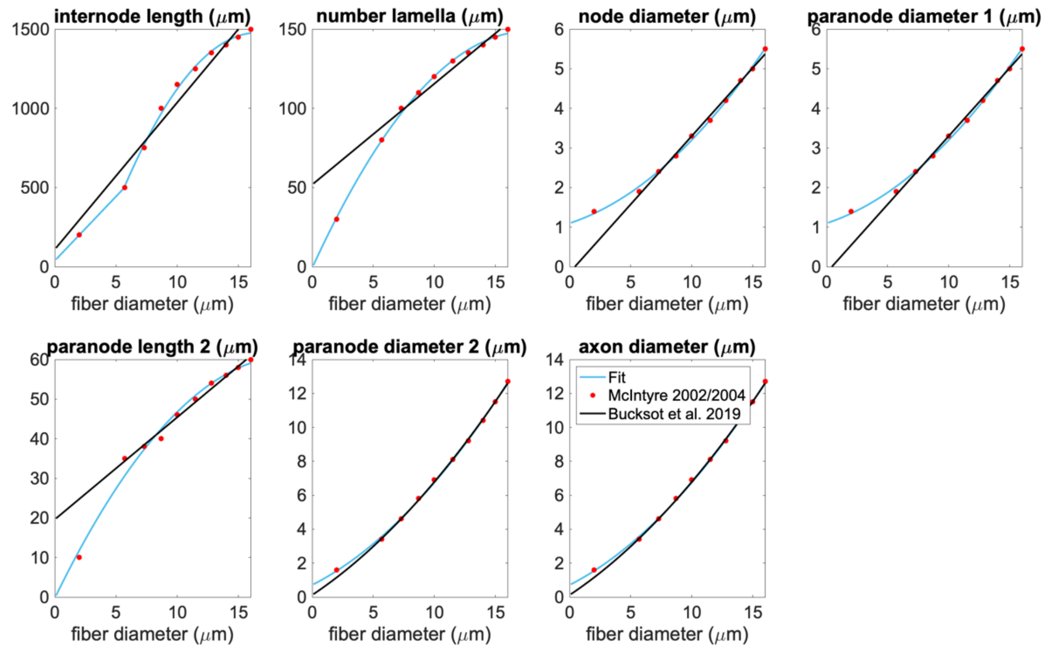

Figure A. Our piecewise polynomial fits to published MRG fiber parameters compared to the Bucksot et al. 2019's interpolation [1]. Single quadratic fits were used for all parameters except for internode length, which has a linear fit below 5.643 μm (using MRG data at 2 and 5.7 μm) and a single quadratic fit at diameters greater than or equal to 5.643 μm (using MRG data >= 5.7 μm); 5.643 μm is the fiber diameter at which the linear and quadratic fits intersected. The fiber diameter is the diameter of the myelin. "Paranode 1" is the MYSA section, "paranode 2" is the FLUT section, and "internode" is the STIN section. The axon diameter is the same for the node of Ranvier and MYSA ("node diameter"), as well as for the FLUT and STIN ("axon diameter"). The node and MYSA lengths are fixed at 1 and 3 μm, respectively, for all fiber diameters.

1.2 References

1. Bucksot JE, Wells AJ, Rahebi KC, Sivaji V, Romero-Ortega M, Kilgard MP, et al. Flat electrode contacts for vagus nerve stimulation. PLoS One [Internet]. 2019;14(11):1–22. Available from: https://doi.org/10.1371/journal.pone.0215191 PMID: 31738766
